# Food selectivity and grazing responses of the Lawnmower Blenny (*Salarias fasciatus*) to nutrient enrichment and ammonia exposure

**DOI:** 10.1111/jfb.70659

**Published:** 2026-09-22

**Authors:** Ramón Alejandro Plazas‐Gómez, Sonia Bejarano, Sara Catalina Rodríguez‐Díaz, Marie Fujitani

**Affiliations:** ^1^ Leibniz Centre for Tropical Marine Research Bremen Germany; ^2^ University of Bremen, Institute for Geography Bremen Germany

**Keywords:** ammonia, blenny, coral reefs, detritivory, eutrophication, grazing

## Abstract

Nutrient enrichment can alter the nutritional quality of the epilithic algal matrix (EAM) and modify the feeding environment of herbivorous and detritivorous reef fishes. However, wastewater inputs can also elevate ammonia concentrations, which may disrupt fish physiological processes, yet the effects of ammonia exposure on grazing activity and feeding selectivity remain poorly understood. In our nutrient enrichment experiment, the nutritional value of the EAM increased under higher nutrient levels, with enriched EAMs containing more lipids, carbohydrates and proteins. The Lawnmower Blenny (*Salarias fasciatus*) grazed more on these nutrient‐enriched tiles, suggesting that feeding preferences were associated with differences in EAM nutritional composition. Ammonia exposure of up to approximately 10 μM did not significantly affect grazing rates, food selectivity or diet composition over the short term. Instead, grazing rates increased over the experimental period after accounting for measured NH_3_ concentration, suggesting behavioural acclimation to the experimental conditions and/or the offered food. These results indicate that, under short‐term and sub‐lethal exposure, feeding behaviour in *S. fasciatus* responded strongly to differences between nutrient‐enriched and low‐nutrient EAMs, while no detectable effect of ammonia on grazing rate was observed. This response may help explain why small benthic detritivorous fishes can persist under moderate nutrient disturbance. Further work is needed to determine whether these responses are maintained under longer exposure periods, higher ammonia concentrations and multi‐stressor conditions.

## 1 INTRODUCTION

Globally, coral reefs are increasingly exposed to agricultural run‐off and wastewater pollution (Tuholske et al., 2021). These inputs deliver nutrients, pathogens and organic matter to coastal ecosystems, with documented impacts on reef biota and ecosystem functioning (Wear & Thurber, 2015). Sewage discharge elevates concentrations of dissolved inorganic nitrogen, including ammonia (NH_3_), a compound known to disrupt cellular processes in fishes by impairing neural transmission, ion regulation and metabolic pathways (Franklin & Edward, 2019). The toxicity of ammonia is strongly influenced by temperature and pH, and to a lesser extent salinity, because these parameters shift the equilibrium between the ionised (NH_4_^+^) and un‐ionised (NH_3_) forms (Clegg & Whitfield, 1995). The un‐ionised form readily diffuses across biological membranes and is therefore primarily responsible for ammonia toxicity in aquatic organisms (Ip & Chew, 2010). Given the typical temperature, pH and variable salinity of tropical marine environments, reef fishes may be particularly vulnerable to ammonia exposure compared with species inhabiting temperate or freshwater systems (Mooney et al., 2018). Despite these environmental conditions, ammonia toxicity in tropical marine fishes has received far less attention than in freshwater species (Randall & Tsui, 2002).

Reef fishes contribute to the resilience of coral reefs through a variety of ecological processes. Among these, large herbivorous reef fishes exert strong top‐down control over algal biomass and therefore play a key role in maintaining coral reef resilience by limiting macroalgal proliferation and reducing the likelihood of phase shifts following disturbance (Hughes et al., 2007; Kuempel & Altieri, 2017). At a finer spatial scale, combtooth blennies (subfamily Salariinae) are abundant small‐bodied cryptobenthic fishes in shallow reef habitats (Townsend & Tibbetts, 2000). Their high feeding frequencies and strong site fidelity result in sustained grazing pressure on epilithic algal matrix (EAM)‐covered surfaces, suggesting that they may influence benthic processes within their immediate feeding areas (Townsend & Tibbetts, 2004). Since EAM is a substrate that mediates benthic productivity and coral recruitment processes (Evensen et al., 2021; Zhang et al., 2025), the disruption of grazing activity under pollution scenarios may alter benthic community trajectories and compromise ecosystem stability (Bellwood et al., 2004).

On polluted reefs, elevated nutrient concentrations may influence the grazing activity of blennies and the ecological functions associated with their feeding. These effects can occur through both direct and indirect pathways. On one hand, the physiological stress may directly reduce grazing efficiency and feeding rates of blennies as ammonia can be lethal at high concentrations, while sub‐lethal exposure has been shown to reduce food intake and impair growth‐related performance in fishes (Parvathy et al., 2023). On the other hand, combtooth blennies are selective grazers that feed intensively on the EAM, consuming mainly detritus (Wilson, 2002). Nutrient enrichment can increase detrital content in the EAM by stimulating algal production and microbial activity, thereby promoting the incorporation of nitrogen into detrital particles within the EAM (Crossman et al., 2001; Tenore et al., 1984). In this context, nutrient enrichment could generate both positive and negative effects on blenny grazing, simultaneously altering food availability while exposing fishes to potentially harmful concentrations of ammonia.

Physiological studies across multiple fish species have shown that chronic ammonia exposure often reduces feed intake and growth performance, processes that are linked to broader metabolic disturbances such as impaired nitrogen excretion and disruptions in neural function (Franklin & Edward, 2019). In juvenile turbot (*Scophthalmus maximus*), elevated concentrations of un‐ionised ammonia reduce growth rates partly through a decline in food intake and decreased efficiency of food utilisation (Rasmussen & Korsgaard, 1996). Similarly, experiments with juvenile cobia (*Rachycentron canadum*) have shown that feeding behaviour can be disrupted at relatively low ammonia concentrations, with fish ceasing to feed before lethal thresholds are reached (Rodrigues et al., 2007). Together, these findings indicate that sub‐lethal ammonia exposure can impair feeding activity well before mortality occurs. However, most of this evidence derives from aquaculture or commercially important species studied under controlled production conditions, whereas comparable information for reef‐associated fishes that perform key ecological functions in coral reef ecosystems remains scarce.

Here we investigate how nutrient enrichment and elevated ammonia concentrations influence benthic grazing by the Lawnmower Blenny *Salarias fasciatus* (Bloch 1786), a common detritivorous reef fish that feeds intensively on the EAM. Building on the direct and indirect pathways outlined above, we test whether ammonia exposure alters blenny feeding activity through physiological effects on the fish, or indirectly through nutrient‐driven changes in the nutritional characteristics of the EAM. Specifically, we ask three questions. First, how does nutrient enrichment modify the nutritional composition of the EAM, in terms of protein, carbohydrate and lipid content? Second, do blennies preferentially graze on EAM that differs in nutritional quality, indicating that food quality influences their feeding behaviour? Third, do sub‐lethal ammonia concentrations in seawater impair food preference, grazing rates or alter diet composition in *S. fasciatus*? In addition, we evaluate whether feeding responses change through time, which may reflect cumulative exposure to ammonia or short‐term acclimation processes. By addressing these questions, our study aims to clarify how nutrient pollution may simultaneously modify food resources and affect the behaviour of small reef grazers, with potential consequences for the ecological functions they perform on coral reefs.

## 2 METHODS

This experiment was conducted at the Marine Ecology Experimental Facility (MAREE) of the Leibniz Centre for Tropical Marine Research (ZMT) between November 2022 and may 2023. All procedures involving animals were carried out in accordance with ethical guidelines and animal welfare standards. Animal husbandry and experimental procedures followed the Regulation for the Welfare Of Experimental Animals issued by the European Commission (EU directive 2010/63) and the Federal Government of Germany. The experiment was approved by the local authorities under animal welfare permit number TVA 179 issued by the Senatorin für Gesundheit, Frauen und Verbraucherschutz, Bremen on December 08, 2022.

### 2.1 Developing epilithic algal matrices (EAM) on artificial substrates

A total of 126 calcium carbonate tiles (12 × 12 cm × 1 cm) were placed in two 500‐L aquaria connected to a 10‐year‐old 3000‐L aquarium system containing several coral and fish species. Tiles were left in the system for two months (Nov ‐ Dec 2022) to allow the settlement and development of EAMs on all surfaces (Figure 1a). During this period, nutrient concentrations were monitored and remained relatively stable, averaging (± standard error [SE]) NO_x_ = 1.51 ± 0.2 μM·L^−1^, NH_4_ = 0.01 ± 0.01 μM·L^−1^ and PO_4_ = 0.34 ± 0.07 μM·L^−1^.

**FIGURE 1:**
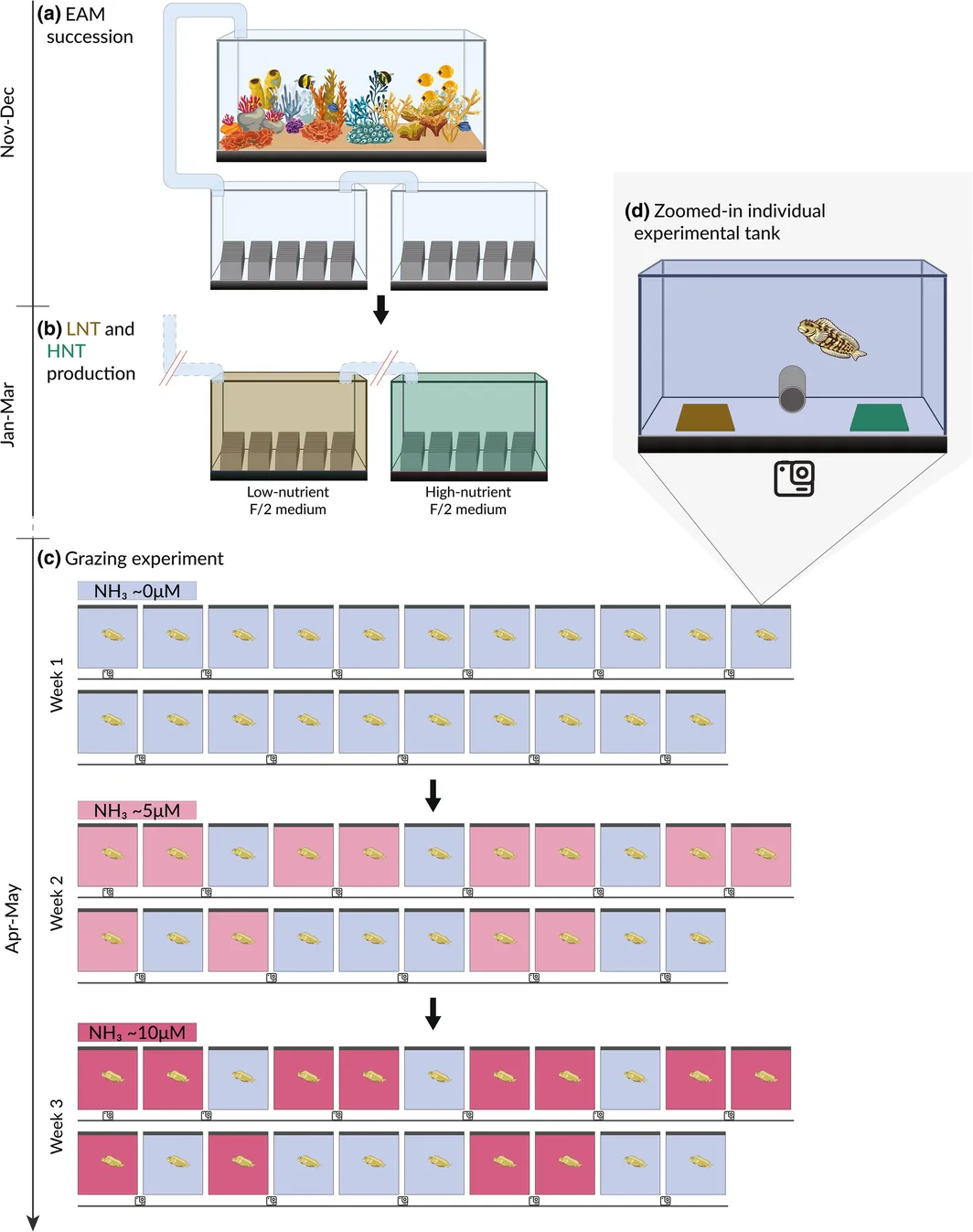
Schematic representation of the experimental design used to evaluate grazing behaviour in *S. fasciatus*. (a) EAM succession phase, in which calcium carbonate tiles were placed in two 500‐L aquaria connected to a 3000‐L coral aquarium system for two months. (b) Nutrient enrichment phase, in which tile aquaria were disconnected and maintained under contrasting nutrient conditions for three months to produce low‐nutrient tiles (LNT) and high‐nutrient tiles (HNTs). (c) Grazing activity experiment, in which 21 *S. fasciatus* individuals were maintained in separate tanks and exposed to increasing seawater NH_3_ concentrations over three weeks. (d) Detail of the individual fish tank during a feeding trial showing the fish, shelter and the simultaneous offer of one LNT and one HNT.

After this succession period, the tile aquaria were disconnected from the coral aquarium and nutrient concentrations were experimentally manipulated using f/2 medium following Guillard and Ryther (1962) to simulate high‐nutrient and low‐nutrient conditions (Figure 1b). The medium was added to the aquaria three times per week for three months (Jan – Mar 2023). This treatment maintained average nutrient concentrations (± SE) of NO_x_ = 18.02 ± 2.43 μM·L^−1^, NH_4_ = 5.47 ± 0.67 μM·L^−1^ and PO_4_ = 7.27 ± 0.67 μM·L^−1^ in the high‐nutrient tank, aiming to simulate nutrient conditions associated with polluted reef environments (Baum et al., 2015; Reopanichkul et al., 2009). In contrast, the low‐nutrient tank maintained concentrations similar to those of the coral aquarium, with average values (± SE) NO_x_ = 1.79 ± 0.37 μM·L^−1^, NH_4_ = 1.72 ± 0.25 μM·L^−1^ and PO_4_ = 0.43 ± 0.08 μM L^−1^. At the end of the three‐month enrichment period, we obtained 63 low‐nutrient tiles (LNTs) and 63 high‐nutrient tiles (HNTs).

### 2.2 Fish acclimation and husbandry

A total of 21 individuals of *S. fasciatus* sourced from DeJong Marine Life (shop.dejongmarinelife.nl) were used in this experiment. Upon arrival on 11 April 2023, fish were acclimated for twelve days in individual 45‐L tanks equipped with air stones and PVC shelters. Water quality parameters (temperature, salinity, pH, O_2_, NO_2_) were monitored and kept stable in each tank throughout the acclimation period and the experiment (Table S1). In addition, the body condition (including body shape and the condition and colouration of skin and fins) and behaviour of each fish were monitored daily during both the acclimation and experimental periods. During the behavioural observations, we did not find signs of erratic swimming, lethargy or persistent refusal to feed in the fish used in the experiment. Fish were weighed and total size and standard size were measured at the end of the experiment.

Daily at 10:00, three water samples were taken from each aquarium. Salinity, dissolved oxygen, temperature and pH were measured using a WTW MultiLine F/Set3 multi‐parameter probe. Nutrient samples were immediately filtered using 0.45‐μm syringe filters (Sartorius Minisart®) and transferred to pre‐rinsed polyethylene bottles. Ammonium samples were frozen at −20°C. Concentrations of NH_4_^+^ were quantified spectrophotometrically using a TECAN Infinite Pro M200 plate reader. The un‐ionised ammonia concentration (NH_3_ μM) in each fish tank was calculated from the measured total ammonium concentration (NH_4_^+^ + NH_3_) and in situ physicochemical conditions following the thermodynamic formulation of Clegg and Whitfield (1995).

### 2.3 Grazing activity experiment

Fish were maintained in their individual tanks during the experimental phase (Figure S1). The grazing activity experiment was conducted over three weeks, from 24 April to 11 May 2023 (Figure 1c,d). During the first week, all 21 fish were maintained under baseline conditions without ammonia addition. In the second week, 12 of those individuals, randomly selected, were exposed to target ammonia concentrations of approximately 5 μM NH_3_, while the remaining nine individuals served as controls without ammonia addition. In the third week, ammonia concentration in the 12 treatment tanks was increased to approximately 10 μM NH_3_, while the same control group (*n* = 9) remained unexposed. Ammonia was added once to the treatment tanks on Mondays, 24 h before the first feeding observations of weeks 2 and 3.

Each week consisted of three phases: starvation, feeding trials and rest. (I) Starvation: Fish were starved for 24 h (Mondays) to standardise hunger levels; (II) Feeding trials: For three consecutive days (Tuesdays–Thursdays), fish were recorded once per day for one hour (13:00–14:00). This time window was selected based on the diel feeding hypothesis, which predicts that feeding activity in many reef fishes, including *S. fasciatus*, peaks during the afternoon (Wilson, 2009). During each trial, fish were simultaneously offered one LNT and one HNT, and grazing activity was recorded using high‐definition video cameras (GoPro®). The tiles were removed after recording. A total of 126 tiles (63 LNT and 63 HNT) were prepared, allowing each tile to be used only once per experimental week. The same set of tiles was reused across weeks, but tile assignment to individual fish was randomised so that no fish was repeatedly offered the same pair of tiles. All trials were recorded simultaneously for all fish using one camera per two tanks (Figures 1c,d, S1a); (III) Rest: On days when feeding behaviour was not recorded (Fridays–Sundays), fish were fed dried seaweed (Ocean Nutrition®). The two ammonia concentrations used in the experiment were selected to simulate intermediate and high pollution levels reported for coastal marine environments (Baum et al., 2015; Reopanichkul et al., 2009).

### 2.4 EAM nutritional content

On May 11, following the last feeding trial, 42 randomly selected tiles (21 LNT and 21 HNT) were collected, and their EAMs were removed using a scalpel blade. The samples were maintained at −20°C until laboratory analysis. This consisted of homogenising and dividing the samples for lipid (chloroform–methanol extraction), protein (Bradford assay) and carbohydrate (phenol–sulfuric acid assay) quantification to assess nutritional composition, using a standardised cellular energy allocation (CEA) protocol adapted from De Coen and Janssen (1997) and Verslycke et al. (2004). Absorbance was measured at 375 nm (lipids), 592 nm (proteins) and 492 nm (carbohydrates), and concentrations were calculated using standard curves. Values were normalised per unit of wet biomass (WW) in mg g^−1^.

### 2.5 Video analysis

Videos were played back in the laboratory to count the number of bites taken by each individual fish per tile type in each assay.

### 2.6 Blenny diet composition analysis

On May 11, following the last feeding trial, fish were euthanised by a trained scientist using tricaine mesylate (MS‐222) in accordance with the guidelines from The American Veterinary Medical Association (AVMA) and the Federation of European Laboratory Animal Science Associations (FELASA). Fish were dissected and their stomachs frozen at −20°C. Later, the stomach samples were thawed and analysed using a digital microscope (Keyence VHX 5000). Contents were diluted in water in a Petri dish placed over a millimetric grid. Each food item was classified into one of six categories: detritus, green algae, brown algae, red algae, cyanobacteria or bivalves. Relative contributions of each category were estimated visually using the millimetric grid as a guide.

### 2.7 Data analysis

To test whether high nutrient concentration in seawater led to EAMs of higher nutritional quality, linear models were fitted for each nutritional variable with treatment as a fixed factor. Model assumptions were evaluated through visual inspection of residual diagnostic plots. Where necessary, response variables were log‐transformed to improve normality and homoscedasticity.

To determine whether blennies grazed differently on EAMs with contrasting nutritional composition, and whether grazing activity was associated with ammonia exposure or experimental time, grazing behaviour was analysed using a generalised linear mixed‐effects model. Bite counts on individual HNT and LNT tiles were modelled with tile type, measured NH_3_ concentration and experimental time as fixed effects. Experimental time was expressed as the number of calendar days since the beginning of the experiment and centred around its mean. Recording duration was incorporated as a log‐transformed offset to account for small differences in observation time (65.3 ± 0.9 min, SE), thereby modelling grazing rate per minute. Fish identity was included as a random intercept to account for repeated observations of the same individuals, and Trial ID was nested within Fish ID to account for the paired HNT and LNT observations obtained within each feeding trial and for additional trial‐to‐trial variation.

Poisson, negative‐binomial type 1, negative‐binomial type 2 and zero‐inflated versions of both negative‐binomial distributions were evaluated using a log link. Model selection was based on Akaike information criterion (AIC) and simulation‐based residual diagnostics. The negative‐binomial type 1 model was retained because it had the lowest AIC among models with reliable convergence and showed satisfactory residual diagnostics.

As a supplementary assessment of food selectivity, the relative allocation of bites between HNT and LNT tiles was analysed at the trial level using the paired numbers of bites on each tile as the response. Trials in which no bites were recorded on either tile were excluded because they contained no information about relative bite allocation. Measured NH_3_ concentration and centred experimental time were included as continuous fixed effects, and fish identity was included as a random intercept. Binomial and beta‐binomial error structures with a logit link were compared, and the beta‐binomial model was retained based on model fit and residual diagnostics (Supporting Information S1).

Model diagnostics were evaluated using the DHARMa package (version 0.4.7), including tests of residual uniformity, dispersion and zero inflation. Full results and diagnostics for all candidate models and the supplementary food‐selectivity analysis are provided in the Supporting Information S1. All models were fitted using the glmmTMB package in R version 4.5.2.

Fulton's condition factor (K) was estimated as a proxy for body condition and calculated as follows:

$$K=100\times \frac{\text{Weight}}{{\text{Length}}^{3}}$$

Differences in Fulton's *K* between ammonia‐exposed and control fish were evaluated using a Student's *t*‐test.

Diet composition was summarised by calculating the mean (± SE) relative contribution (%) of each food item across all individuals and separately for each treatment group. To visualise multivariate differences in diet composition between ammonia‐exposed and control fish, we performed a non‐metric multidimensional scaling (NMDS) using Bray–Curtis dissimilarities. Differences in overall diet composition between ammonia‐exposed and control fish were tested using a permutational multivariate analysis of variance (PERMANOVA; 999 permutations) based on Bray–Curtis dissimilarities. Dietary overlap between ammonia‐exposed and control fish was quantified using Pianka's niche‐overlap index (Pianka, 1973), which ranges from 0 (no overlap) to 1 (complete overlap). All analyses were conducted using the R package vegan (version 2.7–2).

## 3 RESULTS

### 3.1 Nutrient enrichment enhances EAM nutritional quality

The EAMs developed on HNT contained significantly higher concentrations of lipids, carbohydrates and proteins than those developed on LNT (*p* < 0.001; Figure 2). Mean (± SE) lipid, carbohydrate and protein contents in HNT were 6.28 ± 0.38, 5.60 ± 0.47 and 9.07 ± 0.98 mg g^−1^ WW, respectively. In contrast, LNT contained lower concentrations of these components, averaging 3.60 ± 0.13 mg g^−1^ lipids, 3.29 ± 0.28 mg g^−1^ carbohydrates and 2.48 ± 0.28 mg g^−1^ proteins.

**FIGURE 2:**
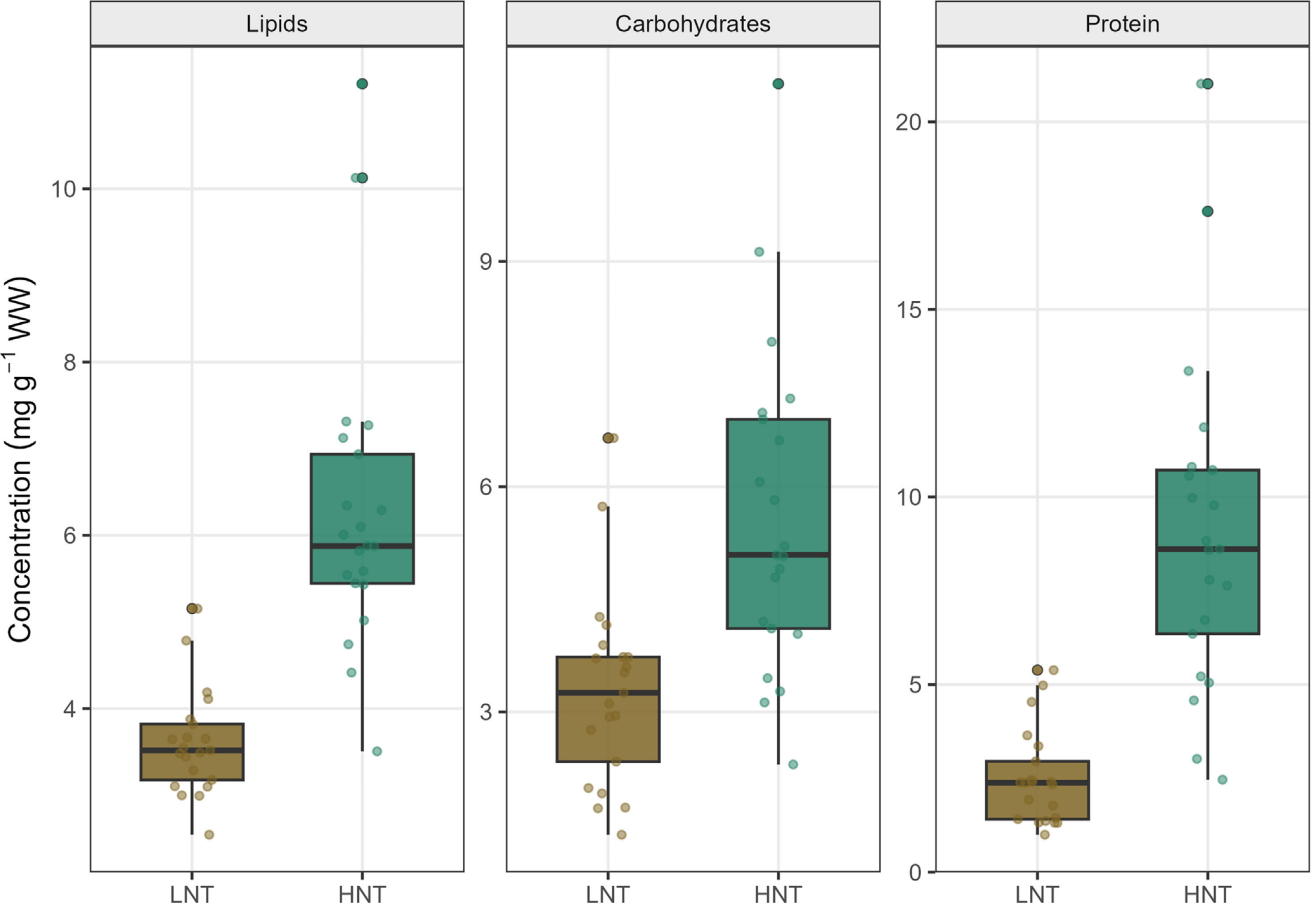
Lipid, carbohydrate and protein concentrations (mg g^−1^ wet weight, WW) of the epilithic algal matrix (EAM) scraped from high‐nutrient tiles (HNTs) and low‐nutrient tiles (LNT). Boxes represent the interquartile range (25th–75th percentiles), the horizontal line indicates the median and whiskers extend to the most extreme values within 1.5 times the interquartile range. *n* = 21 tiles per treatment.

### 3.2 Effect of nutritional value and ammonia exposure on S. fasciatus grazing rates

Blennies showed higher grazing rates on HNT than on LNT. The tile type had a strong positive effect on grazing rate (*β* = 1.024, SE = 0.099, *z* = 10.37, *p* < 0.001; Table 1; Figure 3a). The corresponding rate ratio was 2.78 (95% CI: 2.29–3.38), indicating that the expected grazing rate on HNT was approximately 2.8 times that on LNT.

**TABLE 1:** Summary of the negative‐binomial type 1 generalised linear mixed‐effects model used to analyse grazing behaviour in *Salarias fasciatus*.

Negative binomial model				
Term	Estimate	Std. error	*z* value	Pr(>|*z*|)
(Intercept)	−1.463	0.297	−4.922	8.55e−07
Tile type	1.023	0.099	10.368	<2e−16
NH_3_ (μM)	0.028	0.043	0.650	0.516
Experimental time	0.047	0.017	2.723	0.006

random effects	Variance	Std. Dev.
Trial ID:Fish (Intercept)	0.694	0.833
Fish (Intercept)	1.164	1.079

*Note*: Tile‐level bite counts were modelled as a function of tile type (LNT as the reference level), measured NH_3_ concentration and experimental time. Recording duration was included as a log‐offset to express grazing rates per minute. Fish identity was included as a random intercept, with Trial ID nested within Fish to account for repeated observations of individuals and the paired HNT and LNT observations within each feeding trial. Fixed‐effect estimates are reported with standard errors (SEs), Wald *z*‐values and associated *p*‐values; variance and standard deviation are reported for random effects. The model was fitted to 374 tile‐level observations from 187 feeding trials involving 21 individuals.

**FIGURE 3:**
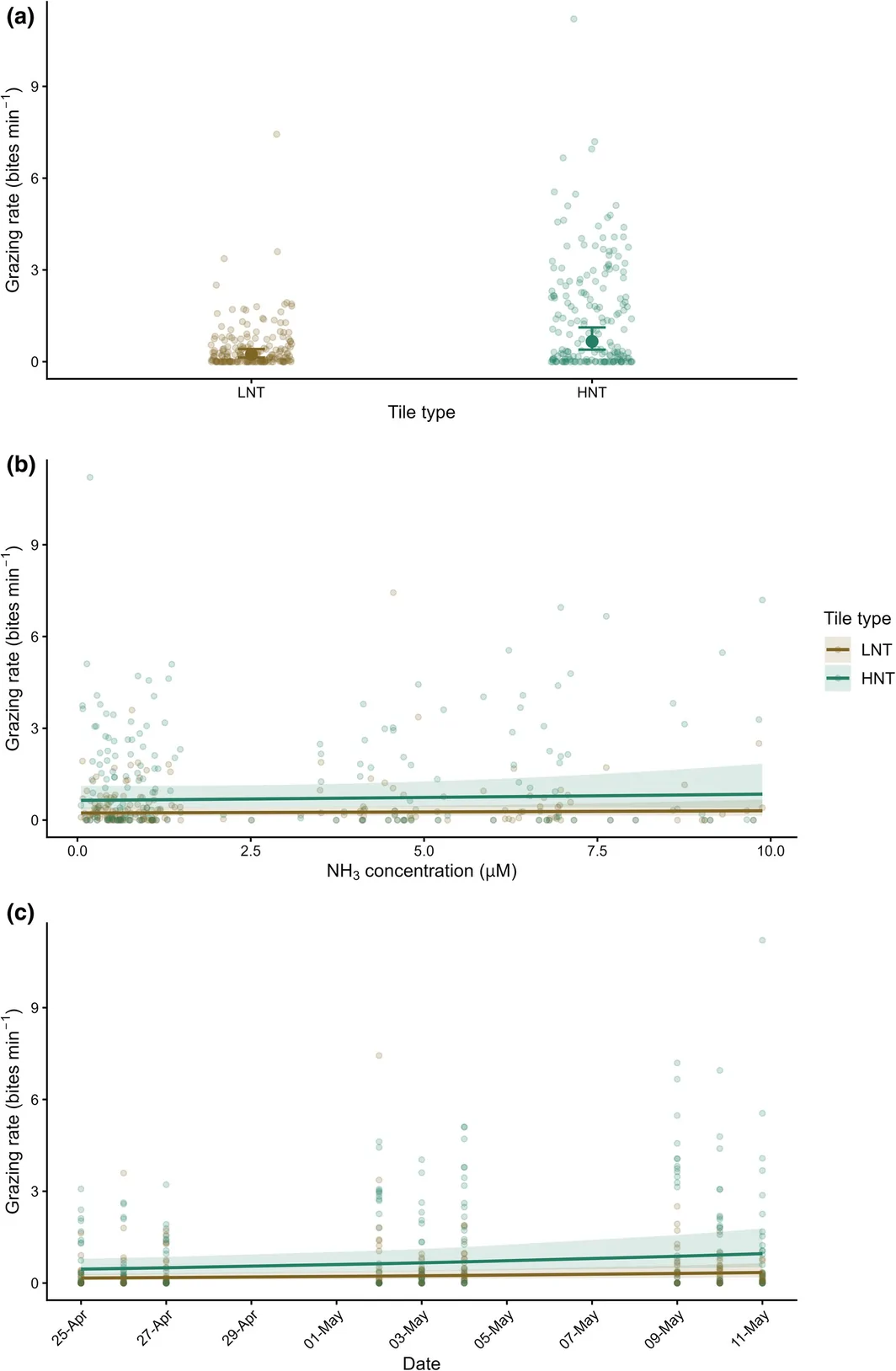
Observed and model‐predicted grazing rates (bites min^−1^) of *Salarias fasciatus* on low‐nutrient (LNT) and high‐nutrient tiles (HNTs). (a) Observed tile‐level grazing rates and model‐predicted mean grazing rates for LNT and HNT, with NH_3_ concentration held at its median and experimental time at its mean. (b) Predicted grazing rates across the observed NH_3_ concentration gradient, with experimental time held at its mean. (c) Predicted grazing rates across the experimental period, with NH_3_ concentration held at its median. Points represent observed tile‐level grazing rates, solid lines or symbols represent population‐level model predictions, and shaded ribbons or error bars indicate 95% confidence intervals.

NH_3_ concentration was not significantly associated with grazing rate (*β* = 0.028, SE = 0.043, *z* = 0.65, *p* = 0.516; rate ratio = 1.03, 95% CI: 0.95–1.12; Figure 3b). Although each increase of 1 μM NH_3_ corresponded to an estimated 2.8% increase in grazing rate, the confidence interval included no effect. In contrast, grazing rate increased significantly with experimental time *(β* = 0.047, SE = 0.017, *z* = 2.72, *p* = 0.006; Figure 3c), corresponding to an estimated 4.8% increase per elapsed day (rate ratio = 1.05, 95% CI: 1.01–1.08).

Considerable variation in grazing activity occurred both among individuals and among feeding trials within individuals. The variance associated with fish identity was 1.16, with a standard deviation of 1.08 on the log scale, whereas the variance associated with Trial ID nested within Fish ID was 0.69, with a standard deviation of 0.83 (Table 1). These standard deviations correspond to approximately 2.9‐fold and 2.3‐fold multiplicative differences in expected grazing rate per standard deviation at the fish and trial levels, respectively.

In the food‐selectivity model, bites recorded during active feeding trials were predominantly allocated to HNT, with an estimated probability of 72% (95% CI: 66–78%). This relative allocation of bites was not significantly associated with NH_3_ concentration (*β* = 0.032, SE = 0.039, *p* = 0.409; odds ratio = 1.03, 95% CI: 0.96–1.11) or experimental time (*β* = 0.013, SE = 0.017, *p* = 0.423; odds ratio = 1.01, 95% CI: 0.98–1.05; Supporting Information S1).

### 3.3 Gut contents and body condition of S. fasciatus

Six food categories were identified in the gut contents of *S. fasciatus*: detritus, green algae, brown algae, red algae, cyanobacteria and bivalves. Detritus and green algae dominated the diets of both ammonia‐exposed and control fish (Figure 4a). Overall diet composition did not differ significantly between treatments (*p* = 0.324). Nevertheless, ammonia‐exposed fish showed a higher mean relative contribution (mean ± SE) of detritus in their gut contents (50.5 ± 8.0%) than control fish (38.0 ± 6.7%), whereas control fish showed a higher mean relative contribution of green algae (46.3 ± 8.7%) than exposed fish (39.6 ± 8.2%). The remaining food categories contributed relatively small proportions to the diet, generally differing by less than 2% between treatments, except for bivalves, which showed a higher mean relative contribution in control fish (8.8 ± 2.2%) than in ammonia‐exposed fish (4.8 ± 2.1%).

**FIGURE 4:**
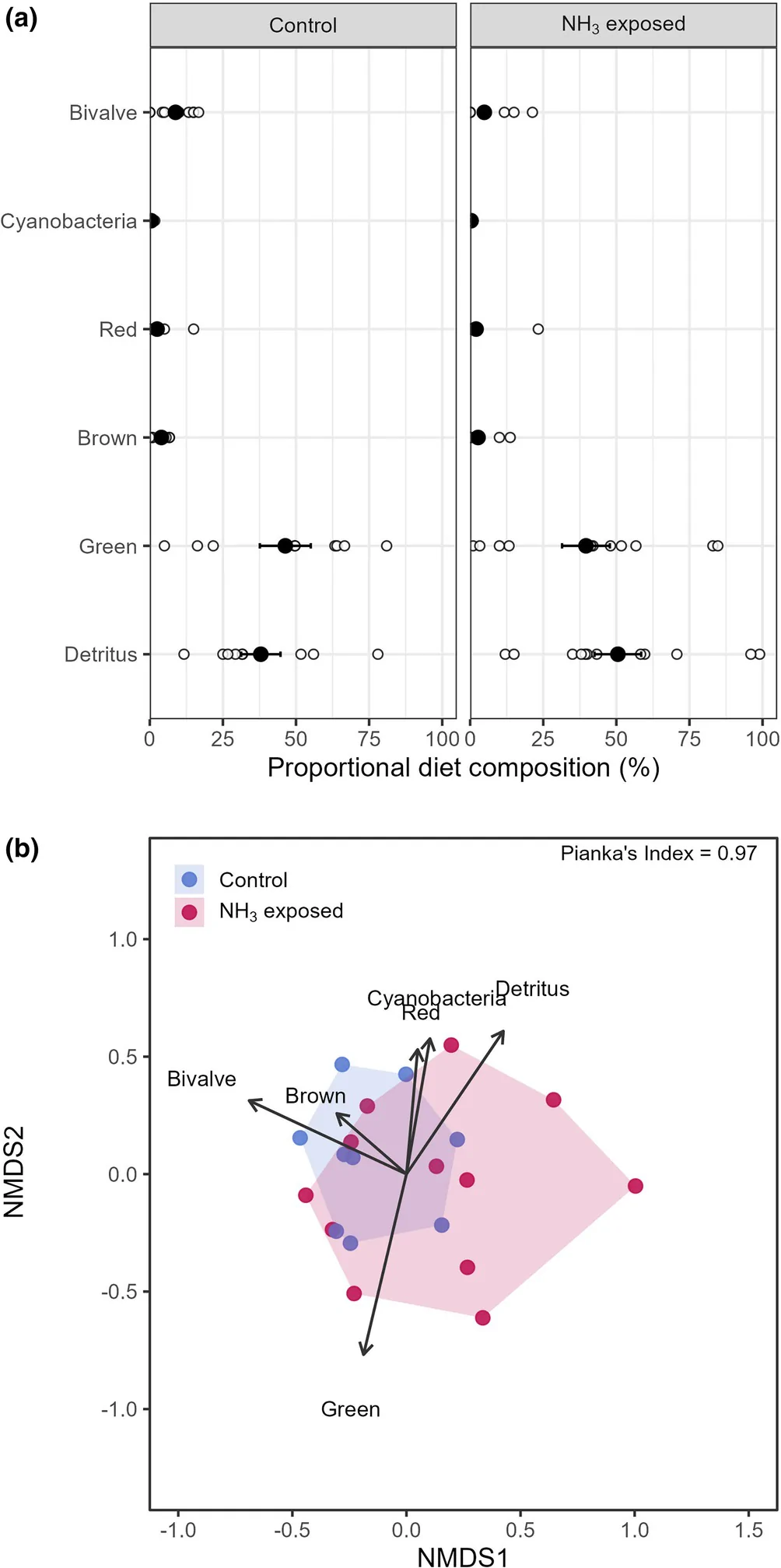
Diet composition and multivariate representation of gut contents in *S. fasciatus* individuals exposed to ~10 μM NH_3_ and not exposed (Control). (a) Proportional diet composition (%) of individual fish. Open circles (○) represent individual observations and filled circles (•) indicate mean values ± SE for each food category. (b) Non‐metric multidimensional scaling (NMDS) ordination based on gut content composition of *S. fasciatus*. Points represent individual fish, and convex hull polygons outline the multivariate dietary space occupied by each group. Arrows indicate the direction and relative contribution of each food item to the ordination.

All six food items were present in the guts of both exposed and control fish, indicating a high dietary overlap between the two groups (Pianka's index = 0.97) (Figure 4b). However, diet composition appeared more variable among ammonia‐exposed fish than among control individuals. This variability was largely driven by some individuals whose gut contents were dominated almost entirely by detritus or green algae, with little or no contribution from other items such as brown algae or bivalves. At the end of the experiment, ammonia‐exposed fish exhibited a significantly higher body condition (Fulton's K) than control fish (mean ± SE: 0.99 ± 0.04 vs. 0.87 ± 0.03; *t*‐test, *p* = 0.026).

## 4 DISCUSSION

This study examined how nutrient enrichment and sublethal ammonia exposure influence the grazing behaviour of the detritivorous blenny *S. fasciatus*. Our results reveal three main patterns. First, nutrient enrichment increased the biochemical quality of the EAM, with higher lipid, carbohydrate and protein contents in HNT compared with LNT. Second, blennies grazed more intensively on HNT, indicating that food quality may influence feeding activity. Third, sublethal ammonia exposure (up to 10 μM NH_3_) was not significantly associated with grazing rate or relative bite allocation, and diet composition did not differ between ammonia‐exposed and control fish. Grazing rates increased significantly over experimental time, potentially reflecting behavioural acclimation to the experimental conditions and/or the offered food. These findings indicate that differences between nutrient‐enriched and low‐nutrient EAMs strongly influenced the feeding behaviour of *S. fasciatus*, while grazing activity was maintained during moderate, short‐term ammonia exposure.

### 4.1 Nutrient enrichment enhances EAM nutritional quality

The significantly higher lipid, carbohydrate and protein concentrations measured in HNT confirm that nutrient enrichment alters the biochemical composition of the EAM. Similar responses have been documented in other algal systems, where increased nutrient availability enhances carbohydrate accumulation, particularly insoluble sugars (Hemmi & Jormalainen, 2002). Detritus in EAM forms through algal decay and the accumulation of organic matter (Tenore et al., 1984; Wilson, 2002), while microbial processing can increase its nutritional value and palatability (Wilson et al., 2001, 2003). Therefore, nutrient enrichment may increase the energetic value of EAM food resources through changes in algal, detrital and microbial components. Under moderate enrichment such as that tested here, this may increase food quality for detritivorous reef fishes; however, any benefit to consumers will depend on how enrichment simultaneously alters the structure and composition of benthic communities, including shifts towards longer algal turfs and increased coral–algal competition (Lin et al., 2021).

### 4.2 Nutritional value as a driver of food selectivity in *S. fasciatus*

Our results show that *S. fasciatus* had higher grazing rates on HNT than on LNT, suggesting that differences in the nutritional value of the EAM may influence feeding selectivity in this species. Although both tile types supported well‐developed EAM communities, they differed markedly in biochemical composition, with higher lipid, carbohydrate and protein contents in HNT. The consistently higher grazing rates on HNT, together with the greater relative allocation of bites to these tiles, suggest that *S. fasciatus* may discriminate among feeding patches based on their energetic value. However, nutrient enrichment may also have increased the quantity of edible material, particularly detritus, available on HNT. Consequently, the higher grazing rates observed on these tiles may reflect not only differences in nutritional quality but also differences in food availability. In addition, although each tile was used only once per experimental week, repeated grazing across weeks may have influenced EAM turnover and recovery, as grazing can modify turf biomass, sediment loads and the composition and productivity of EAM‐associated resources (Marshell & Mumby, 2015; Robinson et al., 2018; Tebbett et al., 2018).

Previous studies have suggested that salariin blennies do not feed randomly within the EAM but instead target patches that provide higher energetic returns. In field experiments, Wilson (2009) found that blennies fed more frequently in EAM patches where sediments and associated detritus remained than in patches from which these components had been removed and interpreted this pattern as evidence that fish preferentially targeted more nutritious food resources. However, the nutritional advantage of detritus may not depend simply on its abundance. Biochemical analyses have shown that the nutritional composition of detrital and algal components can vary substantially with their origin and may, in some cases, be broadly similar (Wilson, 2002; Wilson et al., 2003). This suggests that the quality of detrital and algal material, rather than the amount of detritus alone, may be important in determining the energetic value of EAM feeding patches. Our experimental results support this interpretation by showing that higher nutritional quality of the EAM was associated with higher grazing rates in *S. fasciatus*.

In this context, food selectivity is likely functionally important for these small‐bodied reef fishes. These species typically exhibit high mass‐specific metabolic rates (Clarke & Johnston, 1999), which require frequent feeding and efficient energy acquisition. Under these physiological constraints, targeting nutritionally dense and readily digestible fractions of the EAM would reduce processing costs and maximise net energetic gain (Wilson, 2009).

### 4.3 Short‐term responses of *S. fasciatus* to ammonia in seawater

Sub‐lethal ammonia exposure was not significantly associated with grazing rate, relative bite allocation or diet composition in *S. fasciatus*, whereas grazing rates increased over the experimental period. The temporal increase may reflect acclimation to the experimental conditions and/or the offered food rather than a direct response to ammonia. This pattern contrasts with our initial expectation. Elevated ammonia in water is generally expected to reduce food intake and growth because it hinders branchial ammonia excretion and increases the physiological cost of maintaining internal ammonia homeostasis (Bucking, 2017; Ip & Chew, 2010). As external ammonia rises, fish may need to invest more energy into defence mechanisms such as active NH_4_^+^ excretion, ionoregulatory adjustments and temporary detoxification, thereby reducing the energy available for growth and, in some cases, feeding (Bucking, 2017; Schram et al., 2010). However, the absence of feeding depression in our study highlights that responses to elevated ammonia vary markedly among fish species and depend strongly on their intrinsic defence capacity (Ip & Chew, 2010).

The increase in grazing observed across the experiment is more consistent with a temporal response, potentially including acclimation to the experimental conditions, than with a direct effect of ammonia. Nevertheless, the absence of feeding depression does not rule out physiological costs that were compensated for without producing a detectable decline in grazing. In some fish, increased feeding has been interpreted as compensation for the energetic costs of maintaining ammonia homeostasis. In African catfish, for example, feed intake was elevated at 14–38 μM NH_3_, which was interpreted as likely overcompensation for the energetic cost of defending ammonia homeostasis (Schram et al., 2010). In rainbow trout, feeding initially declined during the first 48 h of sublethal ammonia exposure but increased again thereafter, a pattern interpreted as recovery from the initial stress response and re‐establishment of ammonia equilibrium (Wicks & Randall, 2002). By contrast, in European sea bass, the dominant response during exposure was reduced food intake, although once ammonia was removed fish displayed hyperphagia, consistent with compensatory feeding after growth restriction (Dosdat et al., 2003). These contrasting responses indicate that the effects of ammonia depend not only on concentration, but also on exposure duration and species‐specific capacity to regulate and detoxify ammonia.

This study suggests that *S. fasciatus* may maintain food selectivity and grazing activity during short‐term ammonia exposure up to 10 μM NH_3_. This does not imply that ammonia failed to trigger physiological responses, but rather that any associated regulatory costs were not sufficiently strong to cause a detectable reduction in feeding under the experimental conditions. Importantly, higher concentrations and/or longer exposure periods may still depress feeding of *S. fasciatus*, as reported for other fishes (Franklin & Edward, 2019; Rasmussen & Korsgaard, 1996; Rodrigues et al., 2007). Therefore, our results should not be interpreted as evidence that blennies are unaffected or benefit from ammonia or nutrient enrichment in the marine environment.

### 4.4 Diet composition and body condition

Diet composition appeared more variable among ammonia‐exposed fish, although overall composition did not differ significantly between groups. Ammonia‐exposed fish also had a higher Fulton's K at the end of the experiment. Because body condition was not measured before exposure, this difference cannot be attributed to ammonia treatment and may reflect pre‐existing variation among individuals. These descriptive patterns therefore provide no direct evidence of compensatory feeding but identify responses that could be examined through repeated measurements of body condition and physiological status in future experiments.

Additionally, the gut contents observed in our experiment were broadly consistent with previous studies of wild salariin blennies, in which detritus constitutes the dominant dietary component, followed by filamentous algae and small proportions of invertebrates (Quimbayo et al., 2020; Wilson, 2000). However, the relative contribution of detritus in our study (38%–50%) was lower than that reported in those studies, where it typically exceeds 80%. This difference may reflect the simplified EAM communities that developed under laboratory conditions and is consistent with the results of Christiansen et al. (2010), who reported that *S. fasciatus* can remove up to 57% of photosynthetic material from artificial substrata with a developed EAM layer.

### 4.5 Ecological implications, limitations and future directions

Grazing rate was not significantly associated with measured NH_3_ concentration, while ammonia‐exposed fish showed similar diet composition to control individuals (Figures 3b,c and 4a), suggesting that *S. fasciatus* maintained grazing activity and similar diet composition under the nutrient conditions tested. This is consistent with trait‐based patterns observed in wastewater‐impacted reef systems, where smaller‐bodied fishes at lower trophic levels were less negatively associated with pollution gradients than larger predators and high‐trophic‐level taxa (Plazas‐Gómez et al., 2025, 2026). In that context, traits such as small body size, benthic feeding mode and lower trophic level were linked to persistence under chronic wastewater influence. Our experimental results suggest one potential mechanism contributing to this pattern, as *S. fasciatus* maintained grazing activity and relative bite allocation during moderate, short‐term ammonia exposure.

However, these results should be interpreted within the limits of the experimental design. The study tested short‐term exposure under controlled laboratory conditions, whereas reefs affected by wastewater or eutrophication are exposed to more complex and persistent disturbances. Chronic exposure, higher ammonia concentrations or interaction with additional stressors such as hypoxia, thermal stress and pathogen load could reduce this apparent resilience (Dosdat et al., 2003; Franklin & Edward, 2019; Ip & Chew, 2010). In parallel, nutrient enrichment can alter benthic community structure, including shifts in EAM composition or increased macroalgal dominance (Adam et al., 2021), which may indirectly modify feeding dynamics beyond the direct effects of ammonia alone.

An additional limitation relates to the EAMs used in this experiment. Although the nutrient enrichment treatment generated clear differences in the biochemical composition of the EAM, the communities that developed under laboratory conditions likely represent a simplified version of natural reef EAMs. Natural EAMs are structurally and taxonomically complex and are shaped by a broader range of anthropogenic pressures, environmental processes and biological interactions than can be reproduced in laboratory aquaria (Evensen et al., 2021; Zhang et al., 2025). These constraints should be considered when extrapolating these findings to natural reef systems. Future studies using naturally developed EAMs would help determine whether the feeding preferences and grazing responses observed here are maintained under more realistic ecological conditions.

In conclusion, nutrient enrichment increased the nutritional quality of the EAM by raising its protein, lipid and carbohydrate contents, and *S. fasciatus* exhibited substantially higher grazing rates on HNT than on LNT. During active feeding trials, fish also allocated a greater proportion of their bites to HNT, while this relative allocation was not significantly associated with ammonia concentration or experimental time. Grazing rates increased over the experimental period, potentially reflecting acclimation to the experimental conditions and/or the offered food, whereas ammonia concentration was not significantly associated with grazing rate. Although *S. fasciatus* maintained grazing activity during short‐term exposure to moderate ammonia concentrations, further studies are needed to assess its responses to higher concentrations, longer exposure periods and additional pollutants under both natural and laboratory conditions.

## AUTHOR CONTRIBUTIONS

S.B., M.F. and R.A.P.G. conceived the study. R.A.P.G. and S.C.R.D. conducted the laboratory work, collected and analysed the data. All authors contributed to writing the manuscript and approved the final version.

## FUNDING INFORMATION

This research was funded by the German Federal Ministry of Education and Research (BMBF) through the TransTourism project (Grant code 01UU0907).

## CONFLICT OF INTEREST STATEMENT

The authors declare no conflicts of interest.

## Supporting information


**Table S1.** Mean, standard deviation (SD) and standard error (SE) of water quality parameters monitored in the individual tanks from the start of fish acclimation (11 April 2023) to the end of the experiment (11 May 2023). Parameters include nitrite concentration (NO_2_^−^), salinity, temperature (T), oxygen saturation (%), dissolved oxygen concentration (O_2_) and pH.
**Figure S1.** Experimental setup used for the grazing activity trials at the Marine Ecology Experimental Facility (MAREE), Leibniz Centre for Tropical Marine Research (ZMT). (a) Overview of the individual fish tanks and camera arrangement used to record grazing behaviour during the experiment. (b) Close‐up of a single experimental tank showing one Salarias fasciatus individual, the two experimental tiles, the PVC shelter and the air stone.

**Supporting Information S1:** Extended statistical analyses of *Salarias fasciatus* grazing activity and food selectivity, including data preparation, candidate‐model comparison, simulation‐based residual diagnostics, effect‐size estimation and model‐predicted grazing rates.

## Data Availability

The data that supports the findings of this study are available in the Supporting Information of this article.
