# Supplementary material for: Food selectivity and grazing responses of the Lawnmower Blenny (Salarias fasciatus) to nutrient enrichment and ammonia exposure
Source: J Fish Biol. 2026 Sep 22;109(5):2851–62. doi: 10.1111/jfb.70659 (PMC13770696; doi:10.1111/jfb.70659)
Supplement: Supplementary file 1 [file JFB-109-2851-s002.pdf]

Supplementary Methods S1: Grazing activity and food selectivity

R. Alejandro Plazas-Gómez

2026-09

Contents

1. Overview **2**

2. Data import and preparation **2**

2.1 Basic data checks . . . 2

2.2 Trial identifier and experimental time . . . 3

2.3 Tile-level dataset for the primary grazing analysis . . . 3

2.4 Trial-level dataset for the complementary selectivity analysis . . . 4

3. Tile-level analysis of grazing activity **5**

3.1 Candidate model set . . . 5

3.2 Model selection . . . 6

3.2.1 AIC . . . 6

3.2.2. DHARMa diagnostics . . . 6

4. Negative-binomial type 1 grazing model **31**

4.1 Fixed-effect rate ratios and 95% confidence intervals . . . 32

5. Model-predicted grazing rates **33**

5.1 Population-level prediction function . . . 33

5.2 Panel A: HNT versus LNT grazing rates . . . 33

5.3 Panel B: grazing rate across ammonia concentration . . . 36

5.4 Panel C: grazing rate across experimental time . . . 37

5.5 Combined figure . . . 39

6. Food-selectivity analysis **41**

6.1 Binomial and beta-binomial candidate models . . . 41

6.2 DHARMa diagnostics . . . 42

6.3 Selected beta-binomial model . . . 50

6.4 Odds ratios and HNT allocation probability . . . 51

1. Overview

This supplementary material provides the full analytical workflow used to examine grazing activity and food selectivity in Salarias fasciatus. Two complementary analyses were conducted to distinguish between absolute grazing rates on individual tiles and the relative allocation of bites between simultaneously offered food resources.

Grazing activity was analysed at the tile level using generalized linear mixed-effects models. Bite counts on high-nutrient tiles (HNT) and low-nutrient tiles (LNT) were modelled as a function of tile type, measured NH3 concentration, and experimental time. Fish identity and feeding trial were incorporated as nested random effects to account for repeated observations of individuals and the paired HNT and LNT observations within each trial. Recording duration was included as a log-offset to account for small differences in observation time.

Food selectivity was evaluated separately using the paired numbers of bites on HNT and LNT within active feeding trials. This analysis quantified the relative allocation of bites to the two tile types and tested whether this allocation varied with measured NH3 concentration or experimental time.

The sections below describe data preparation, candidate-model evaluation, model diagnostics, effect-size estimation, and model predictions for both analyses.

```r
library(tidyverse)
library(lubridate)
library(glmmTMB)
library(DHARMa)
library(ggeffects)
library(emmeans)
library(patchwork)

knitr::opts_chunk$set(
  fig.align = "center",
  out.width = "90%"
)
```

2. Data import and preparation

```r
blenny <- read.csv("Blenny_baseline.csv") %>%
  mutate(
    Date = as.Date(Date),
    Setting = factor(Setting, levels = c("S1", "S2", "S3")),
    Treatment = factor(Treatment, levels = c("C", "T")),
    Fish = factor(Fish),
    Bite_LNT = as.integer(Bite_LNT),
    Bite_HNT = as.integer(Bite_HNT),
    Time_recorded = as.numeric(Time_recorded),
    NH3_umol = as.numeric(NH3_umol)
  )
```

2.1 Basic data checks

```r
required_variables <- c(
  "Date", "Setting", "Treatment", "Fish", "Time_recorded",
  "Bite_LNT", "Bite_HNT", "NH3_umol"
)

stopifnot(all(required_variables %in% names(blenny)))
stopifnot(
  all(!is.na(blenny$Date)),
  all(!is.na(blenny$Fish)),
  all(!is.na(blenny$Bite_LNT)),
  all(!is.na(blenny$Bite_HNT)),
  all(!is.na(blenny$Time_recorded)),
  all(!is.na(blenny$NH3_umol))
)
stopifnot(
  all(blenny$Bite_LNT >= 0),
  all(blenny$Bite_HNT >= 0),
  all(blenny$Time_recorded > 0)
)
```

2.2 Trial identifier and experimental time

Each row in the original wide-format dataset represents one fish during one feeding trial. Because every trial contains one HNT and one LNT observation after conversion to long format, a unique Trial ID is created before reshaping the data.

Experimental time is expressed as days since the first feeding observation and centred around its mean.

```r
blenny <- blenny %>%
  mutate(
    TrialID = interaction(Fish, Date, drop = TRUE),
    DaysSinceStart = as.numeric(Date - min(Date)),
    DaysSinceStart_c = DaysSinceStart - mean(DaysSinceStart)
  )

stopifnot(!anyDuplicated(blenny$TrialID))
```

2.3 Tile-level dataset for the primary grazing analysis

Each feeding trial contributes two observations: one LNT and one HNT.

```r
bites_long <- blenny %>%
  select(
    TrialID, Fish, Date, DaysSinceStart, DaysSinceStart_c,
    Setting, Treatment, NH3_umol, Time_recorded,
    Bite_LNT, Bite_HNT
  ) %>%
  pivot_longer(
    cols = c(Bite_LNT, Bite_HNT),
    names_to = "TileType",
    values_to = "BiteCount"
```

```r
  ) %>%
  mutate(
    TileType = recode(TileType, Bite_LNT = "LNT", Bite_HNT = "HNT"),
    TileType = factor(TileType, levels = c("LNT", "HNT")),
    BiteCount = as.integer(BiteCount),
    BiteRate = BiteCount / Time_recorded
  )

stopifnot(
  nrow(bites_long) == 2 * nrow(blenny),
  all(table(bites_long$TrialID) == 2)
)

nrow(bites_long)
```

```
## [1] 374
```

```r
length(unique(bites_long$TrialID))
```

```
## [1] 187
```

```r
length(unique(bites_long$Fish))
```

```
## [1] 21
```

2.4 Trial-level dataset for the complementary selectivity analysis

Trials with zero bites on both tiles contain no information about relative bite allocation and are therefore excluded **only** from the selectivity analysis.

```r
selectivity_trial <- blenny %>%
  transmute(
    TrialID, Fish, Date, DaysSinceStart, DaysSinceStart_c,
    Setting, Treatment, NH3_umol, Time_recorded,
    Bites_LNT = Bite_LNT,
    Bites_HNT = Bite_HNT,
    TotalBites = Bite_LNT + Bite_HNT,
    Prop_HNT = if_else(TotalBites > 0, Bites_HNT / TotalBites, NA_real_)
  ) %>%
  filter(TotalBites > 0)

nrow(selectivity_trial)
```

```
## [1] 150
```

```r
zero_bite_trials <- sum(blenny$Bite_LNT + blenny$Bite_HNT == 0)
zero_bite_trials
```

```
## [1] 37
```

3. Tile-level analysis of grazing activity

The response variable was the number of bites recorded on each tile. Tile type, measured NH3 concentration, and centred experimental time were included as fixed effects. Fish identity was included as a random intercept to account for repeated observations of the same individuals, and Trial ID was nested within Fish ID to account for the paired HNT and LNT observations obtained during the same feeding event and additional trial-to-trial variation within individuals `(1 | Fish/TrialID)`. Recording duration was included as a log-transformed offset.

3.1 Candidate model set

Candidate models were fitted using identical fixed- and random-effects structures and differed only in their assumed response distribution and zero-inflation structure.

```
grazing_formula <- BiteCount ~
  TileType +
  NH3_umol +
  DaysSinceStart_c +
  (1 | Fish/TrialID) +
  offset(log(Time_recorded))

m_grazing_pois <- glmmTMB(
  formula = grazing_formula,
  data = bites_long,
  family = poisson(link = "log")
)

m_grazing_nb1 <- glmmTMB(
  formula = grazing_formula,
  data = bites_long,
  family = nbinom1(link = "log")
)

m_grazing_nb2 <- glmmTMB(
  formula = grazing_formula,
  data = bites_long,
  family = nbinom2(link = "log")
)

m_grazing_nb1_zi <- glmmTMB(
  formula = grazing_formula,
  ziformula = ~1,
  data = bites_long,
  family = nbinom1(link = "log")
)

m_grazing_nb2_zi <- glmmTMB(
  formula = grazing_formula,
  ziformula = ~1,
  data = bites_long,
  family = nbinom2(link = "log")
)
```

3.2 Model selection

Model-family selection was based on both AIC and residual diagnostics. The negative-binomial type 1 (NB1) model was selected because it had the lowest AIC among models with reliable convergence and showed satisfactory simulation-based residual diagnostics. The zero-inflated negative-binomial models produced non-positive-definite Hessian matrices, indicating unreliable parameter estimation, and were therefore excluded from further consideration.

3.2.1 AIC

```
grazing_aic <- AIC(
  m_grazing_pois,
  m_grazing_nb1,
  m_grazing_nb2,
  m_grazing_nb1_zi,
  m_grazing_nb2_zi
) %>%
  as.data.frame() %>%
  arrange(AIC) %>%
  mutate(
    deltaAIC = AIC - min(AIC, na.rm = TRUE)
  )

grazing_aic
```

```
##                  df      AIC   deltaAIC
## m_grazing_nb1     7 3205.659    0.00000
## m_grazing_nb2     7 3272.549   66.88997
## m_grazing_pois    6 7054.336 3848.67706
## m_grazing_nb1_zi  8       NA         NA
## m_grazing_nb2_zi  8       NA         NA
```

3.2.2. DHARMa diagnostics

```
set.seed(123)

res_grazing_pois <- simulateResiduals(m_grazing_pois, n = 1000, plot = FALSE)
res_grazing_nb1 <- simulateResiduals(m_grazing_nb1, n = 1000, plot = FALSE)
res_grazing_nb2 <- simulateResiduals(m_grazing_nb2, n = 1000, plot = FALSE)
res_grazing_nb1_zi <- simulateResiduals(m_grazing_nb1_zi, n = 1000, plot = FALSE)
res_grazing_nb2_zi <- simulateResiduals(m_grazing_nb2_zi, n = 1000, plot = FALSE)
```

```
plot(res_grazing_nb1)
```

NB1 diagnostics

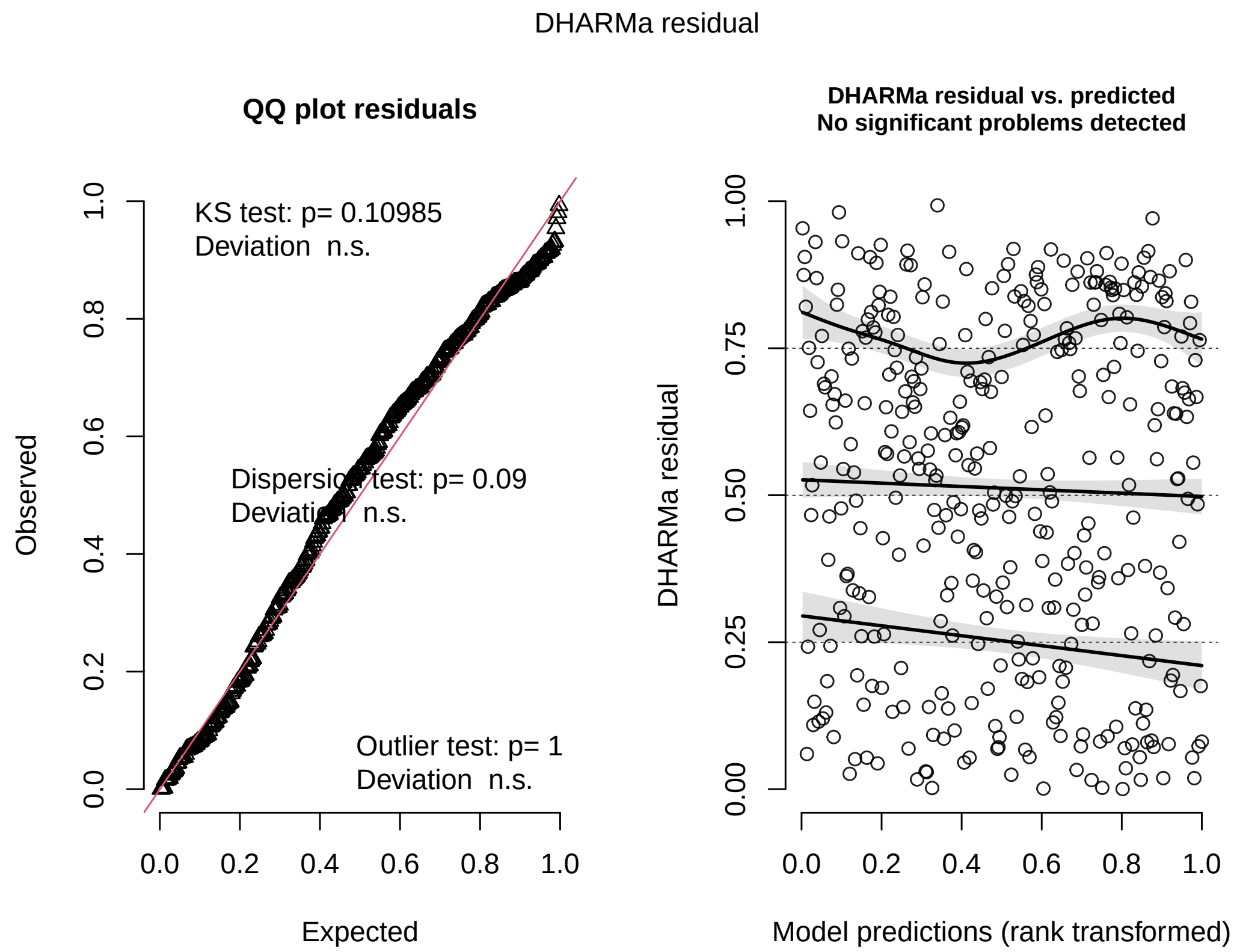


```
testUniformity(res_grazing_nb1)
```

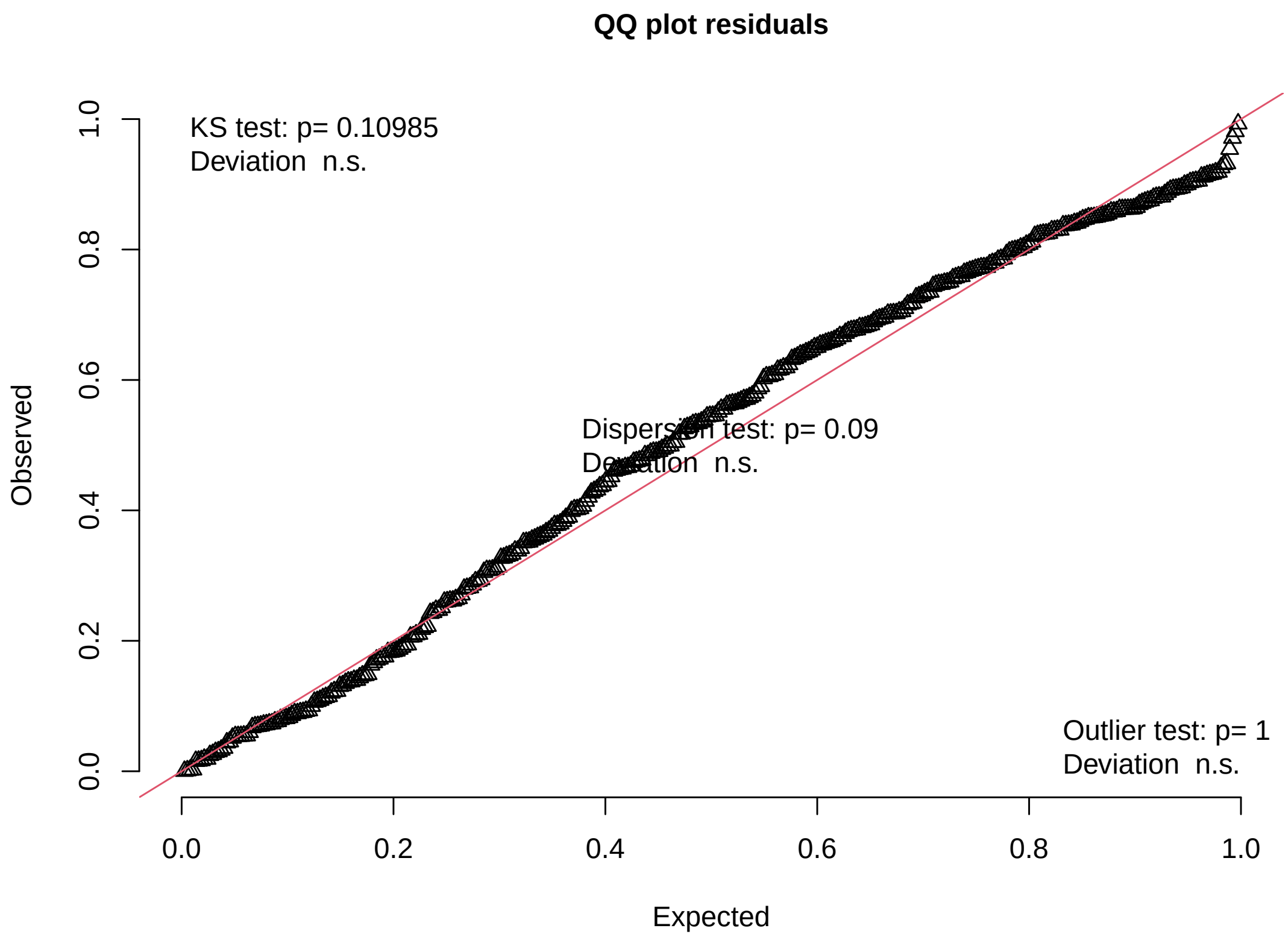


```
##
##  Asymptotic one-sample Kolmogorov-Smirnov test
##
## data:  simulationOutput$scaledResiduals
## D = 0.062283, p-value = 0.1098
## alternative hypothesis: two-sided
```

```
testDispersion(res_grazing_nb1)
```

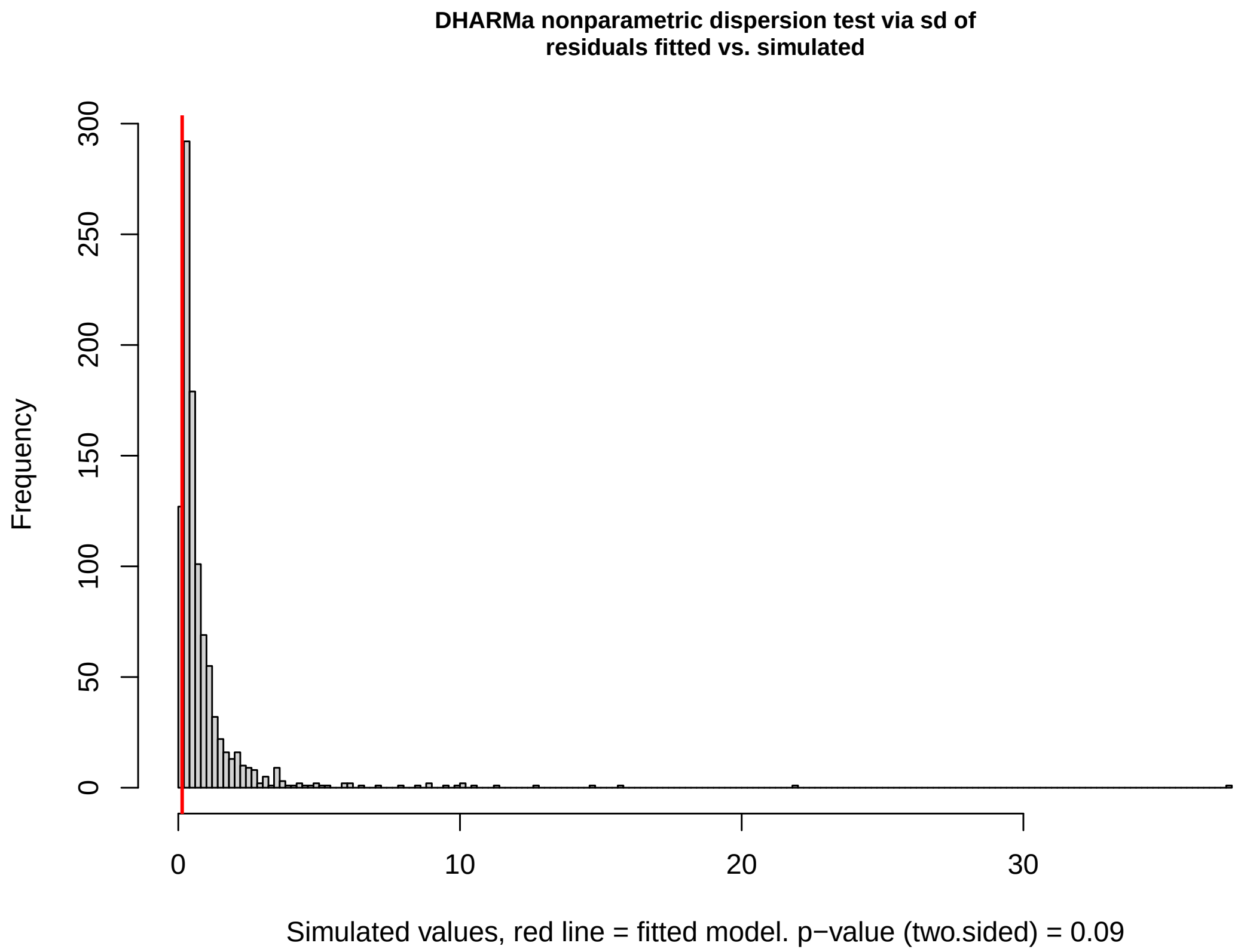


```
##
##  DHARMa nonparametric dispersion test via sd of residuals fitted vs.
##  simulated
##
## data:  simulationOutput
## dispersion = 0.14337, p-value = 0.09
## alternative hypothesis: two.sided
```

```r
testZeroInflation(res_grazing_nb1)
```

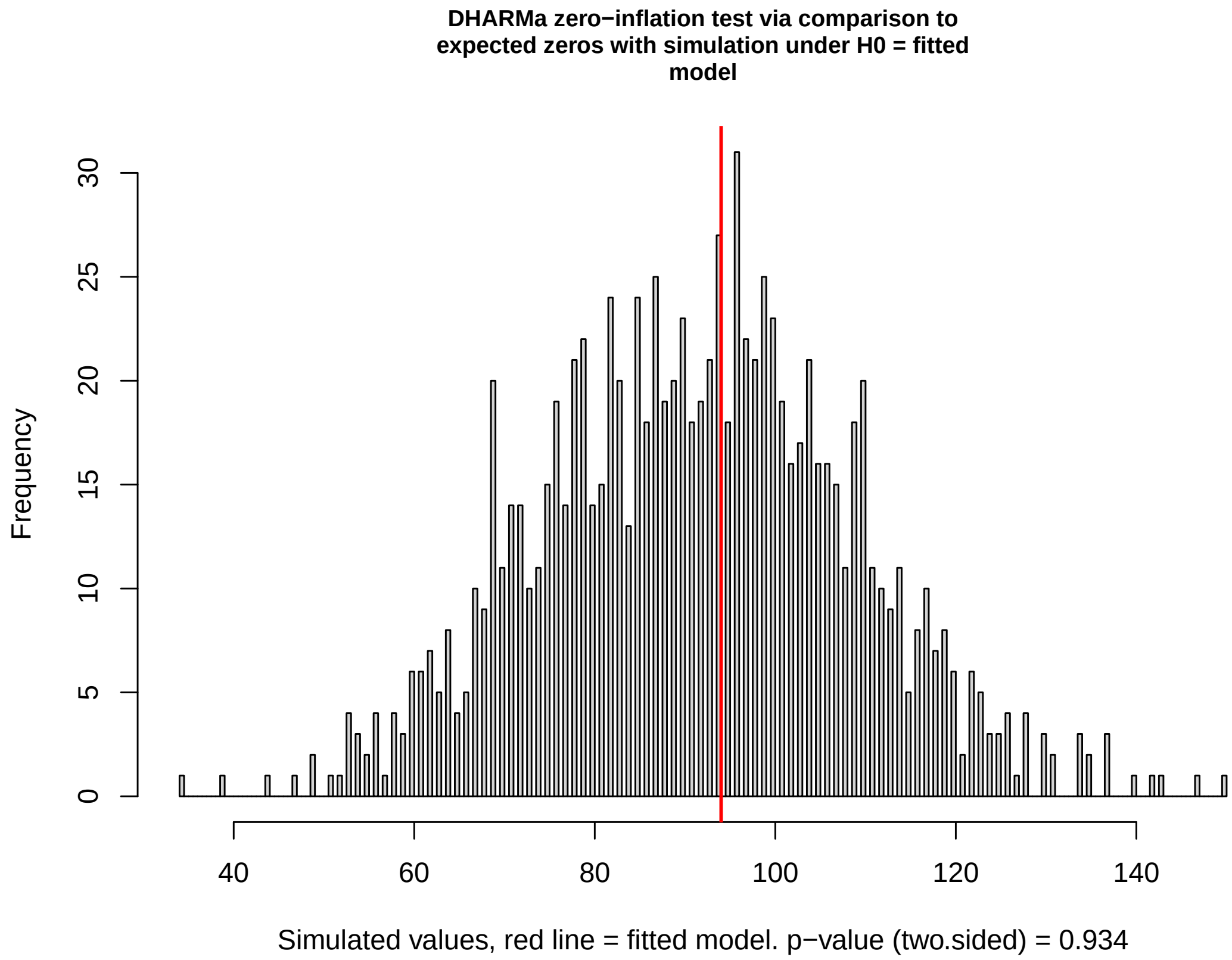


```
##
##  DHARMa zero-inflation test via comparison to expected zeros with
##  simulation under H0 = fitted model
##
## data:  simulationOutput
## ratioObsSim = 1.0271, p-value = 0.934
## alternative hypothesis: two.sided
```

```
testOutliers(res_grazing_nb1)
```

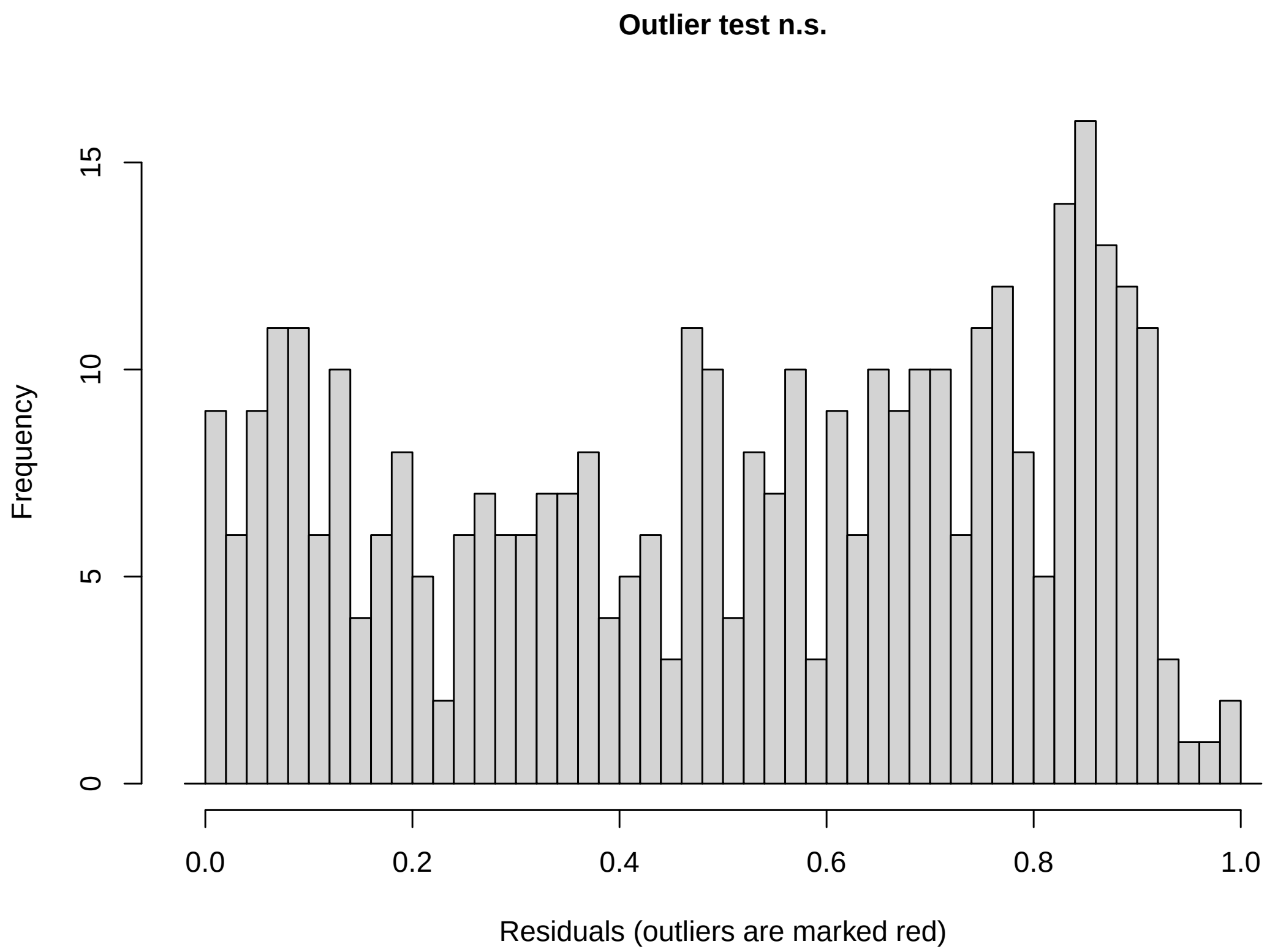


```
##
##  DHARMa bootstrapped outlier test
##
## data:  res_grazing_nb1
## outliers at both margin(s) = 0, observations = 374, p-value = 1
## alternative hypothesis: two.sided
##  percent confidence interval:
##  0.000000000 0.005347594
## sample estimates:
## outlier frequency (expected: 0.00117647058823529 )
##                                                   0
```

```
plot(res_grazing_pois)
```

Poisson diagnostics

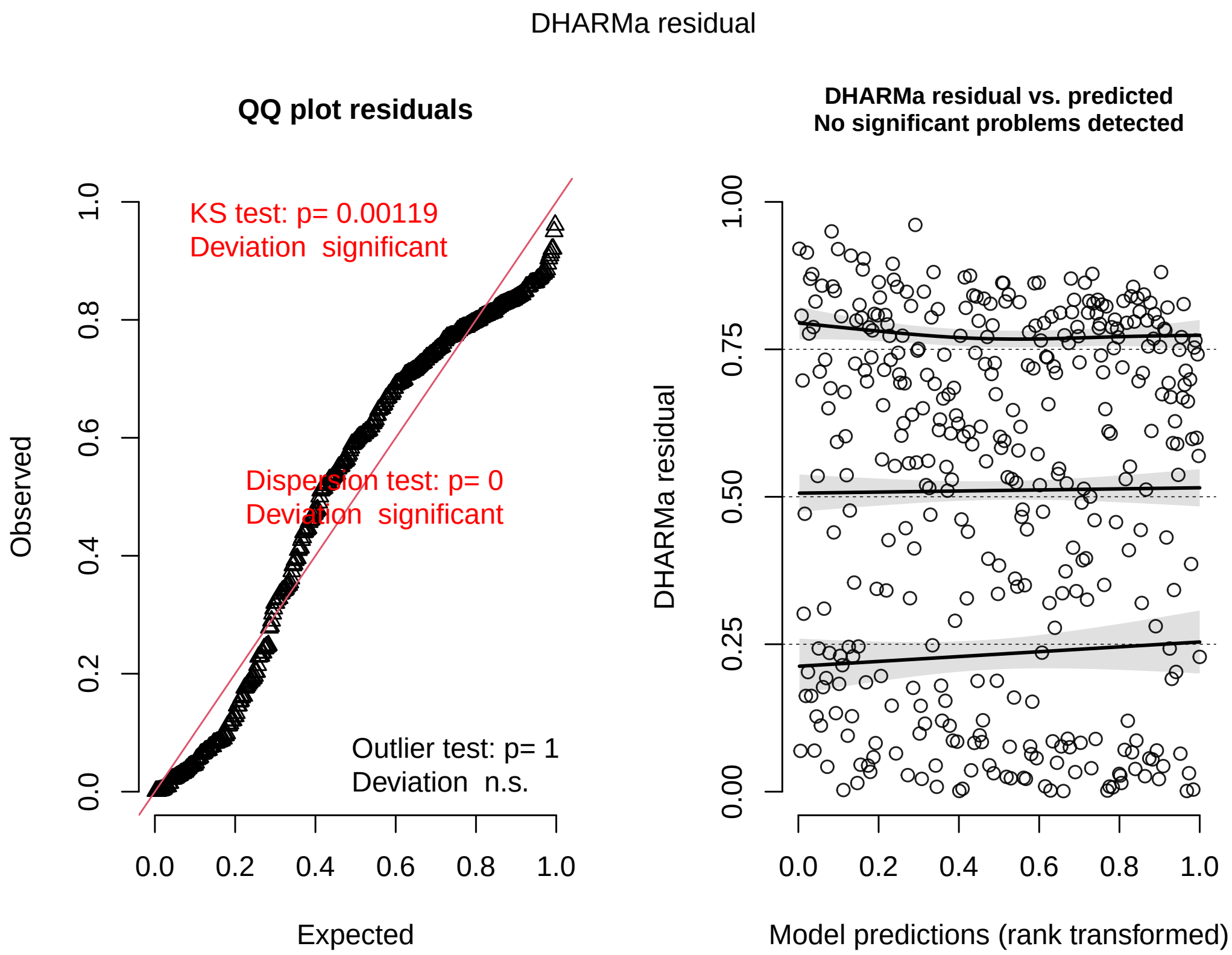


```
testUniformity(res_grazing_pois)
```

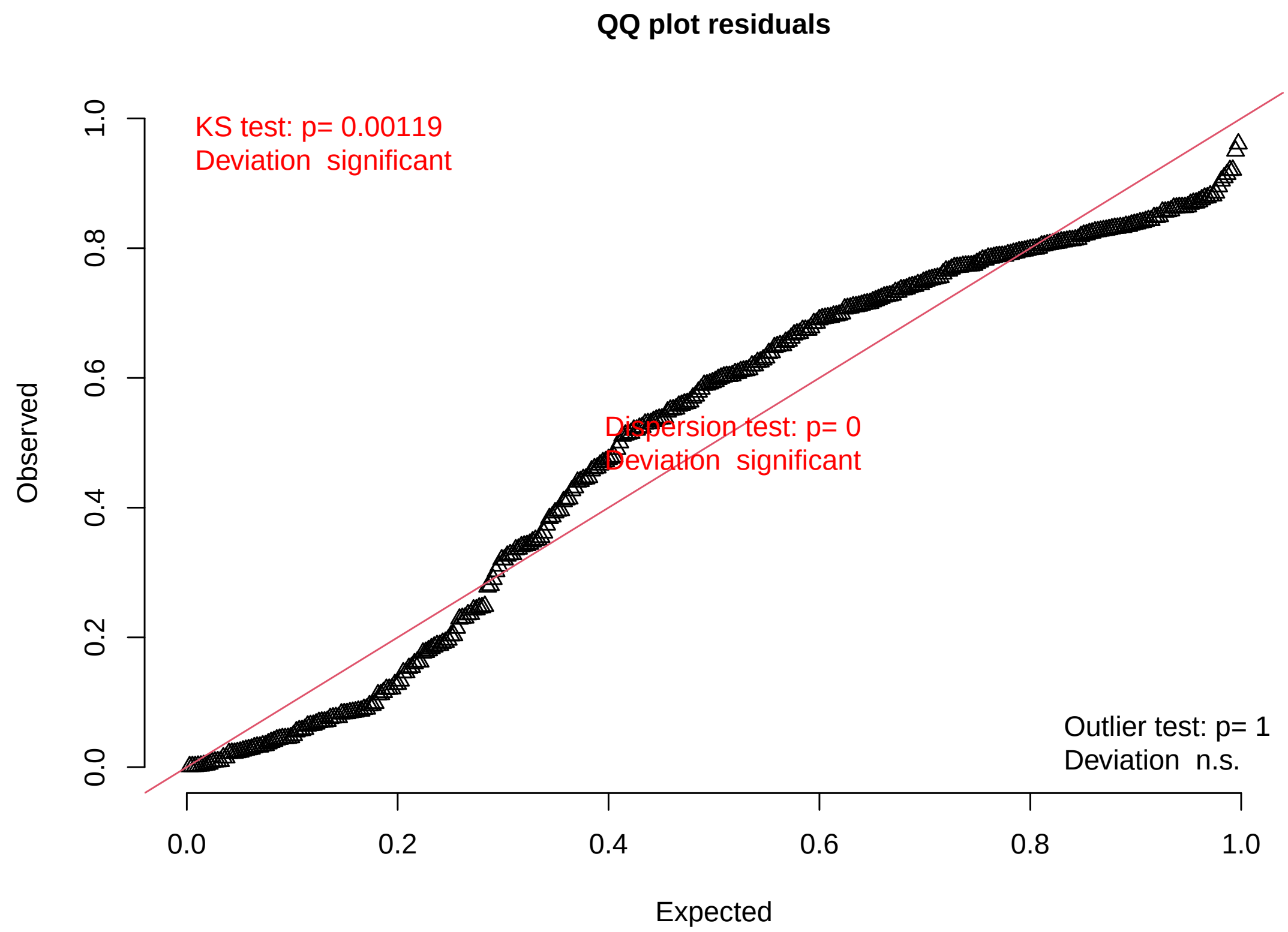


```
##
##  Asymptotic one-sample Kolmogorov-Smirnov test
##
## data:  simulationOutput$scaledResiduals
## D = 0.09965, p-value = 0.001189
## alternative hypothesis: two-sided
```

```
testDispersion(res_grazing_pois)
```

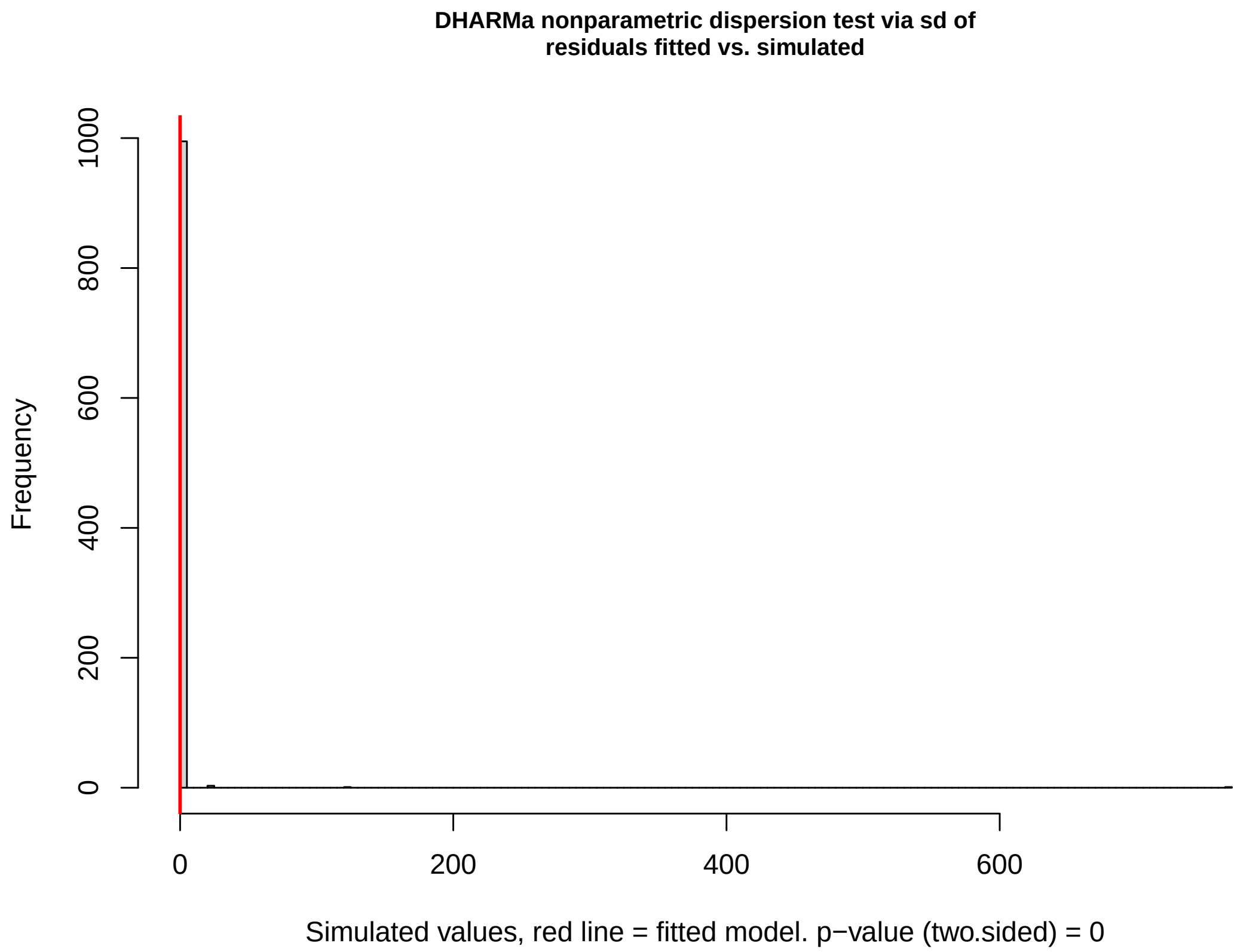


```
##
##  DHARMa nonparametric dispersion test via sd of residuals fitted vs.
##  simulated
##
## data:  simulationOutput
## dispersion = 1.9291e-05, p-value < 2.2e-16
## alternative hypothesis: two.sided
```

```
testZeroInflation(res_grazing_pois)
```

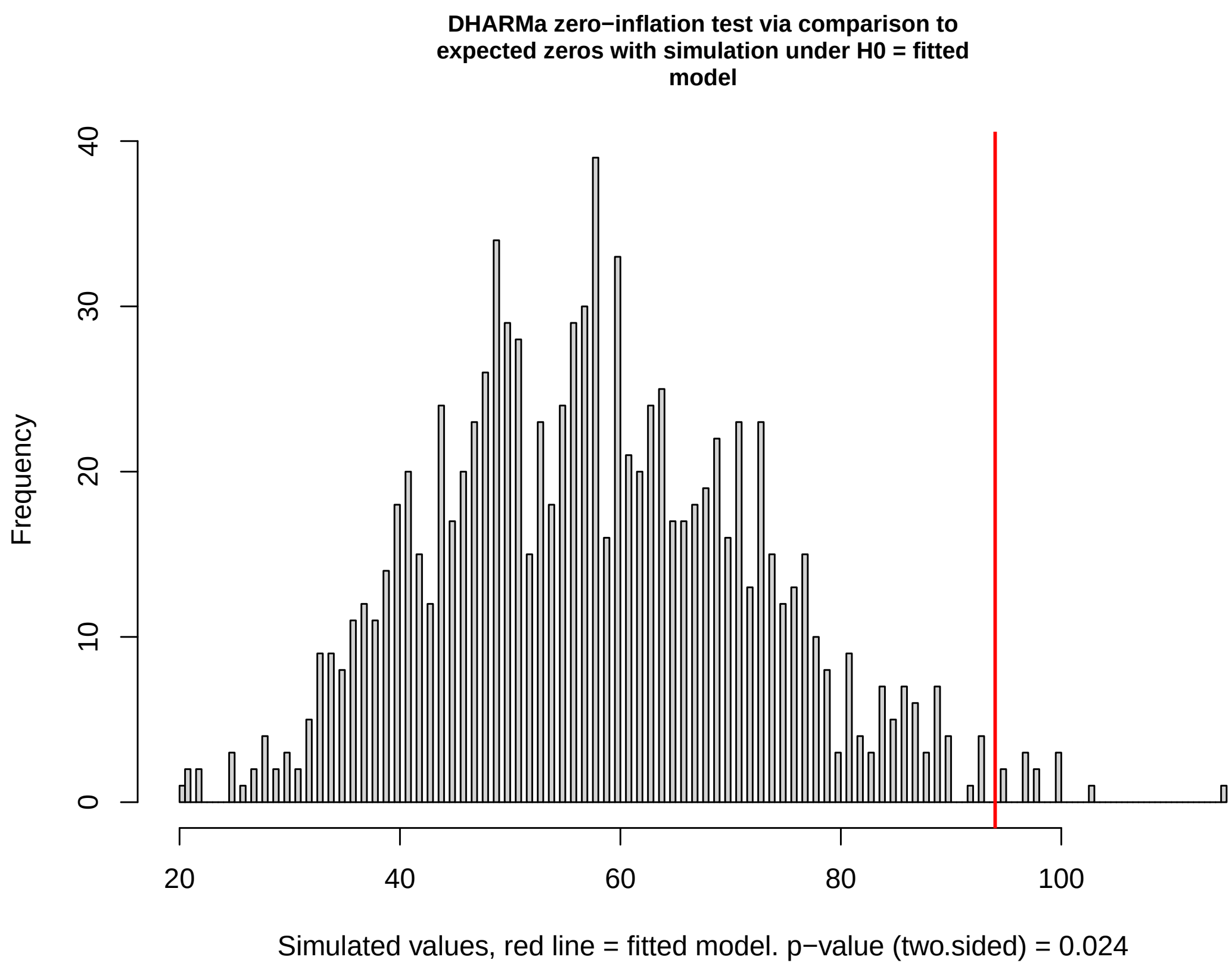


```
##
##  DHARMa zero-inflation test via comparison to expected zeros with
##  simulation under H0 = fitted model
##
## data:  simulationOutput
## ratioObsSim = 1.6224, p-value = 0.024
## alternative hypothesis: two.sided
```

```
testOutliers(res_grazing_pois)
```

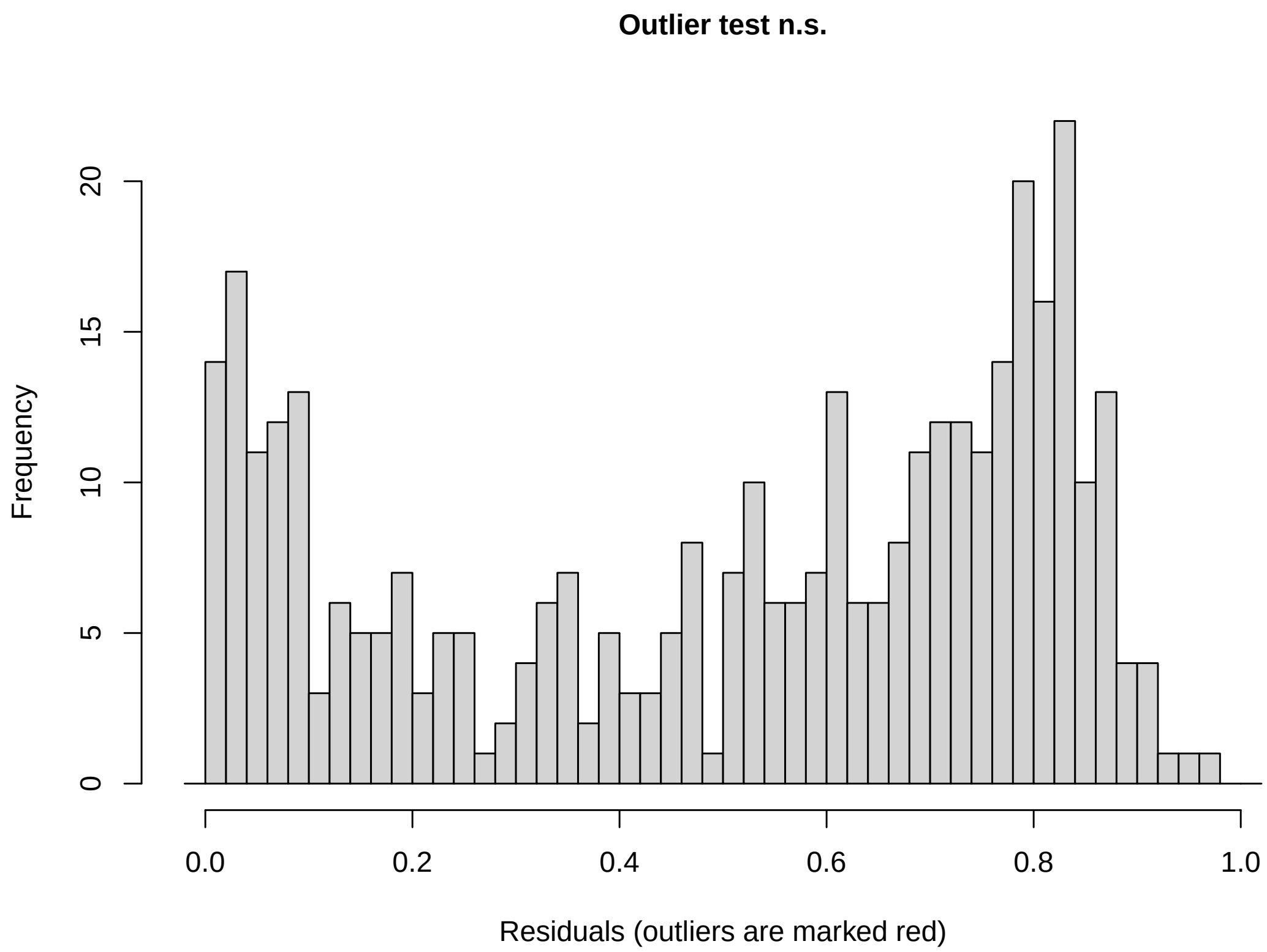


```
##
##  DHARMa bootstrapped outlier test
##
## data:  res_grazing_pois
## outliers at both margin(s) = 0, observations = 374, p-value = 1
## alternative hypothesis: two.sided
##  percent confidence interval:
##  0.00000000 0.01069519
## sample estimates:
## outlier frequency (expected: 0.00160427807486631 )
##                                                  0
```

```
plot(res_grazing_nb2)
```

NB2 diagnostics

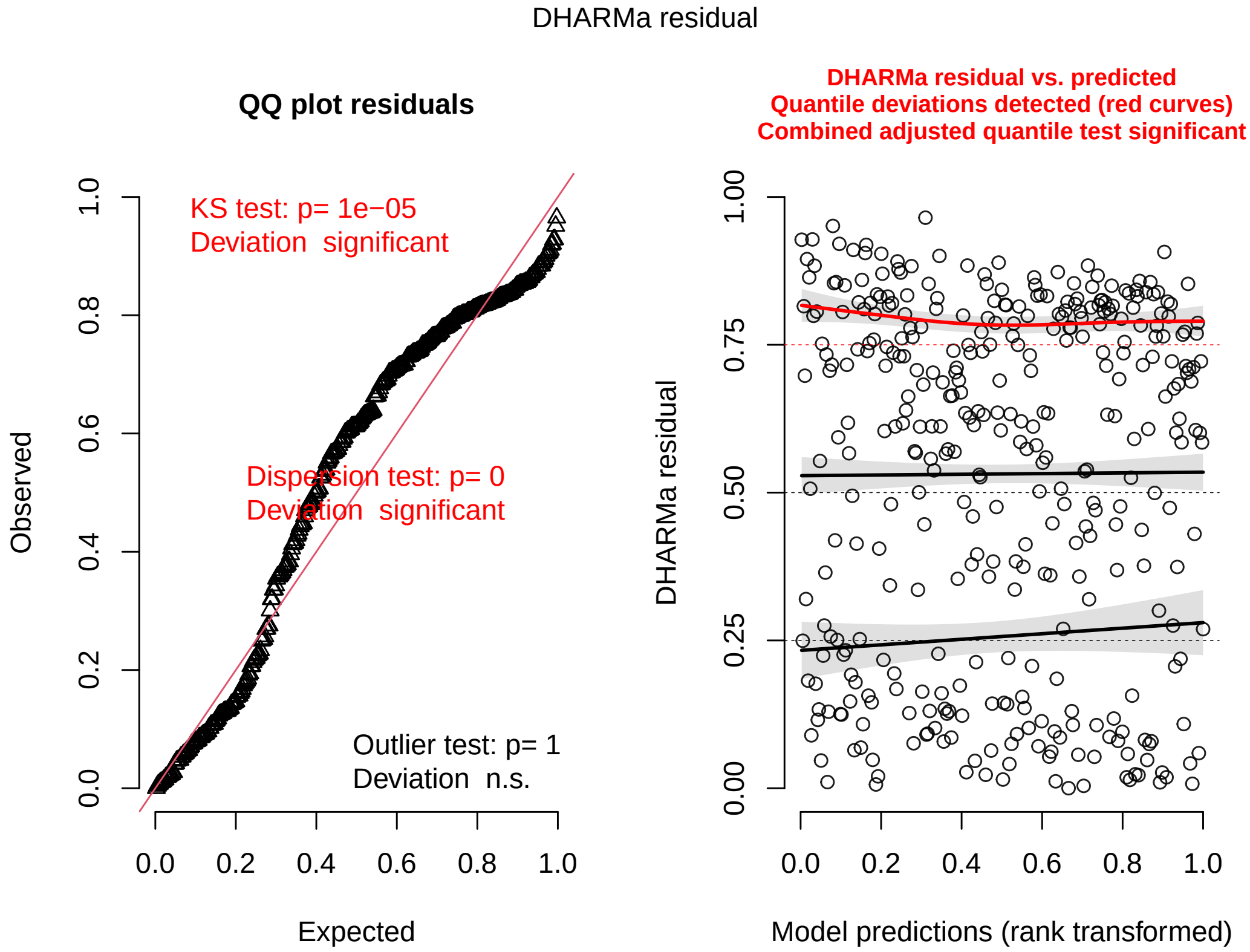


```
testUniformity(res_grazing_nb2)
```

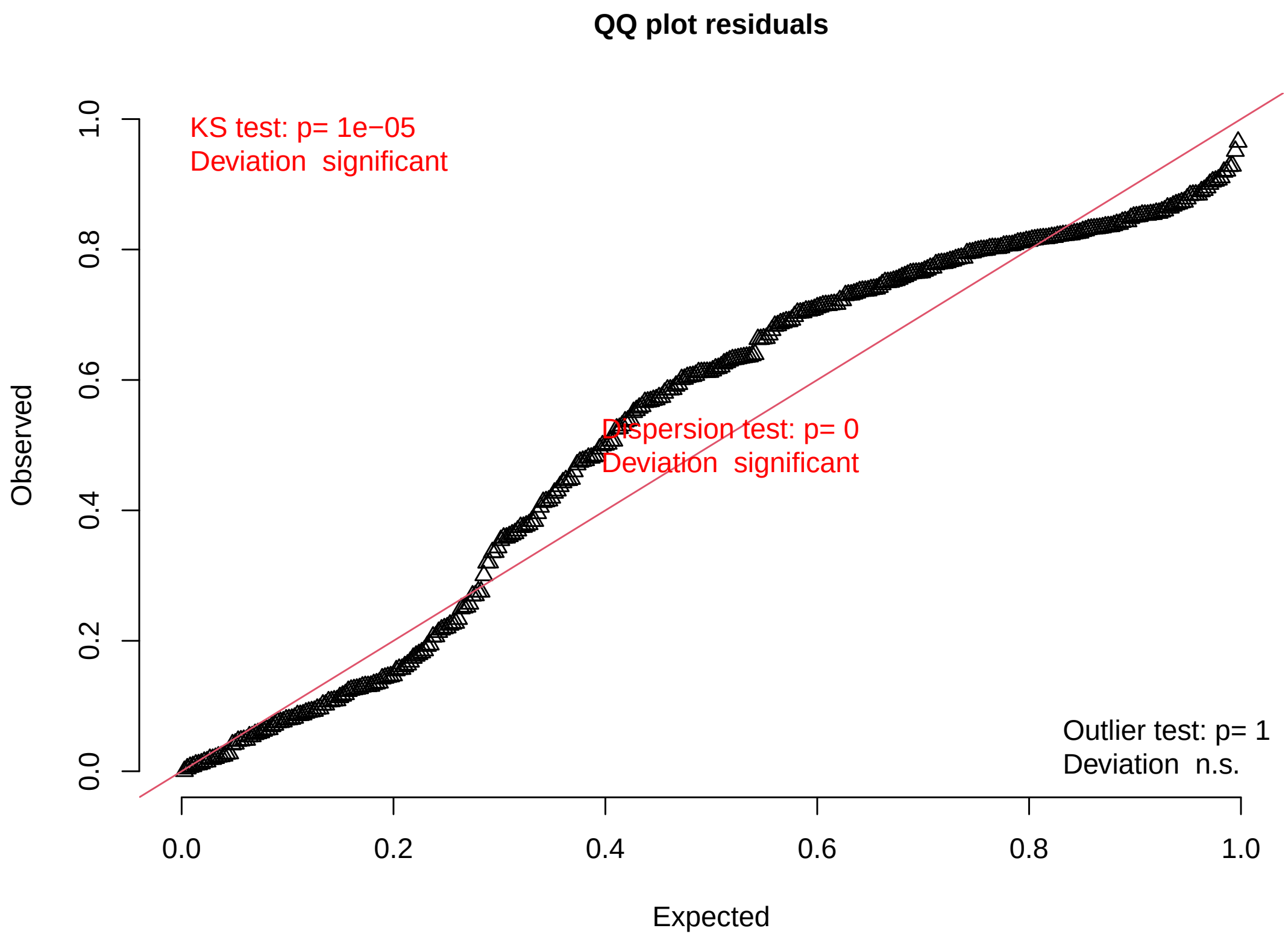


```
##
##  Asymptotic one-sample Kolmogorov-Smirnov test
##
## data:  simulationOutput$scaledResiduals
## D = 0.13041, p-value = 5.973e-06
## alternative hypothesis: two-sided
```

```
testDispersion(res_grazing_nb2)
```

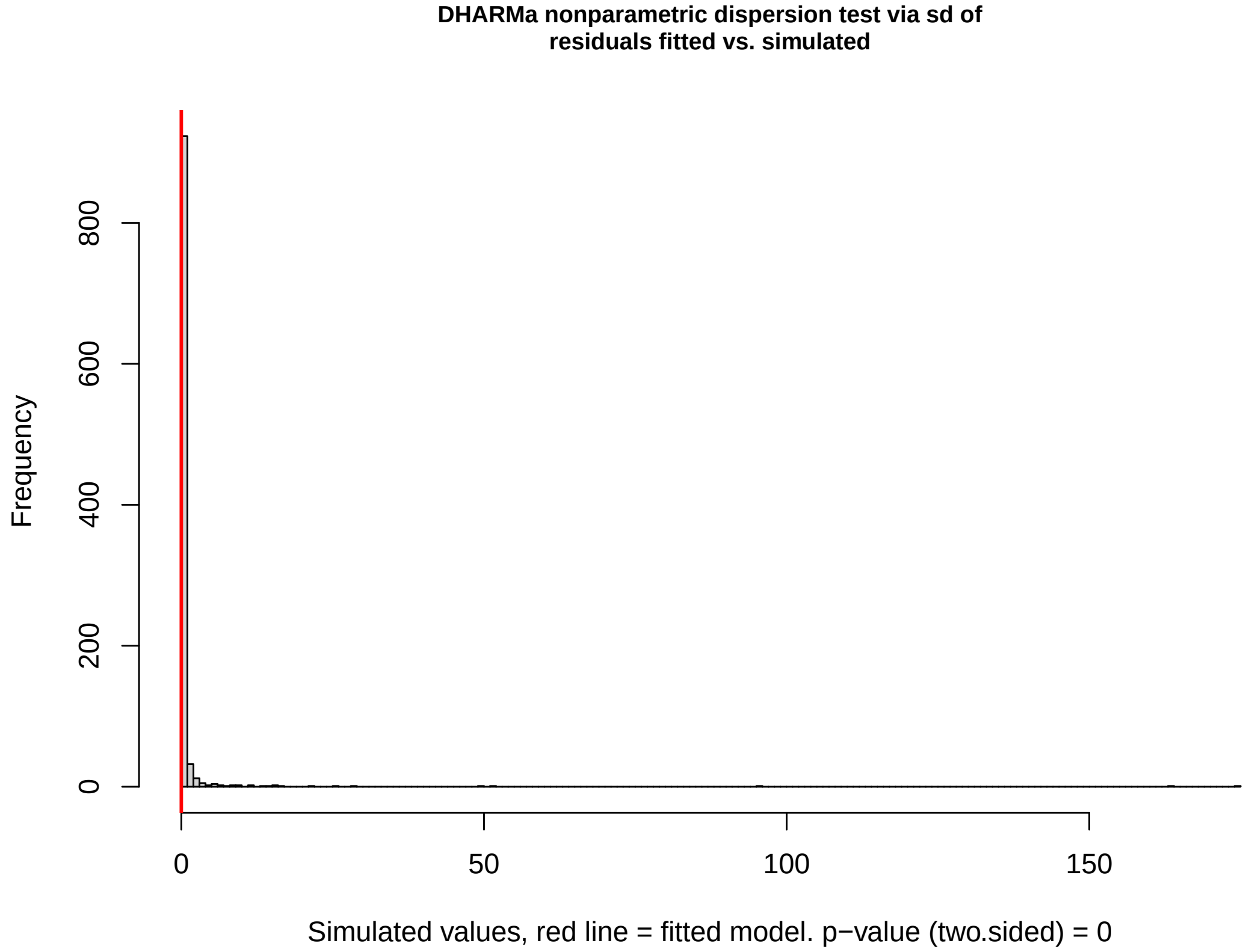


```
##
##  DHARMa nonparametric dispersion test via sd of residuals fitted vs.
##  simulated
##
## data:  simulationOutput
## dispersion = 0.00031766, p-value < 2.2e-16
## alternative hypothesis: two.sided
```

```
testZeroInflation(res_grazing_nb2)
```

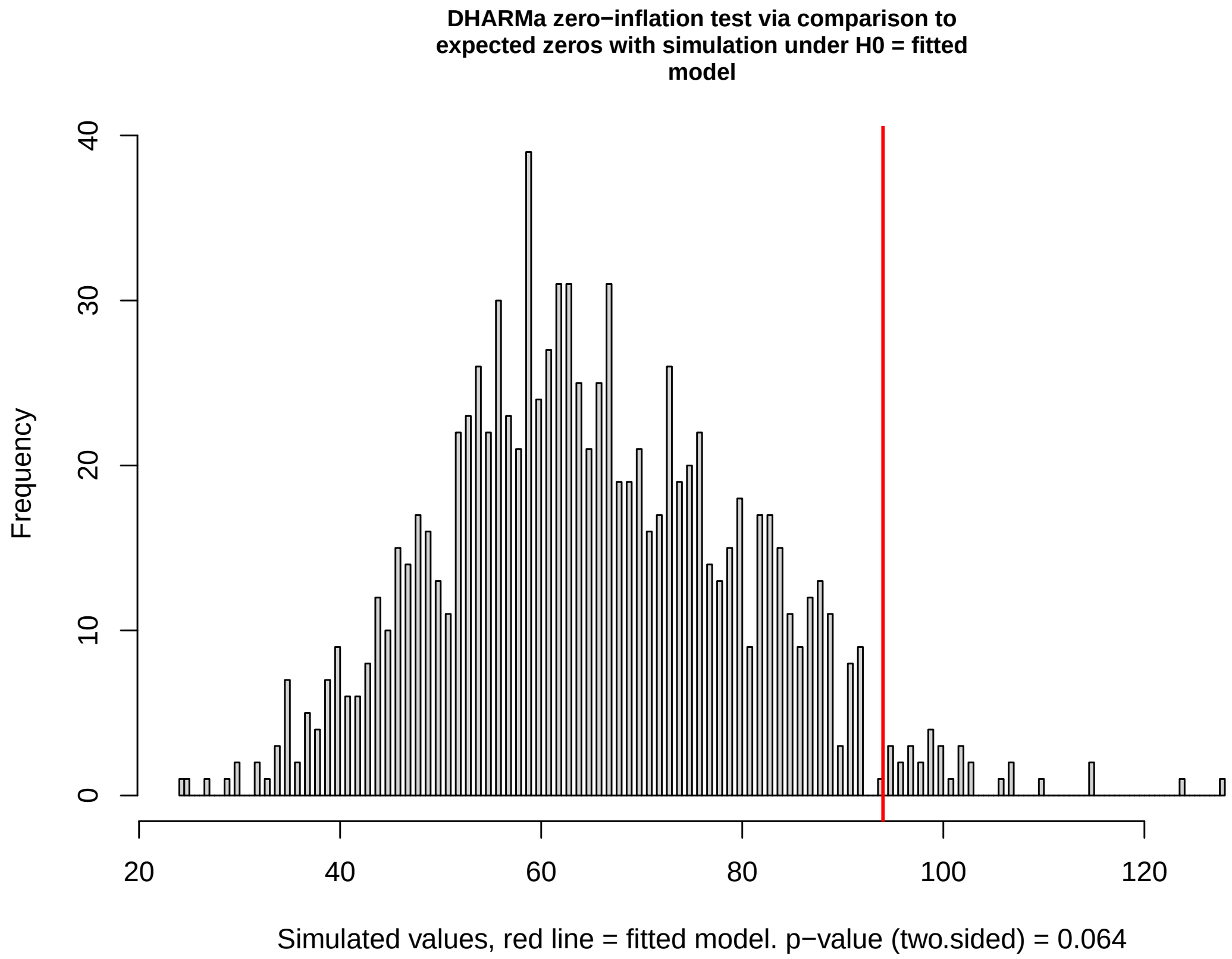


```
##
##  DHARMa zero-inflation test via comparison to expected zeros with
##  simulation under H0 = fitted model
##
## data:  simulationOutput
## ratioObsSim = 1.4423, p-value = 0.064
## alternative hypothesis: two.sided
```

```
testOutliers(res_grazing_nb2)
```

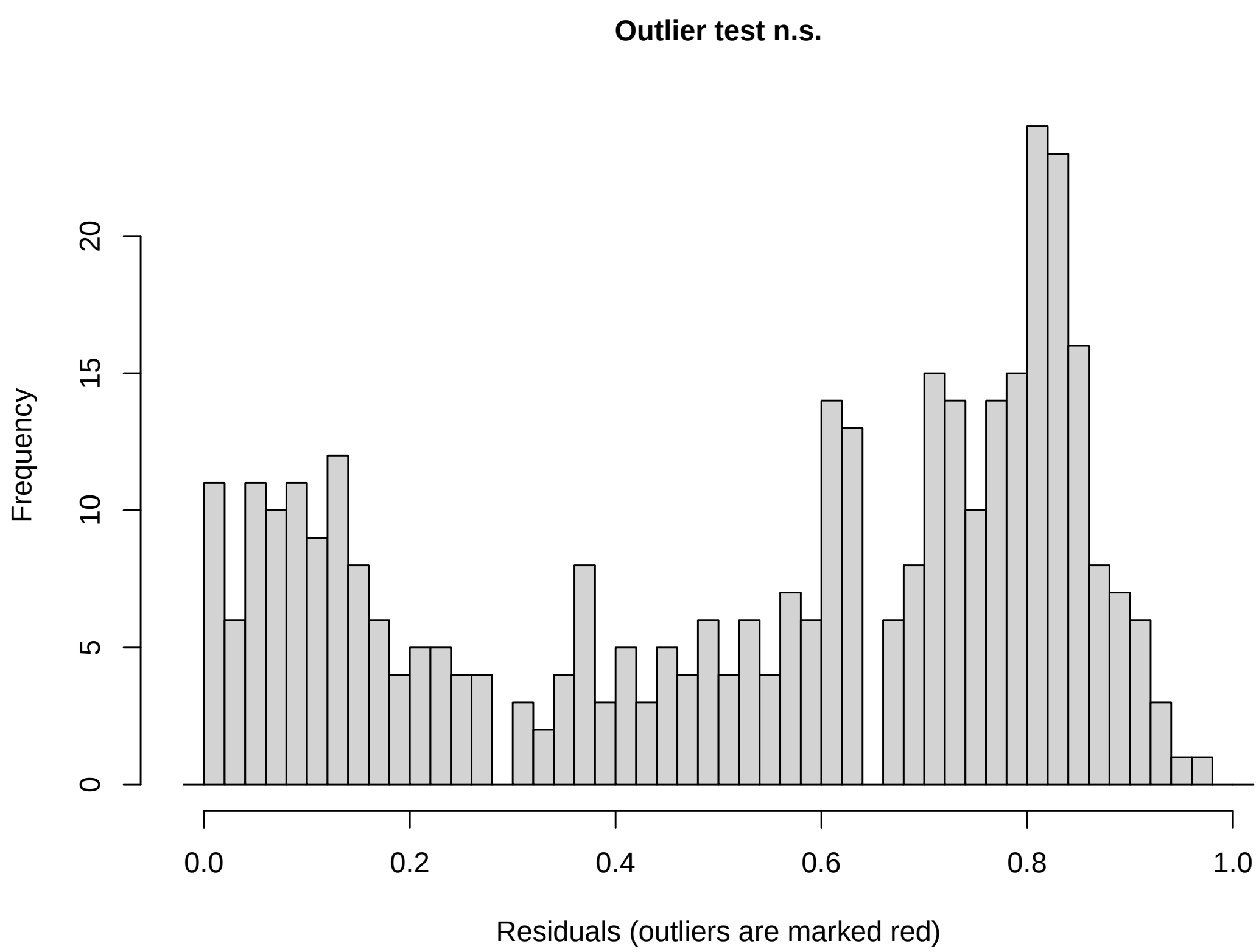


```
##
##  DHARMa bootstrapped outlier test
##
## data:  res_grazing_nb2
## outliers at both margin(s) = 0, observations = 374, p-value = 1
## alternative hypothesis: two.sided
##  percent confidence interval:
##  0.00000000 0.00802139
## sample estimates:
## outlier frequency (expected: 0.00133689839572193 )
##                                                  0
```

```
plot(res_grazing_nb1_zi)
```

Zero-inflated NB1 diagnostics

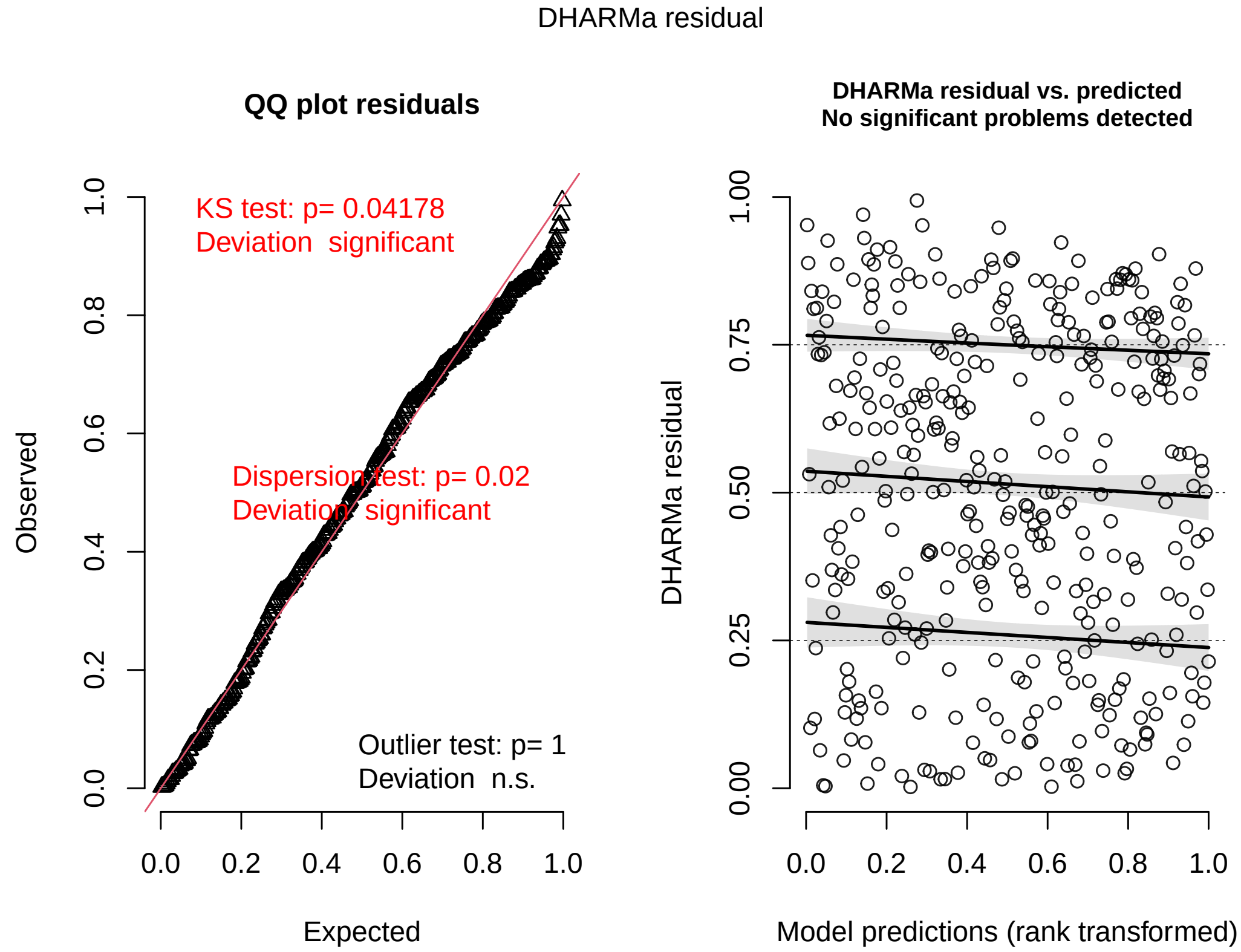


```
testUniformity(res_grazing_nb1_zi)
```

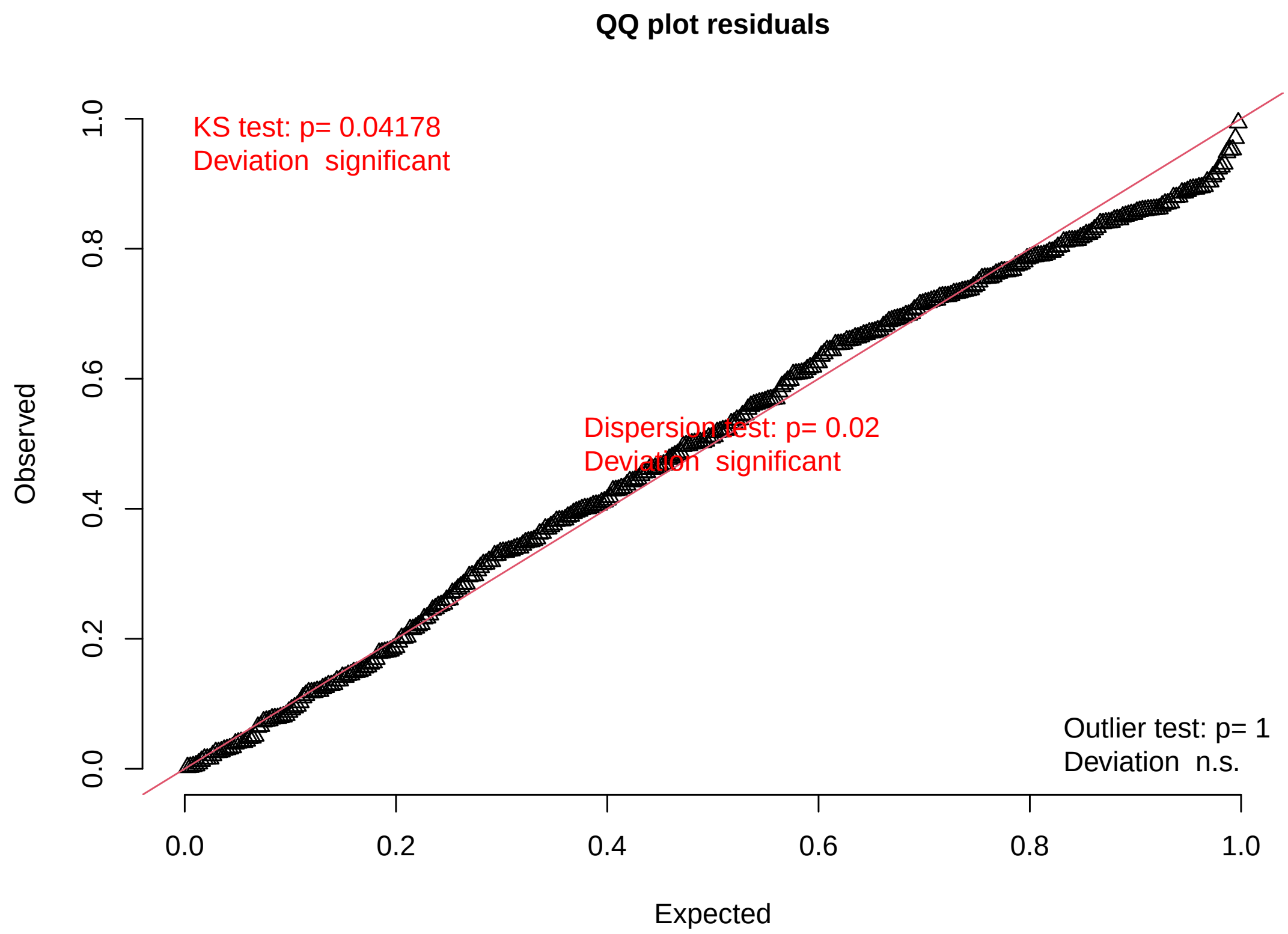


```
##
##  Asymptotic one-sample Kolmogorov-Smirnov test
##
## data:  simulationOutput$scaledResiduals
## D = 0.071914, p-value = 0.04178
## alternative hypothesis: two-sided
```

```
testDispersion(res_grazing_nb1_zi)
```

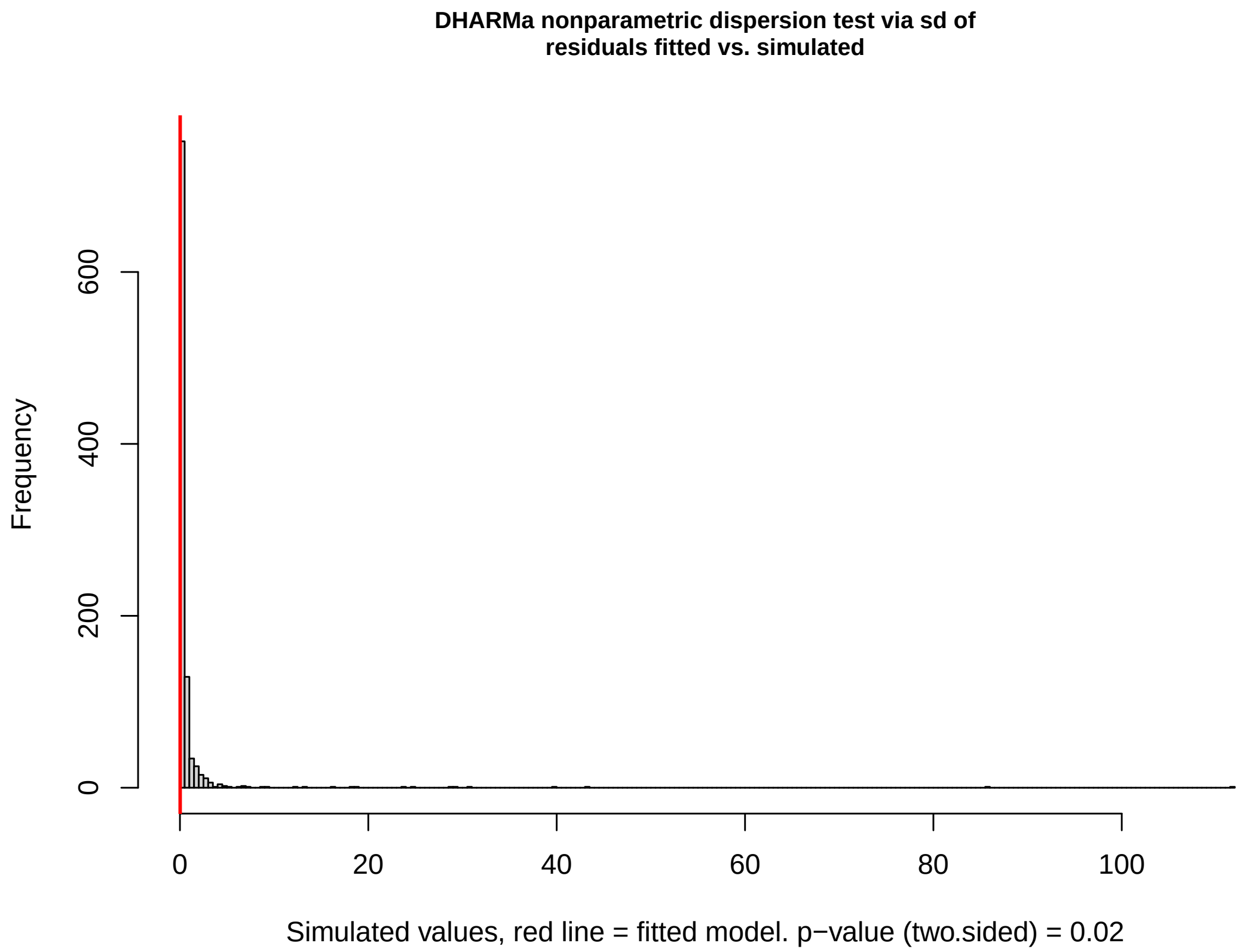


```
##
##  DHARMa nonparametric dispersion test via sd of residuals fitted vs.
##  simulated
##
## data:  simulationOutput
## dispersion = 0.026361, p-value = 0.02
## alternative hypothesis: two.sided
```

```
testZeroInflation(res_grazing_nb1_zi)
```

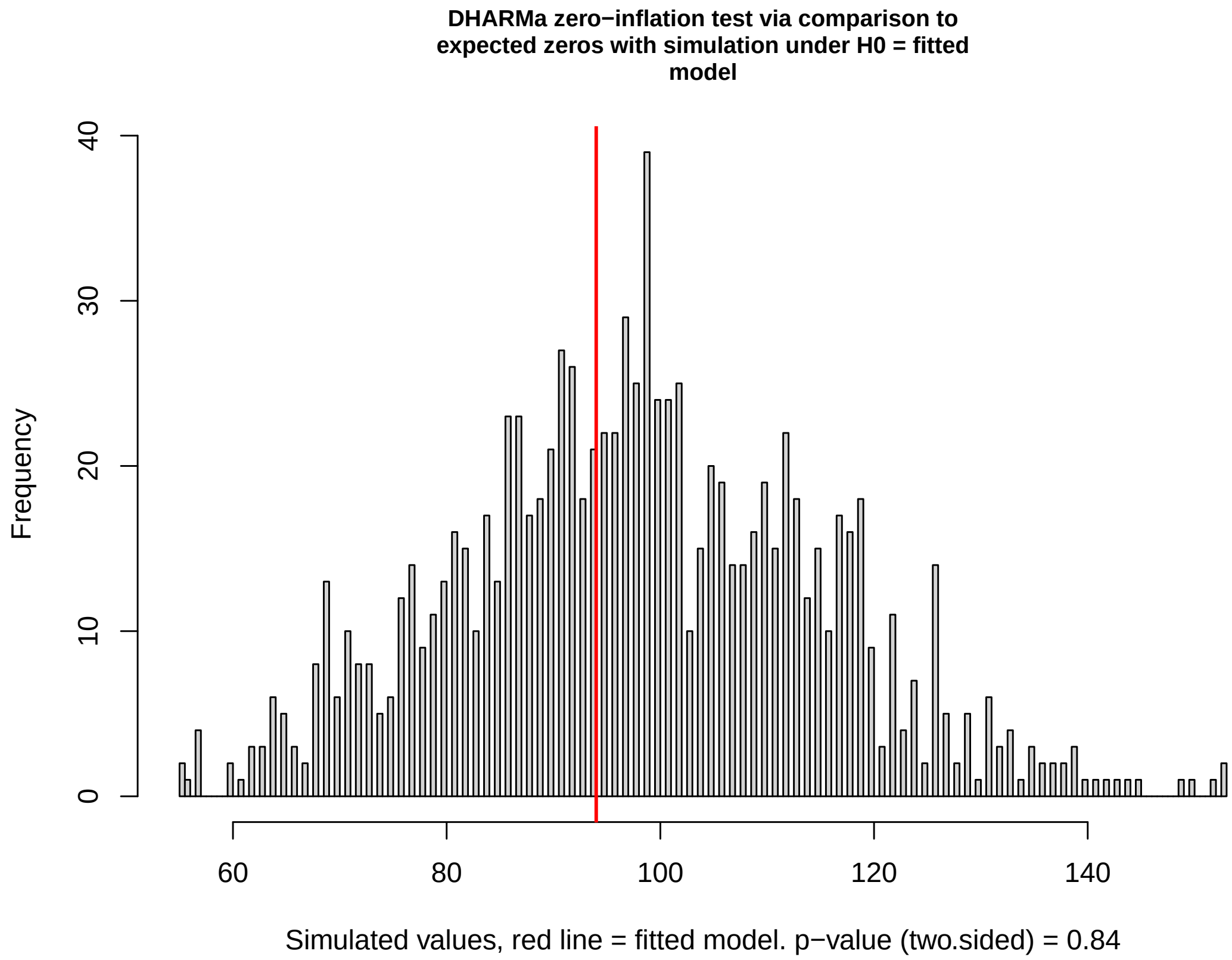


```
##
##  DHARMa zero-inflation test via comparison to expected zeros with
##  simulation under H0 = fitted model
##
## data:  simulationOutput
## ratioObsSim = 0.95813, p-value = 0.84
## alternative hypothesis: two.sided
```

```
testOutliers(res_grazing_nb1_zi)
```

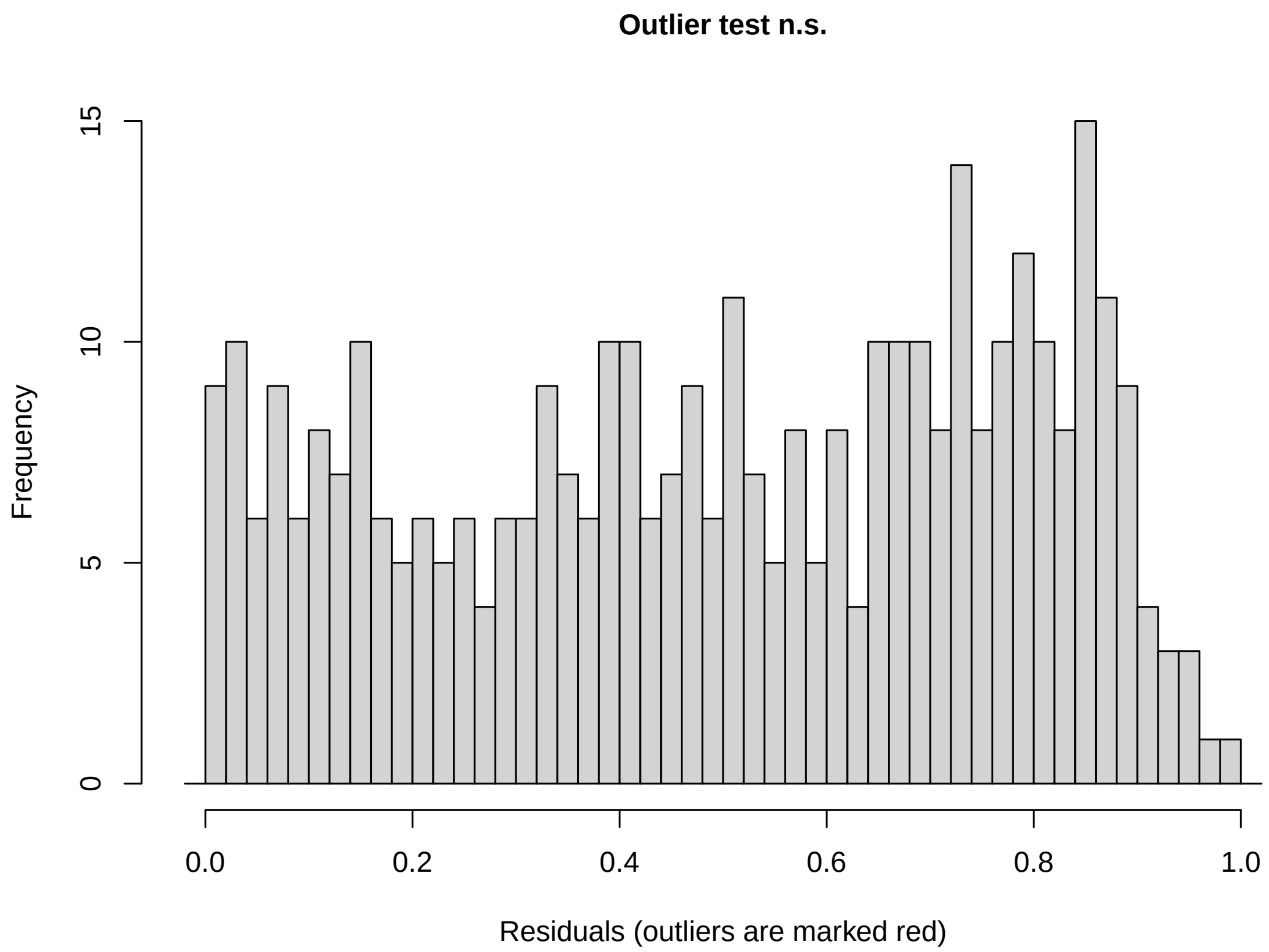


```
##
##  DHARMa bootstrapped outlier test
##
## data:  res_grazing_nb1_zi
## outliers at both margin(s) = 0, observations = 374, p-value = 1
## alternative hypothesis: two.sided
##  percent confidence interval:
##  0.000000000 0.005347594
## sample estimates:
## outlier frequency (expected: 0.00101604278074866 )
##                                                   0
```

```
plot(res_grazing_nb2_zi)
```

Zero-inflated NB2 diagnostics

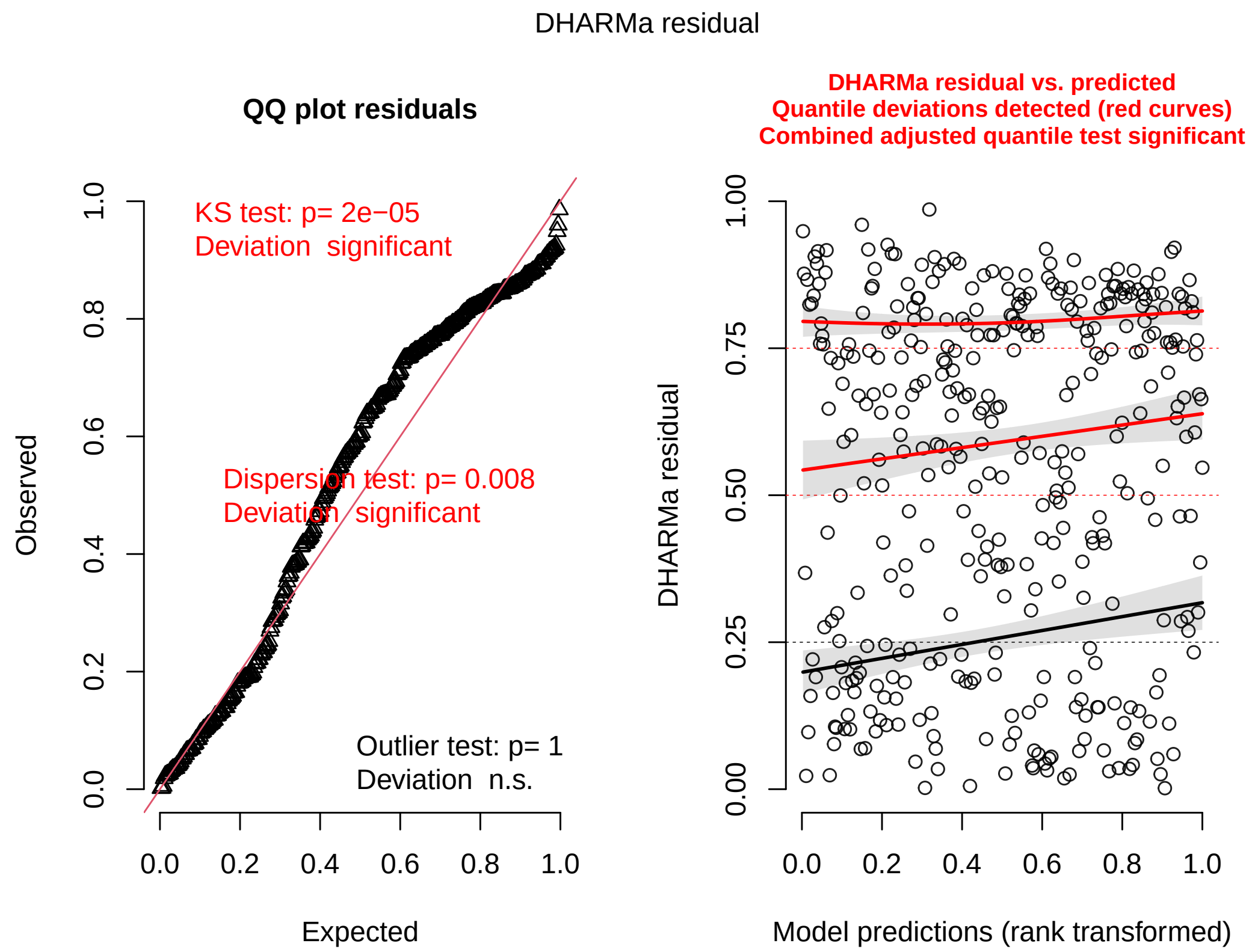


```
testUniformity(res_grazing_nb2_zi)
```

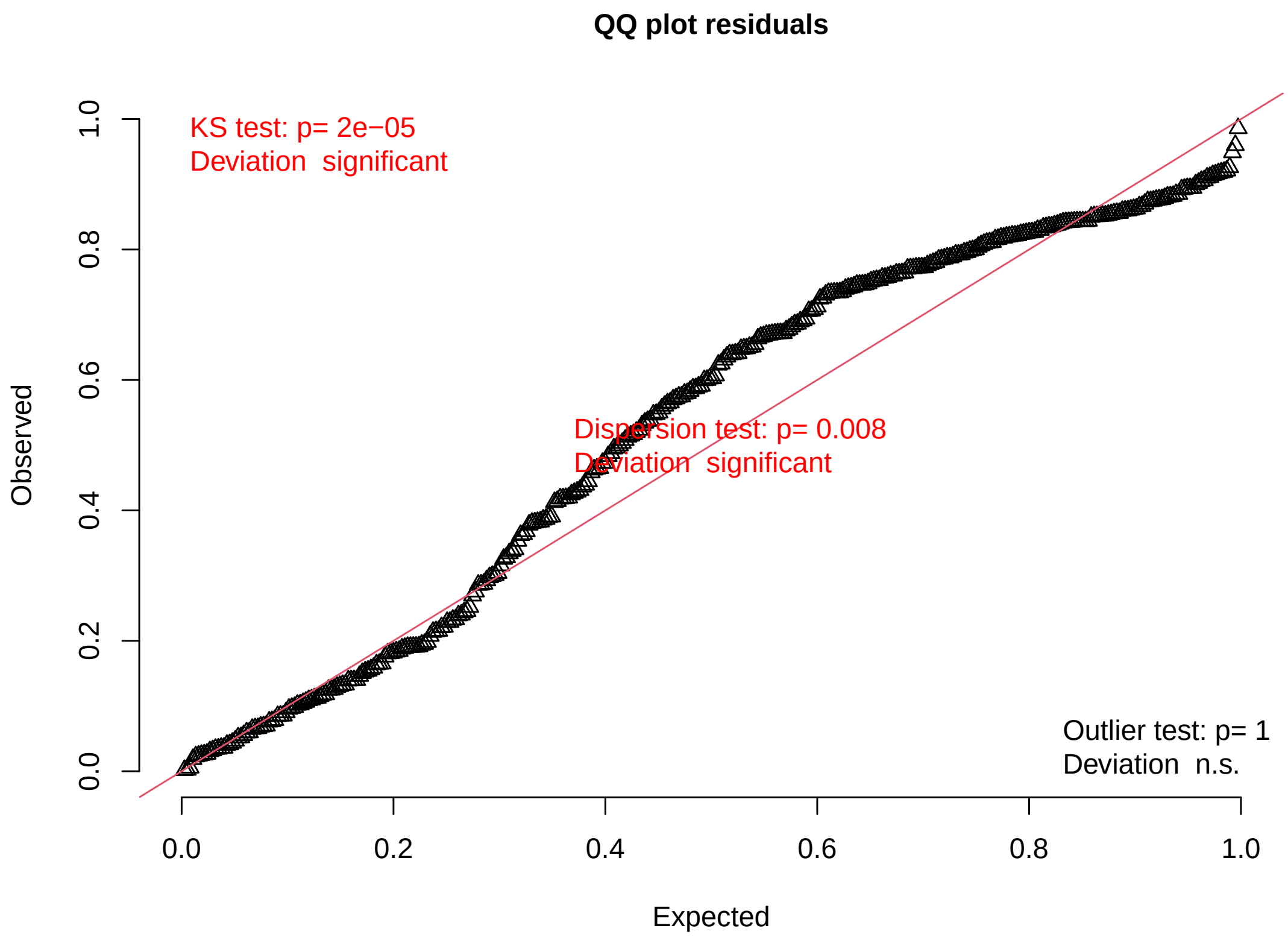


```
##
##  Asymptotic one-sample Kolmogorov-Smirnov test
##
## data:  simulationOutput$scaledResiduals
## D = 0.12369, p-value = 2.144e-05
## alternative hypothesis: two-sided
```

```
testDispersion(res_grazing_nb2_zi)
```

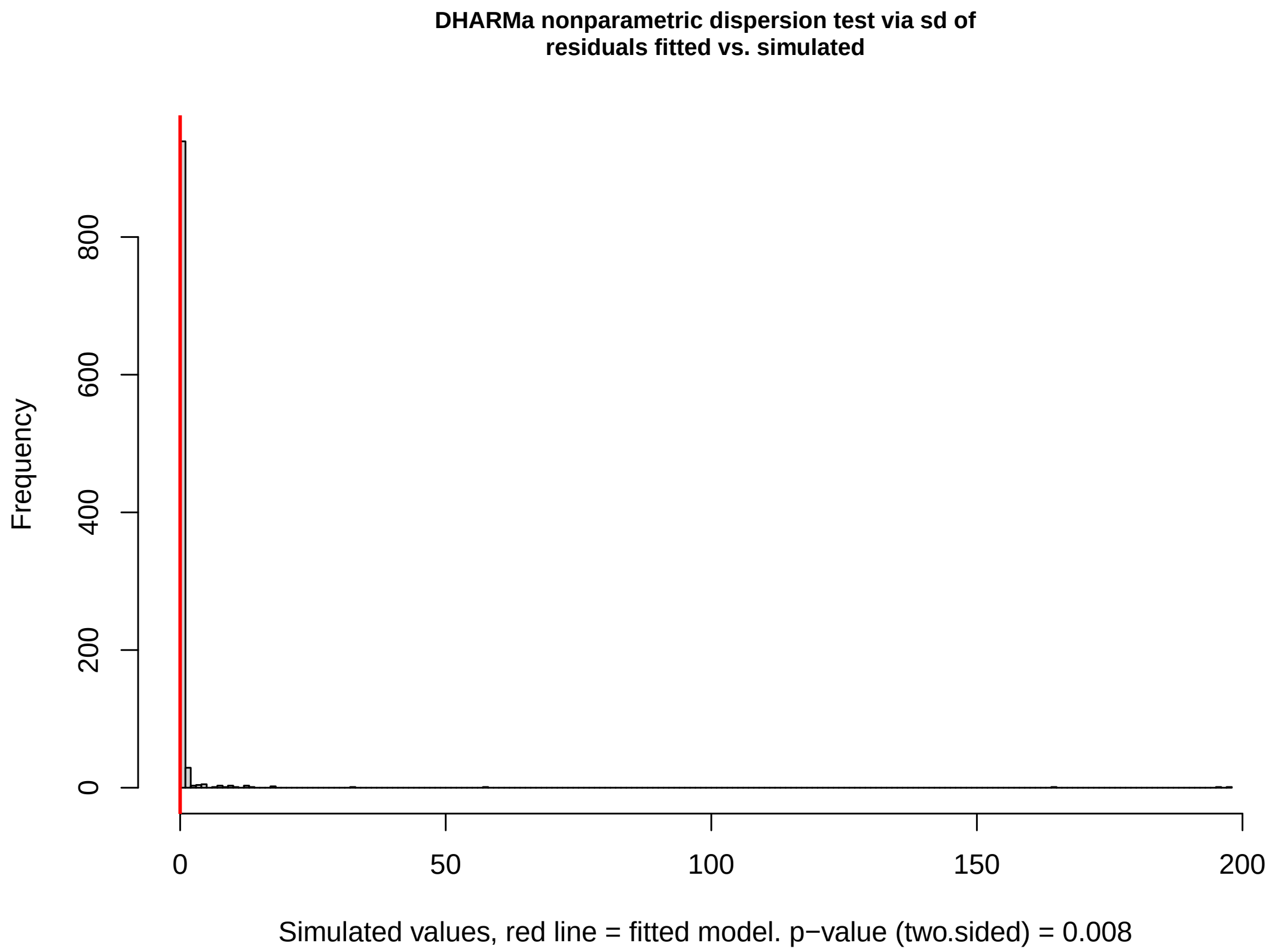


```
##
##  DHARMa nonparametric dispersion test via sd of residuals fitted vs.
##  simulated
##
## data:  simulationOutput
## dispersion = 0.0017819, p-value = 0.008
## alternative hypothesis: two.sided
```

```
testZeroInflation(res_grazing_nb2_zi)
```

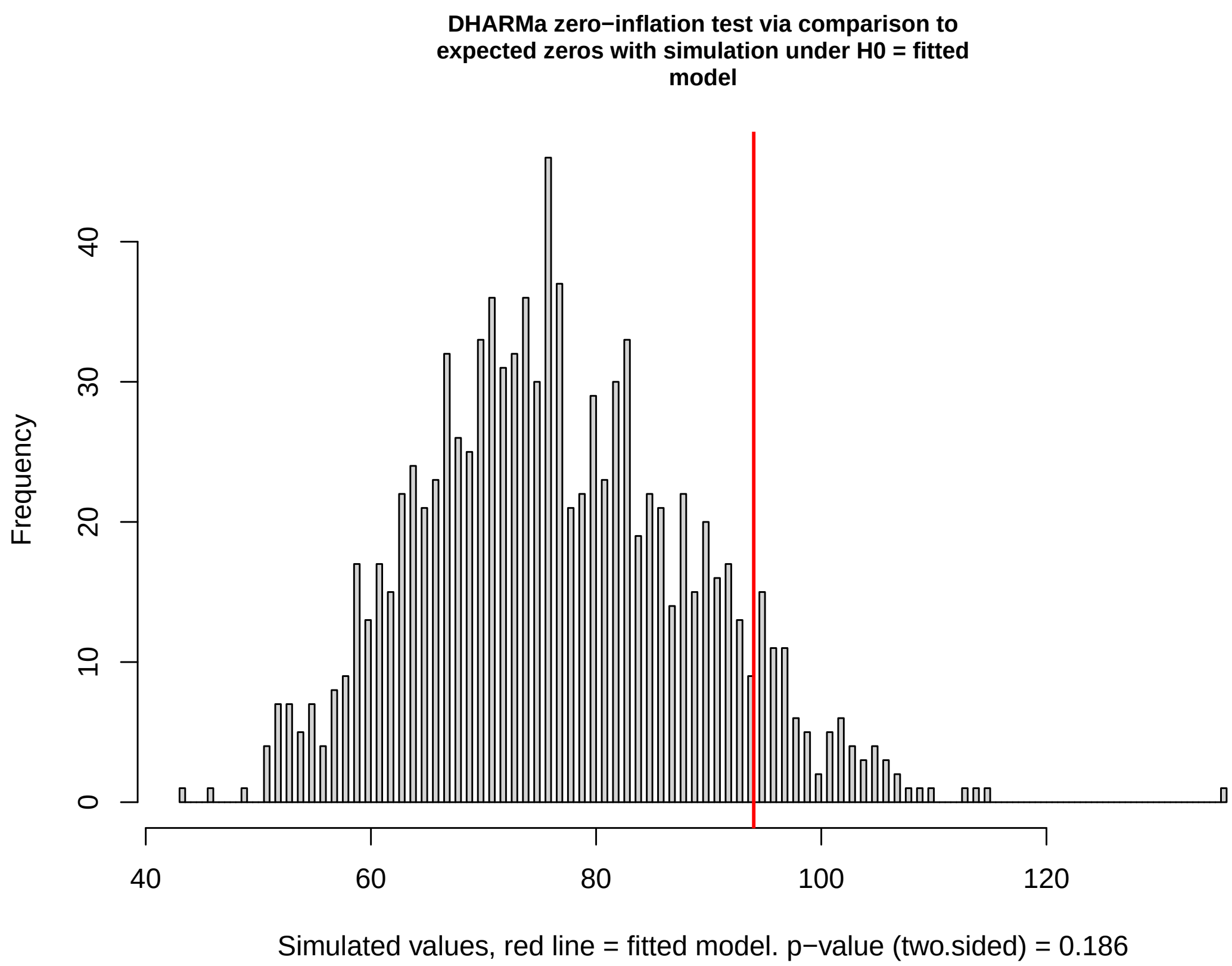


```
##
##  DHARMa zero-inflation test via comparison to expected zeros with
##  simulation under H0 = fitted model
##
## data:  simulationOutput
## ratioObsSim = 1.2265, p-value = 0.186
## alternative hypothesis: two.sided
```

```r
testOutliers(res_grazing_nb2_zi)
```

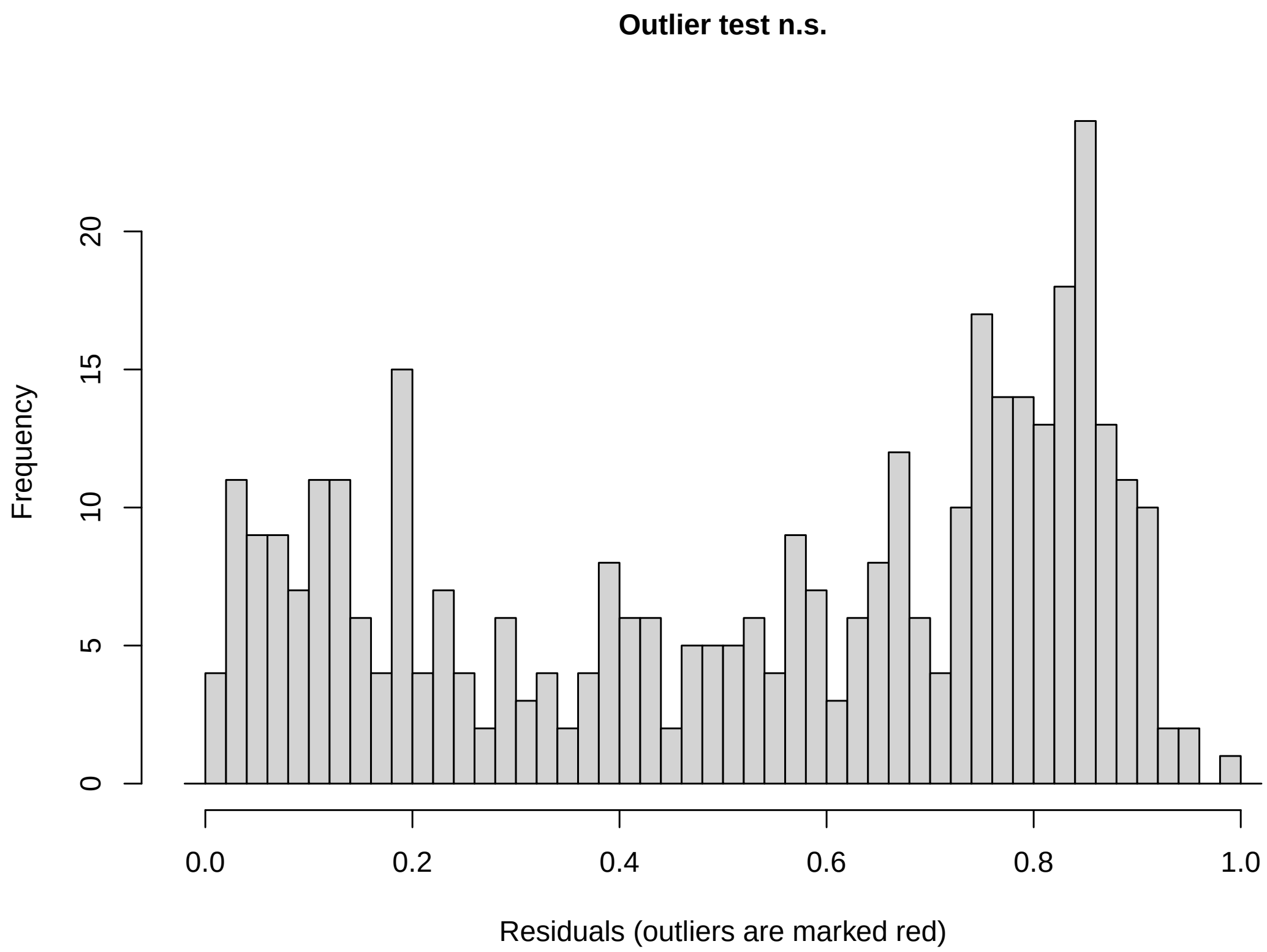


```
##
##  DHARMa bootstrapped outlier test
##
## data:  res_grazing_nb2_zi
## outliers at both margin(s) = 0, observations = 374, p-value = 1
## alternative hypothesis: two.sided
##  percent confidence interval:
##  0.00000000 0.01069519
## sample estimates:
## outlier frequency (expected: 0.00141711229946524 )
##                                                  0
```

4. Negative-binomial type 1 grazing model

The selected NB1 model is summarised below. Fixed-effect coefficients are reported on the log scale, while the random-effect estimates describe variation among fish and among feeding trials within fish.

```r
final_grazing_model <- m_grazing_nb1
summary(final_grazing_model)
```

```
##  Family: nbinom1  ( log )
## Formula:
## BiteCount ~ TileType + NH3_umol + DaysSinceStart_c + (1 | Fish/TrialID) +
##     offset(log(Time_recorded))
```

```
## Data: bites_long
##
##       AIC       BIC    logLik -2*log(L)  df.resid
##    3205.7    3233.1   -1595.8    3191.7       367
##
## Random effects:
##
## Conditional model:
##  Groups       Name        Variance Std.Dev.
##  TrialID:Fish (Intercept) 0.6943   0.8333
##  Fish         (Intercept) 1.1645   1.0791
## Number of obs: 374, groups:  TrialID:Fish, 187; Fish, 21
##
## Dispersion parameter for nbinom1 family (): 46.8
##
## Conditional model:
##                   Estimate Std. Error z value Pr(>|z|)
## (Intercept)      -1.46318    0.29725  -4.922 8.55e-07 ***
## TileTypeHNT       1.02351    0.09872  10.368  < 2e-16 ***
## NH3_umol          0.02792    0.04298   0.650  0.51589
## DaysSinceStart_c  0.04663    0.01712   2.723  0.00647 **
## ---
## Signif. codes:  0 '***' 0.001 '**' 0.01 '*' 0.05 '.' 0.1 ' ' 1
```

```r
VarCorr(final_grazing_model)
```

```
##
## Conditional model:
##  Groups       Name        Std.Dev.
##  TrialID:Fish (Intercept) 0.83327
##  Fish         (Intercept) 1.07913
```

4.1 Fixed-effect rate ratios and 95% confidence intervals

Because the model uses a log link, exponentiated coefficients are rate ratios.

```r
grazing_coef <- as.data.frame(
  summary(final_grazing_model)$coefficients$cond
) %>%
  tibble::rownames_to_column("Term") %>%
  rename(
    Estimate = Estimate,
    SE = `Std. Error`,
    z_value = `z value`,
    p_value = `Pr(>|z|)`
  ) %>%
  mutate(
    CI_low_log = Estimate - 1.96 * SE,
    CI_high_log = Estimate + 1.96 * SE,
    Rate_ratio = exp(Estimate),
    RR_CI_low = exp(CI_low_log),
    RR_CI_high = exp(CI_high_log)
```

```
  )

grazing_coef
```

```
##              Term    Estimate         SE    z_value      p_value  CI_low_log
## 1     (Intercept) -1.46318413 0.29725088 -4.9223878 8.549458e-07 -2.04579586
## 2     TileTypeHNT  1.02350841 0.09871726 10.3680794 3.464172e-25  0.83002258
## 3        NH3_umol  0.02792430 0.04298101  0.6496894 5.158929e-01 -0.05631847
## 4 DaysSinceStart_c  0.04663138 0.01712430  2.7231122 6.467010e-03  0.01306776
##   CI_high_log Rate_ratio RR_CI_low RR_CI_high
## 1  -0.8805724   0.231498 0.1292773  0.4145456
## 2   1.2169942   2.782941 2.2933705  3.3770220
## 3   0.1121671   1.028318 0.9452381  1.1186998
## 4   0.0801950   1.047736 1.0131535  1.0834983
```

5. Model-predicted grazing rates

Population-level predictions were generated to visualise grazing rates in relation to tile type, measured NH3 concentration, and experimental time.

As the recording duration is included as an offset, predictions were generated with `Time_recorded = 1`, so predicted values are expresed as bites per minute. Population-level predictions are generated with random effects set to zero (`re.form = NA`).

5.1 Population-level prediction function

```
predict_population <- function(model, newdata) {
  pred <- predict(
    model,
    newdata = newdata,
    type = "link",
    se.fit = TRUE,
    re.form = NA,
    allow.new.levels = TRUE
  )

  newdata %>%
    mutate(
      fit_link = as.numeric(pred$fit),
      se_link = as.numeric(pred$se.fit),
      predicted = exp(fit_link),
      conf.low = exp(fit_link - 1.96 * se_link),
      conf.high = exp(fit_link + 1.96 * se_link)
    )
}
```

5.2 Panel A: HNT versus LNT grazing rates

```r
tile_pred_data <- expand_grid(
  TileType = factor(c("LNT", "HNT"), levels = c("LNT", "HNT")),
  NH3_umol = median(bites_long$NH3_umol, na.rm = TRUE),
  DaysSinceStart_c = 0,
  Time_recorded = 1,
  Fish = levels(bites_long$Fish)[1],
  TrialID = levels(bites_long$TrialID)[1]
)

tile_predictions <- predict_population(final_grazing_model, tile_pred_data)
tile_predictions
```

```
## # A tibble: 2 x 11
##   TileType NH3_umol DaysSinceStart_c Time_recorded Fish  TrialID      fit_link
##   <fct>       <dbl>            <dbl>         <dbl> <chr> <chr>           <dbl>
## 1 LNT          1.08                0             1 3     3.2023-04-25   -1.43
## 2 HNT          1.08                0             1 3     3.2023-04-25   -0.410
## # i 4 more variables: se_link <dbl>, predicted <dbl>, conf.low <dbl>,
## #   conf.high <dbl>
```

```r
tile_colours <- c(
  "LNT" = "#7F6524",
  "HNT" = "#247F65"
)

plot_tile <- ggplot() +
  geom_jitter(
    data = bites_long,
    aes(
      x = TileType,
      y = BiteRate,
      colour = TileType
    ),
    width = 0.12,
    alpha = 0.22,
    size = 1.4
  ) +
  geom_point(
    data = tile_predictions,
    aes(
      x = TileType,
      y = predicted,
      colour = TileType
    ),
    size = 3
  ) +
  geom_errorbar(
    data = tile_predictions,
    aes(
      x = TileType,
      ymin = conf.low,
      ymax = conf.high,
```

```
      colour = TileType
    ),
    width = 0.08,
    linewidth = 0.7
  ) +
  scale_colour_manual(values = tile_colours) +
  labs(
    x = "Tile type",
    y = expression("Grazing rate (bites min"^{-1}*")")
  ) +
  theme_classic() +
  theme(
  legend.position = "none"
)

plot_tile <- plot_tile +
  guides(colour = "none")

plot_tile
```

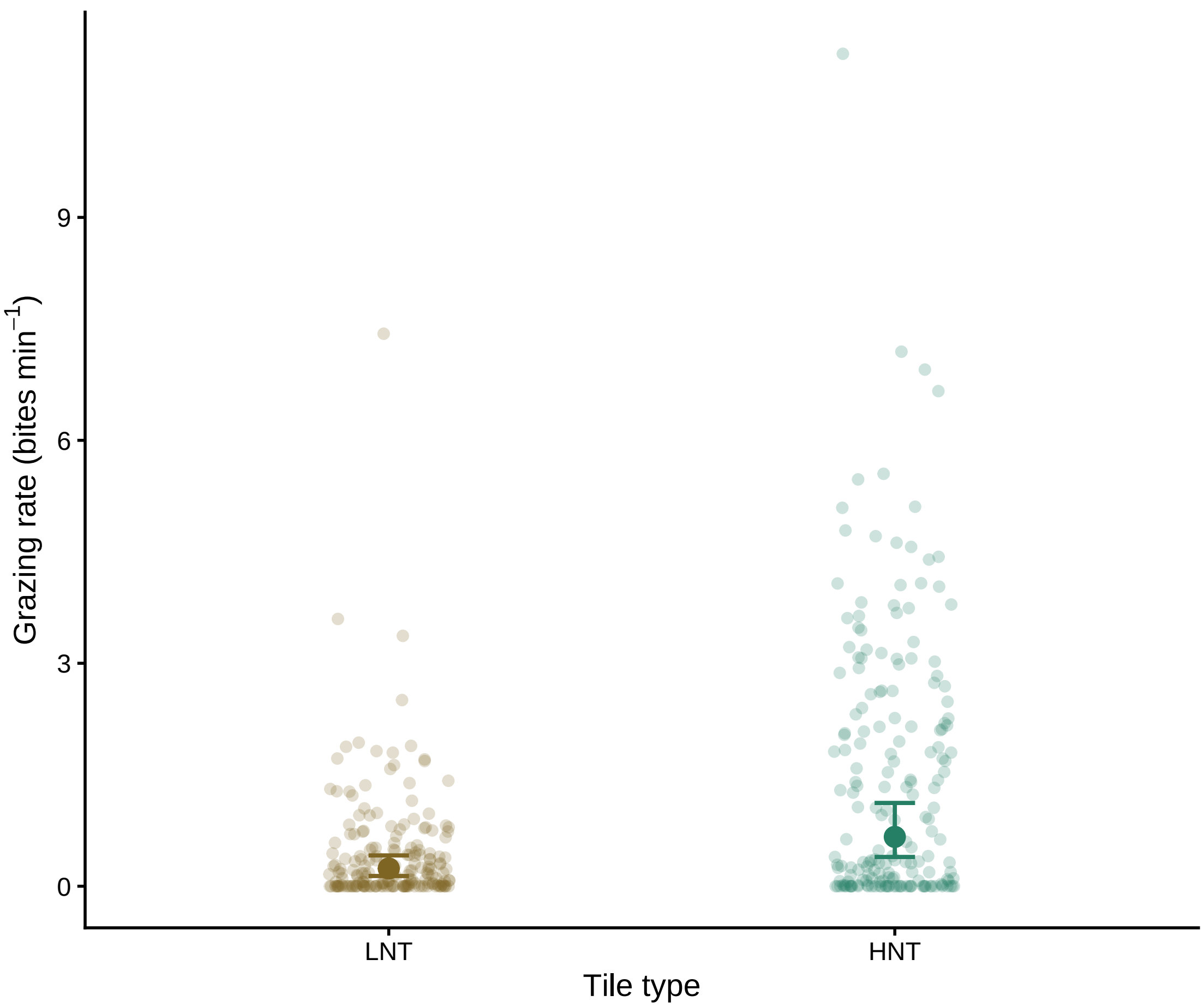

5.3 Panel B: grazing rate across ammonia concentration

Because the selected model is additive and contains no `TileType × NH3` interaction, HNT and LNT have the same estimated NH3 slope but different predicted grazing-rate levels.

```
nh3_sequence <- seq(
  min(bites_long$NH3_umol, na.rm = TRUE),
  max(bites_long$NH3_umol, na.rm = TRUE),
  length.out = 100
)

nh3_pred_data <- expand_grid(
  TileType = factor(c("LNT", "HNT"), levels = c("LNT", "HNT")),
  NH3_umol = nh3_sequence,
  DaysSinceStart_c = 0,
  Time_recorded = 1,
  Fish = levels(bites_long$Fish)[1],
  TrialID = levels(bites_long$TrialID)[1]
)

nh3_predictions <- predict_population(final_grazing_model, nh3_pred_data)
```

```
plot_nh3 <- ggplot() +
  geom_point(
    data = bites_long,
    aes(
      x = NH3_umol,
      y = BiteRate,
      colour = TileType
    ),
    alpha = 0.20,
    size = 1.3
  ) +
  geom_ribbon(
    data = nh3_predictions,
    aes(
      x = NH3_umol,
      ymin = conf.low,
      ymax = conf.high,
      fill = TileType,
      group = TileType
    ),
    alpha = 0.15,
    colour = NA
  ) +
  geom_line(
    data = nh3_predictions,
    aes(
      x = NH3_umol,
      y = predicted,
      colour = TileType
    ),
    linewidth = 0.9
  ) +
```

```
  scale_colour_manual(values = tile_colours) +
  scale_fill_manual(values = tile_colours) +
  labs(
    x = expression(NH[3]~"concentration ("*mu*"M)"),
    y = expression("Grazing rate (bites min"^{-1}*")"),
    colour = "Tile type",
    fill = "Tile type"
  ) +
  theme_classic()

plot_nh3
```

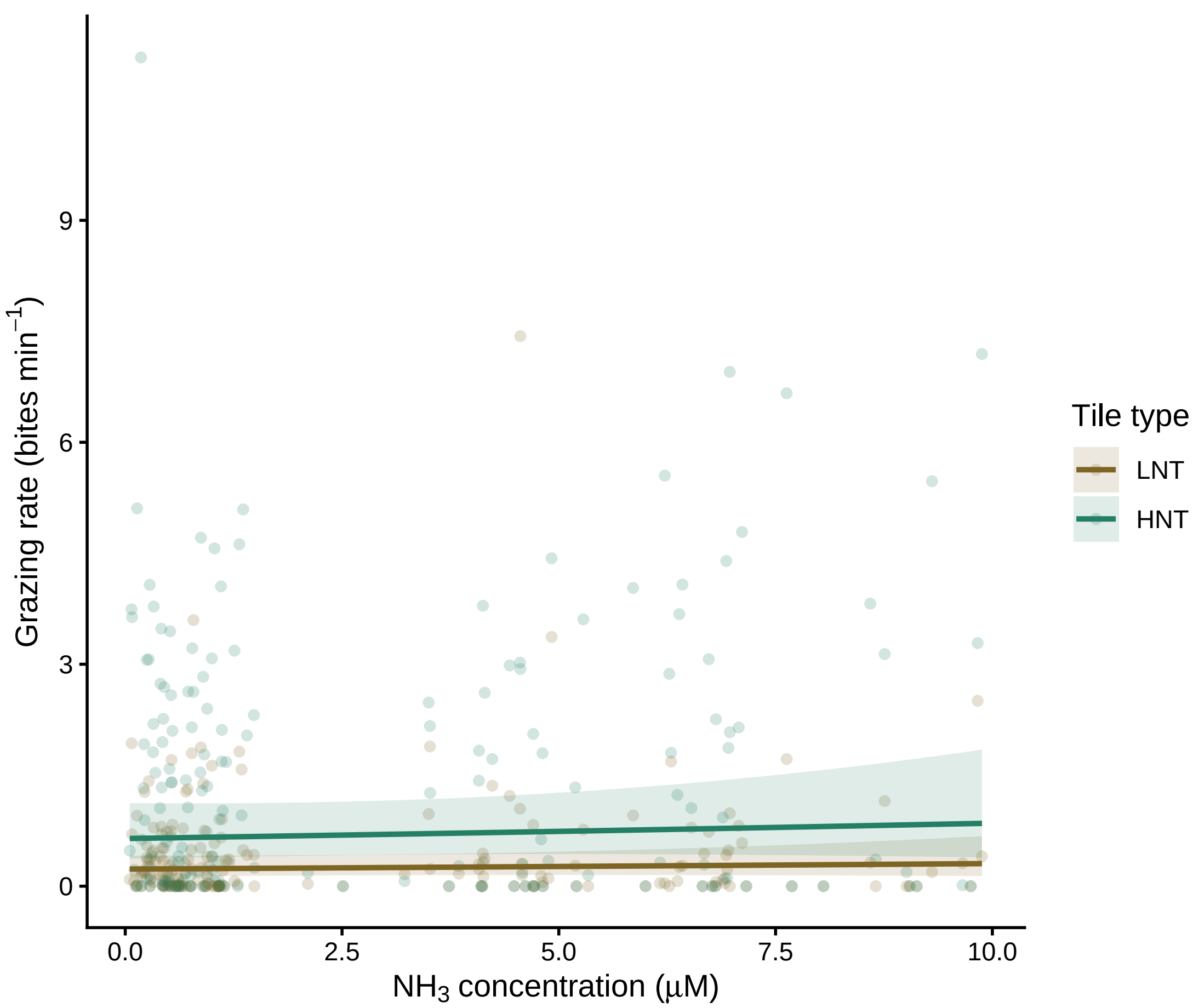

5.4 Panel C: grazing rate across experimental time

```
time_lookup <- bites_long %>%
  distinct(Date, DaysSinceStart, DaysSinceStart_c) %>%
  arrange(Date)

time_pred_data <- expand_grid(
  TileType = factor(c("LNT", "HNT"), levels = c("LNT", "HNT")),
  DaysSinceStart_c = time_lookup$DaysSinceStart_c,
```

```r
  NH3_umol = median(bites_long$NH3_umol, na.rm = TRUE),
  Time_recorded = 1,
  Fish = levels(bites_long$Fish)[1],
  TrialID = levels(bites_long$TrialID)[1]
) %>%
  left_join(time_lookup, by = "DaysSinceStart_c")

time_predictions <- predict_population(final_grazing_model, time_pred_data)
```

```r
plot_time <- ggplot() +
  geom_point(
    data = bites_long,
    aes(
      x = Date,
      y = BiteRate,
      colour = TileType
    ),
    alpha = 0.20,
    size = 1.3
  ) +
  geom_ribbon(
    data = time_predictions,
    aes(
      x = Date,
      ymin = conf.low,
      ymax = conf.high,
      fill = TileType,
      group = TileType
    ),
    alpha = 0.15,
    colour = NA
  ) +
  geom_line(
    data = time_predictions,
    aes(
      x = Date,
      y = predicted,
      colour = TileType
    ),
    linewidth = 0.9
  ) +
  scale_colour_manual(values = tile_colours) +
  scale_fill_manual(values = tile_colours) +
  scale_x_date(
    date_breaks = "2 days",
    date_labels = "%d-%b"
  ) +
  labs(
    x = "Date",
    y = expression("Grazing rate (bites min"^{-1}*")"),
    colour = "Tile type",
    fill = "Tile type"
  ) +
```

```r
  theme_classic() +
  theme(
    axis.text.x = element_text(
      angle = 45,
      hjust = 1
    )
  )

plot_time
```

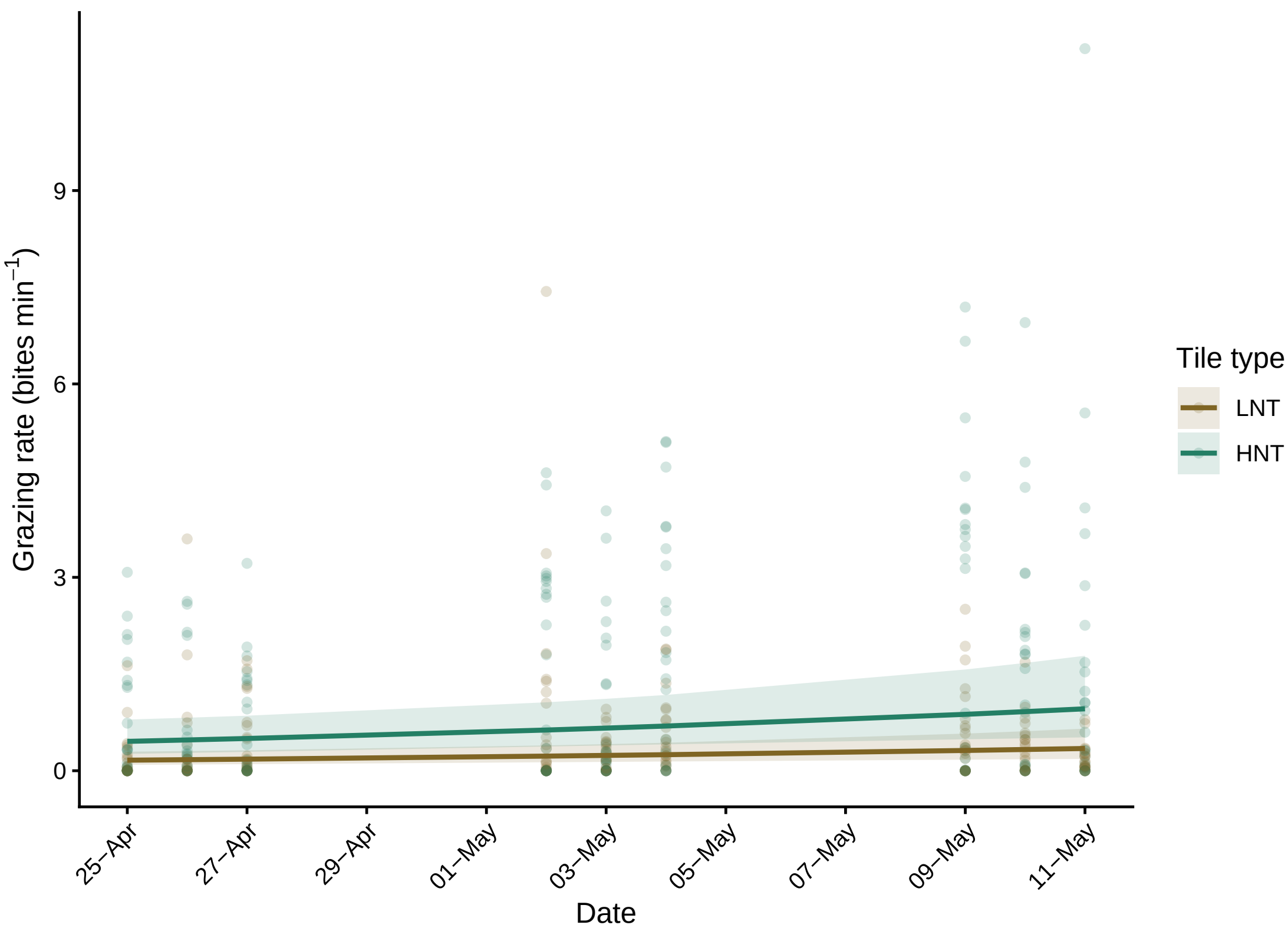

5.5 Combined figure

```r
primary_figure <-
  plot_tile /
  plot_nh3 /
  plot_time +
  plot_annotation(tag_levels = "a") +
  plot_layout(guides = "collect") &
  theme(
    legend.position = "right",
    legend.title = element_text(size = 11),
    legend.text = element_text(size = 10)
  )

primary_figure
```

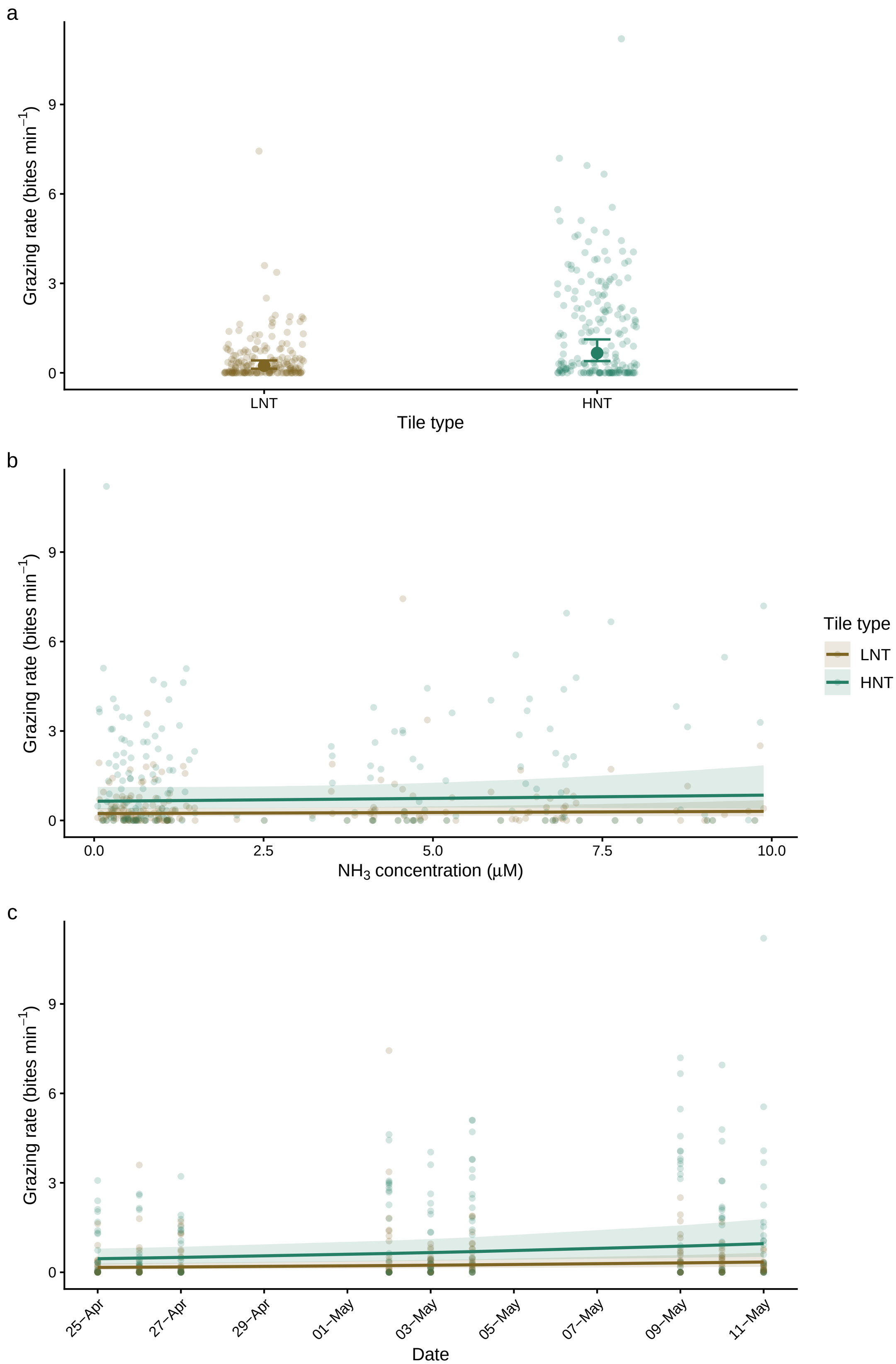
a
Grazing rate (bites min^{-1})
0
3
6
9
LNT
HNT
Tile type
b
Grazing rate (bites min^{-1})
0.0
2.5
5.0
7.5
10.0
NH_3 concentration (µM)
Tile type
LNT
HNT
c
Grazing rate (bites min^{-1})
25–Apr
27–Apr
29–Apr
01–May
03–May
05–May
07–May
09–May
11–May
Date

```r
ggsave(
  "Figure_Grazing_NestedModel.png",
  primary_figure,
  width = 8,
  height = 12,
  dpi = 600
)
```

6. Food-selectivity analysis

The tile-level grazing model evaluates absolute differences in grazing rate between HNT and LNT. Food selectivity was additionally examined as the relative allocation of bites between the two simultaneously offered tiles. This conditional analysis was restricted to trials in which at least one bite occurred, because trials with zero bites on both tiles contain no information about relative bite allocation.

The selectivity model addressed whether bites were preferentially allocated to HNT and whether this allocation varied with measured NH3 concentration or experimental time.

6.1 Binomial and beta-binomial candidate models

```r
selectivity_formula <- cbind(Bites_HNT, Bites_LNT) ~
  NH3_umol +
  DaysSinceStart_c +
  (1 | Fish)

m_select_binom <- glmmTMB(
  formula = selectivity_formula,
  data = selectivity_trial,
  family = binomial(link = "logit")
)

m_select_beta <- glmmTMB(
  formula = selectivity_formula,
  data = selectivity_trial,
  family = betabinomial(link = "logit")
)

selectivity_aic <- AIC(m_select_binom, m_select_beta) %>%
  as.data.frame() %>%
  arrange(AIC) %>%
  mutate(deltaAIC = AIC - min(AIC))

selectivity_aic
```

```
##                df      AIC deltaAIC
## m_select_beta   5 1214.256     0.00
## m_select_binom  4 3460.105  2245.85
```

6.2 DHARMa diagnostics

```
set.seed(123)

res_select_binom <- simulateResiduals(m_select_binom, n = 1000, plot = FALSE)
res_select_beta <- simulateResiduals(m_select_beta, n = 1000, plot = FALSE)

plot(res_select_binom)
```

```
## Warning in newton(lsp = lsp, X = G$X, y = G$y, Eb = G$Eb, UrS = G$UrS, L = G$L,
## : Fitting terminated with step failure - check results carefully
```

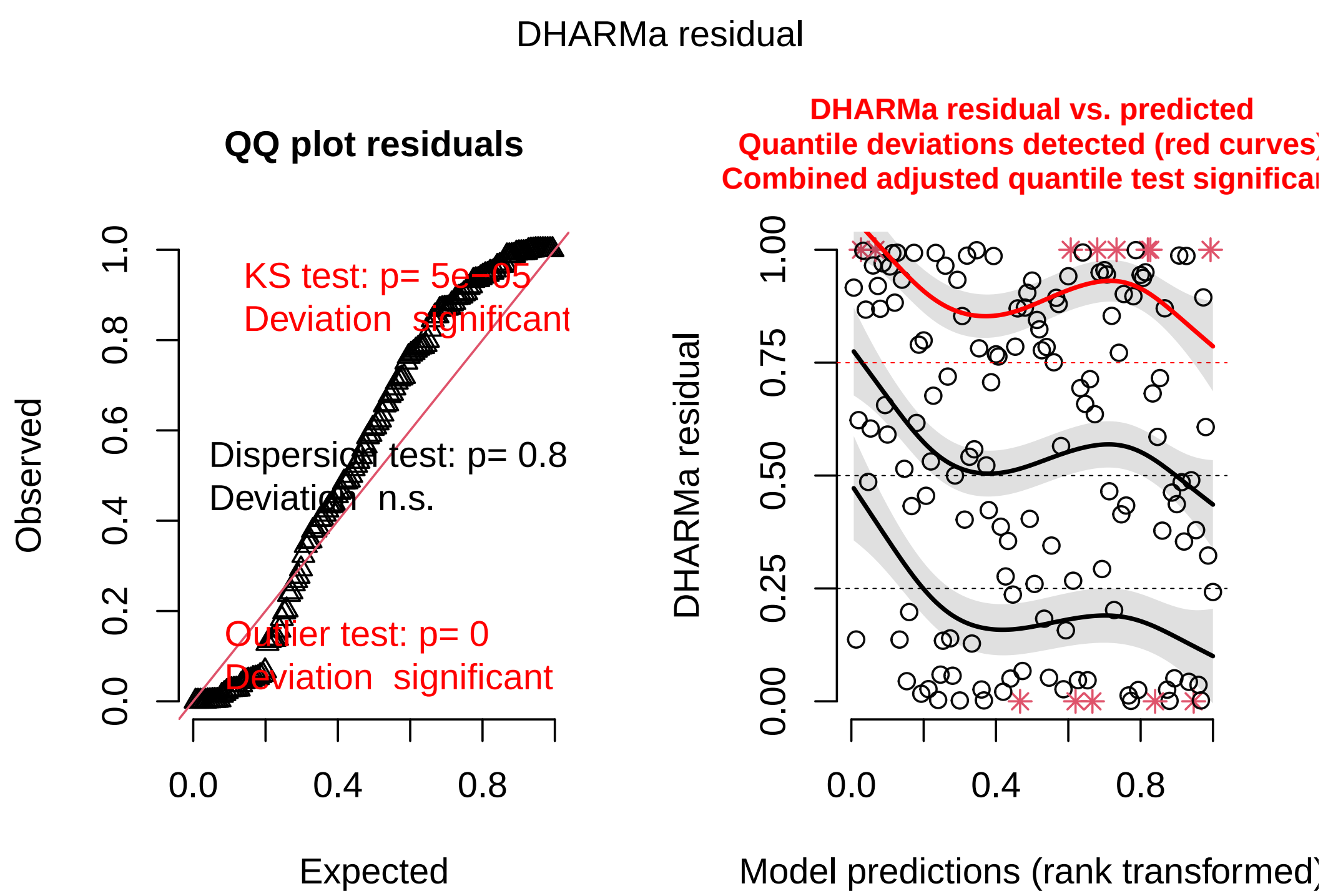


```
testUniformity(res_select_binom)
```

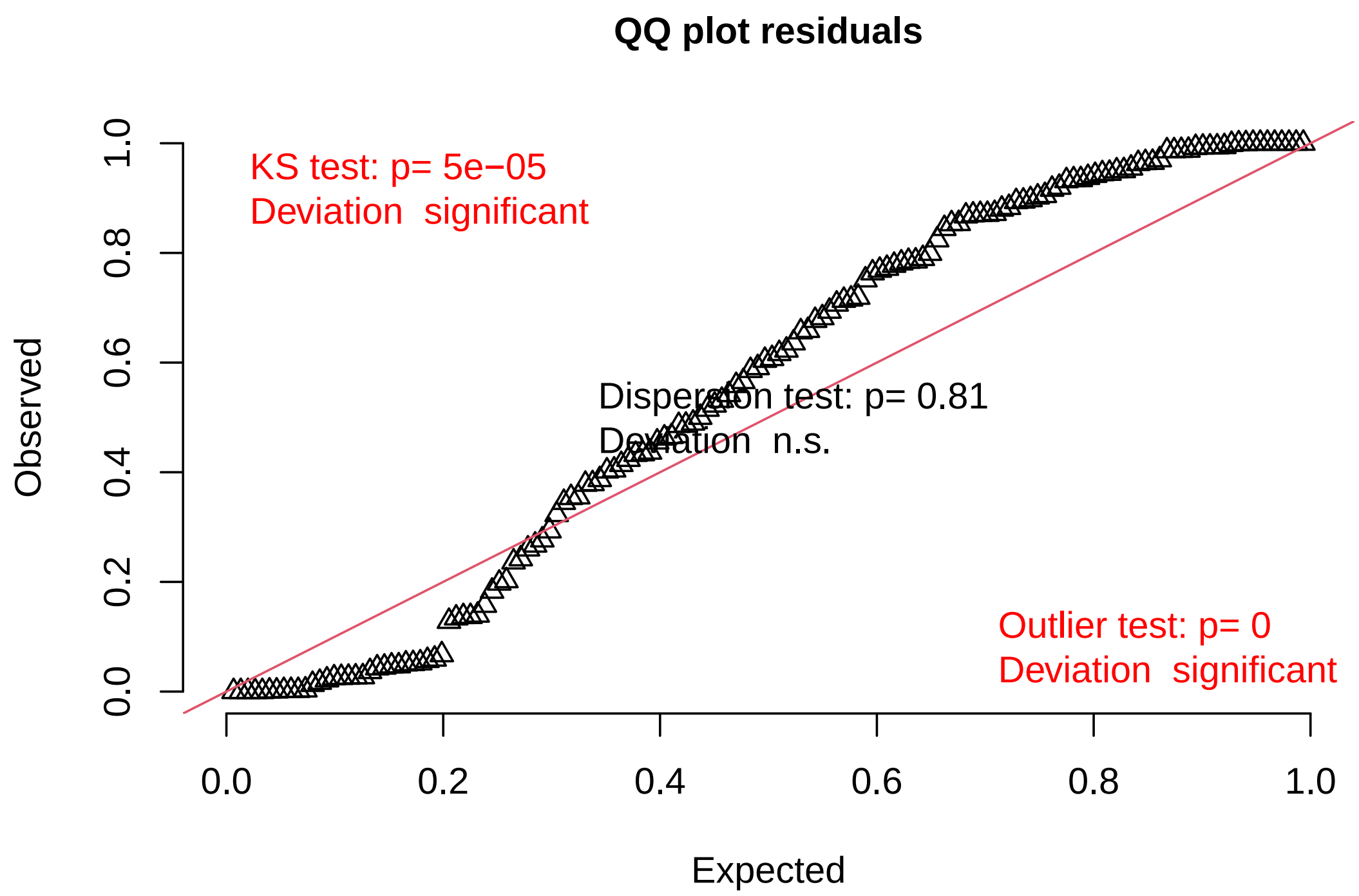


```
##
##  Asymptotic one-sample Kolmogorov-Smirnov test
##
## data:  simulationOutput$scaledResiduals
## D = 0.18747, p-value = 5.275e-05
## alternative hypothesis: two-sided
```

```
testDispersion(res_select_binom)
```

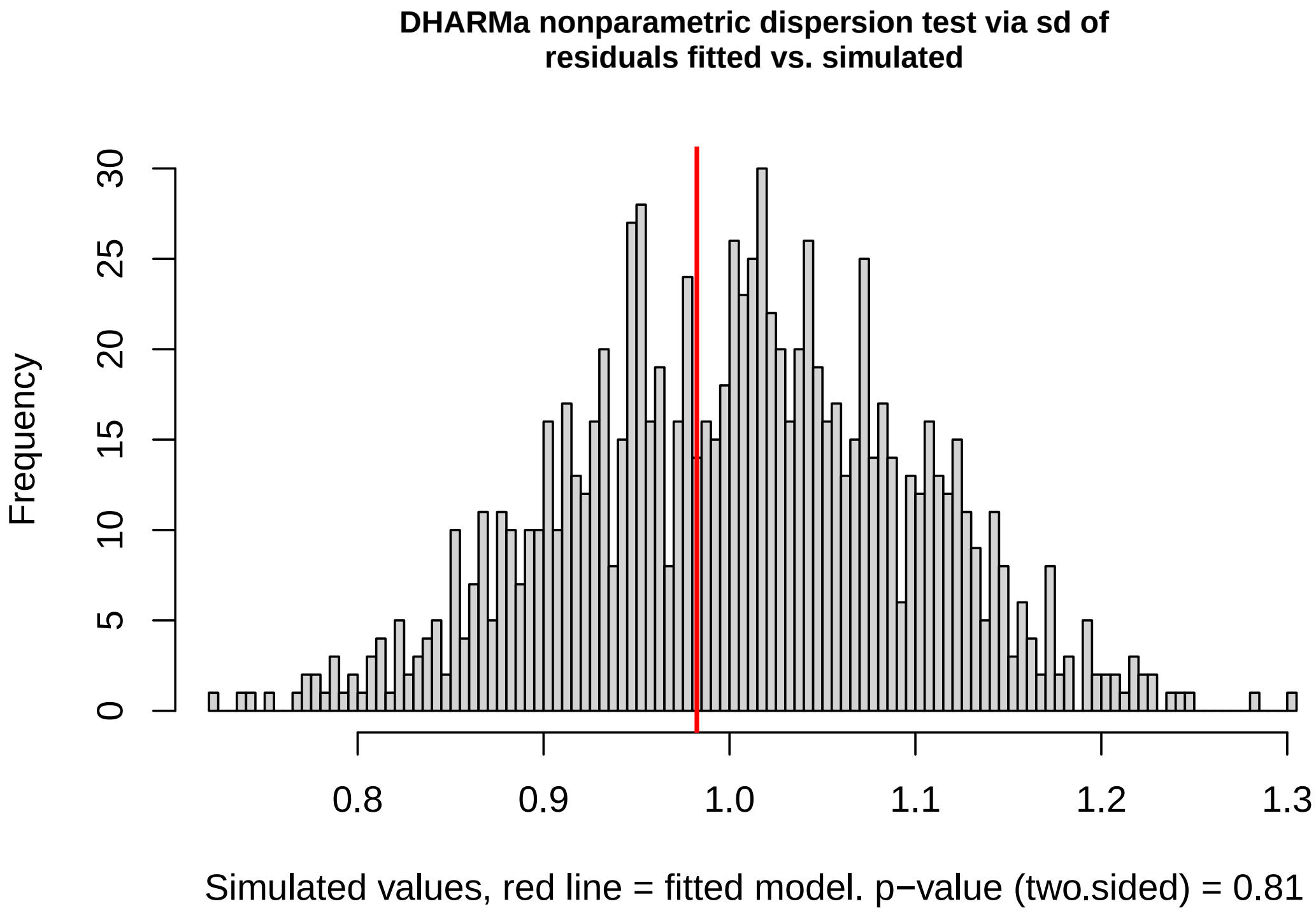


```
##
##  DHARMa nonparametric dispersion test via sd of residuals fitted vs.
##  simulated
##
## data:  simulationOutput
## dispersion = 0.97777, p-value = 0.81
## alternative hypothesis: two.sided
```

```
testZeroInflation(res_select_binom)
```

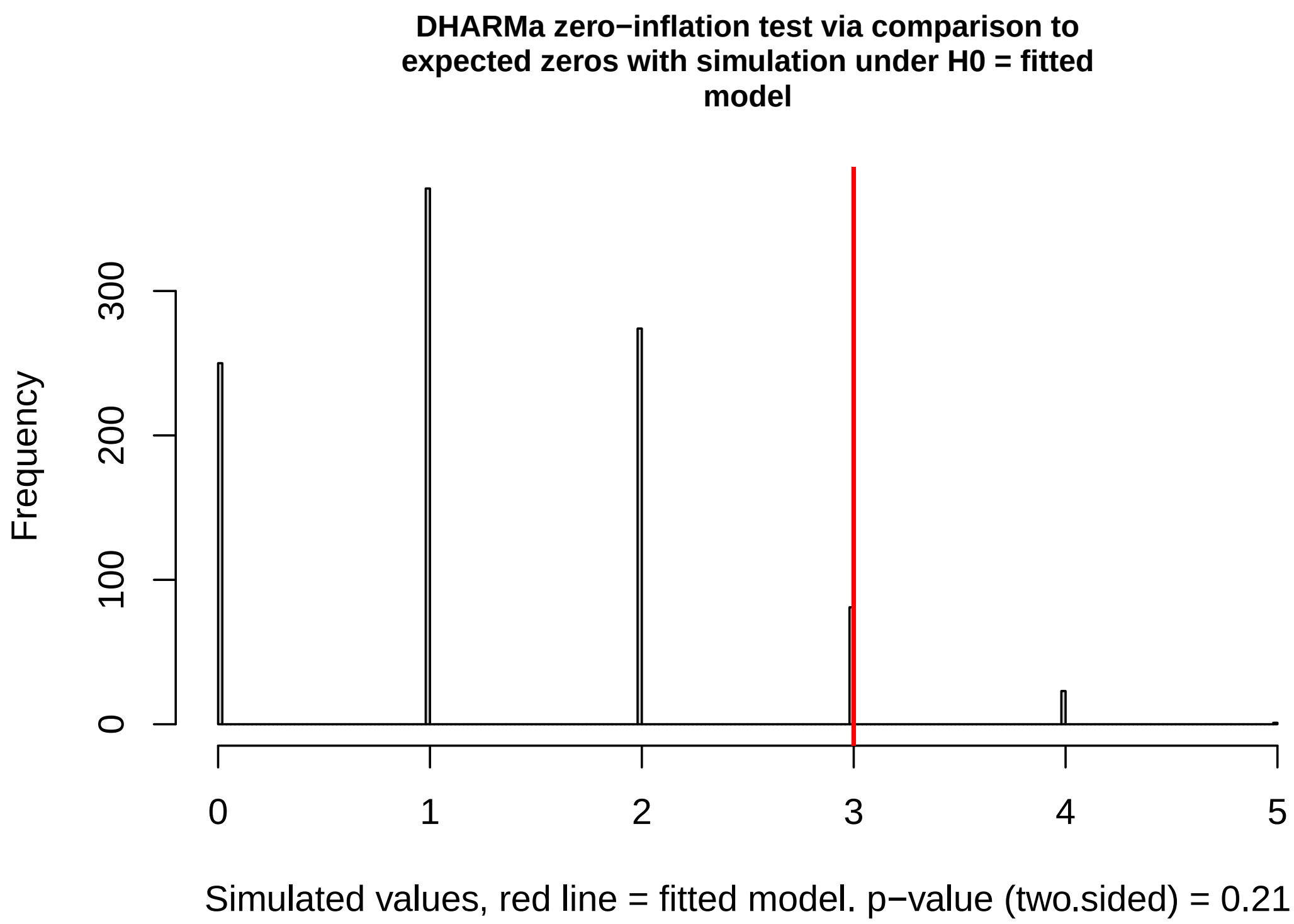


```
##
##  DHARMa zero-inflation test via comparison to expected zeros with
##  simulation under H0 = fitted model
##
## data:  simulationOutput
## ratioObsSim = 2.3828, p-value = 0.21
## alternative hypothesis: two.sided
```

```
testOutliers(res_select_binom)
```

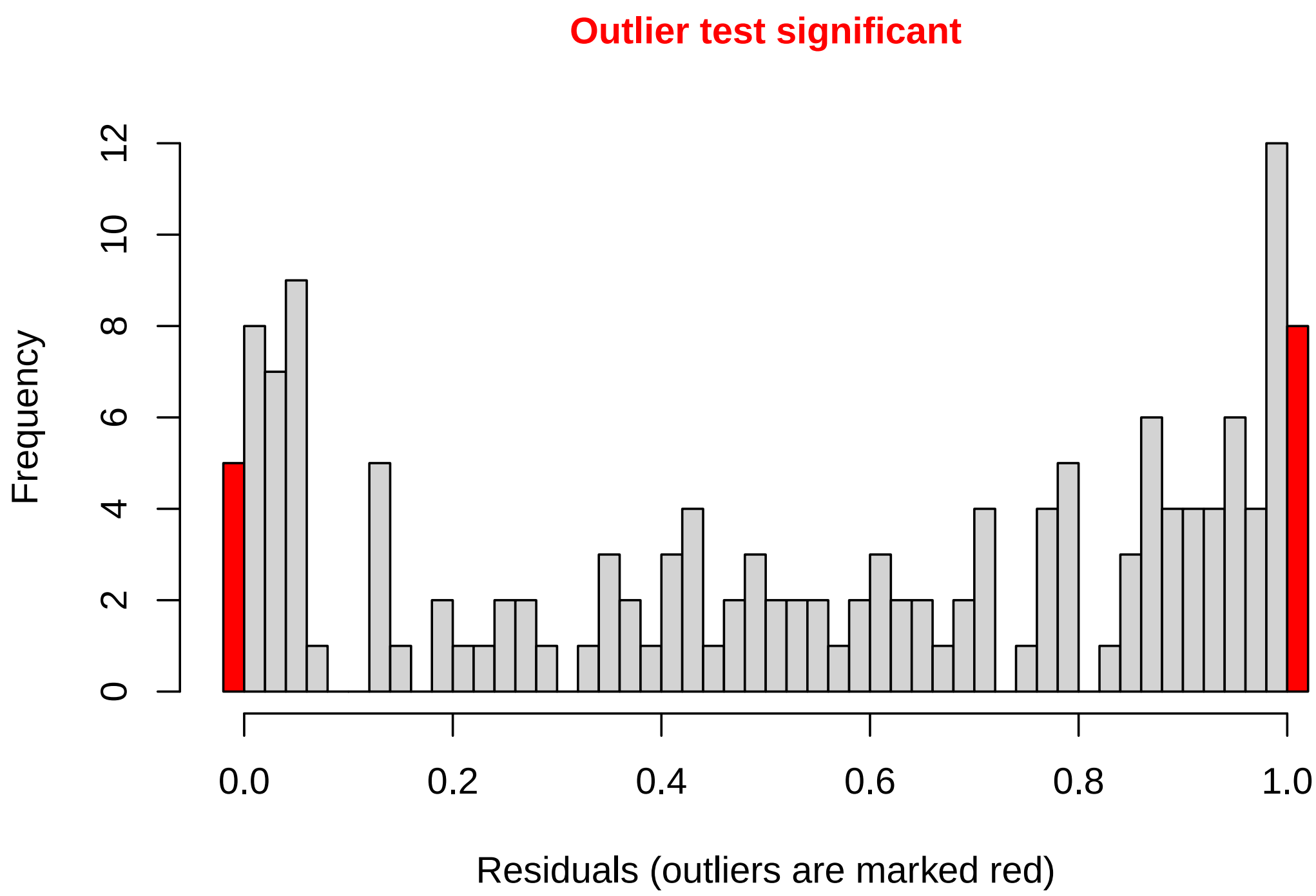


```
##
##  DHARMa bootstrapped outlier test
##
## data:  res_select_binom
## outliers at both margin(s) = 13, observations = 150, p-value < 2.2e-16
## alternative hypothesis: two.sided
##  percent confidence interval:
##  0.00 0.02
## sample estimates:
## outlier frequency (expected: 0.0016 )
##                              0.08666667
```

```
plot(res_select_beta)
```

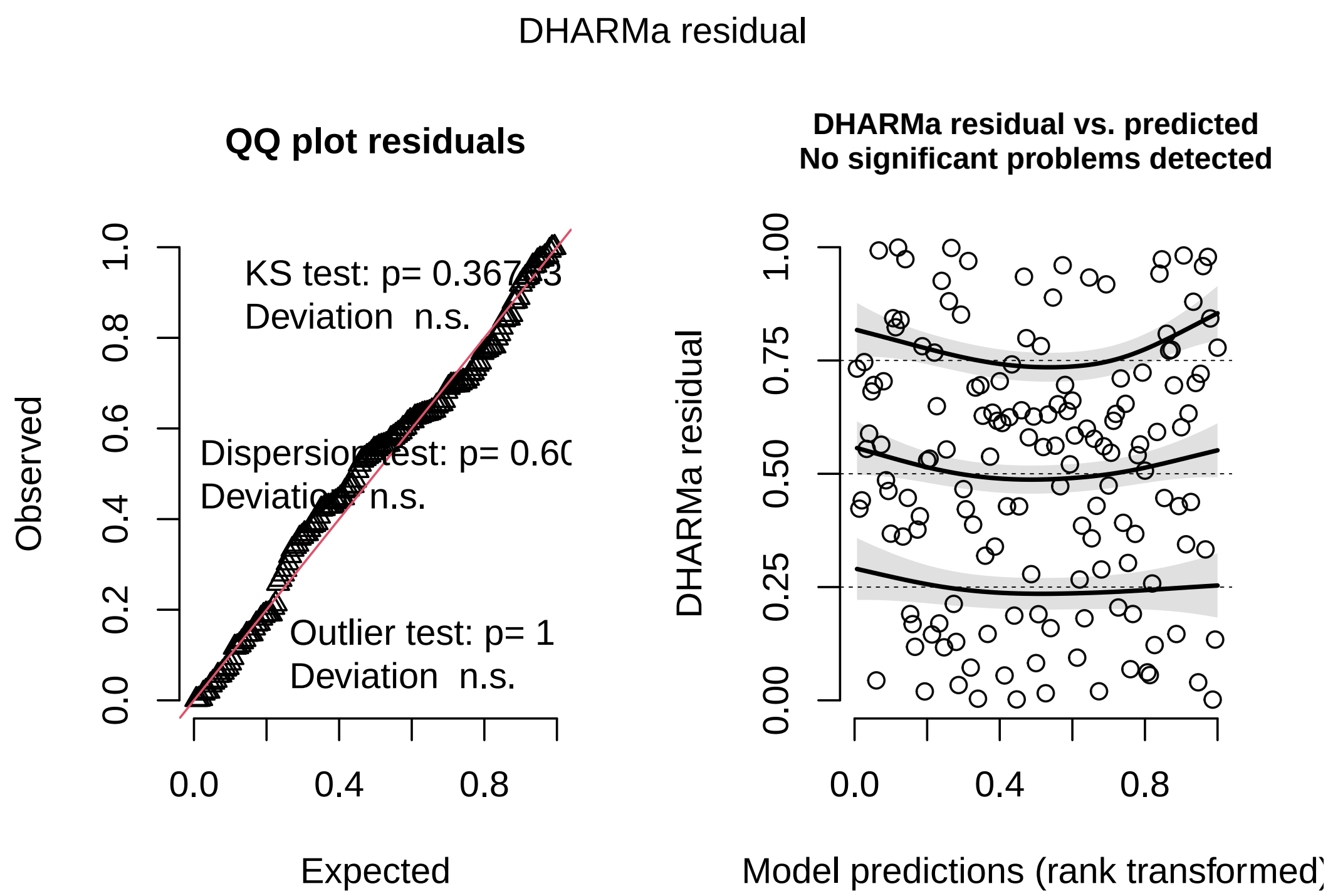


```
testUniformity(res_select_beta)
```

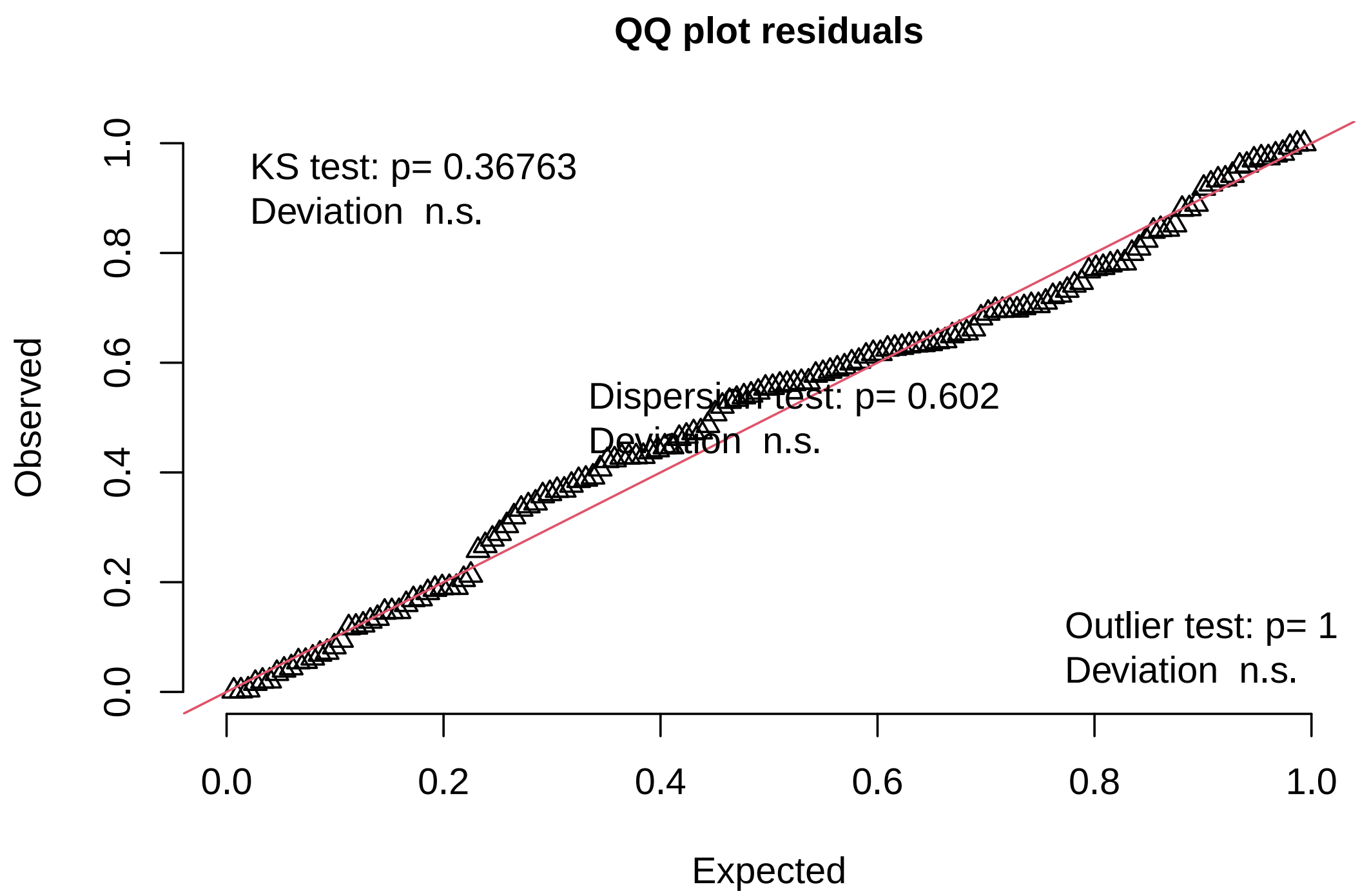


```
##
```

```
##  Asymptotic one-sample Kolmogorov-Smirnov test
##
## data:  simulationOutput$scaledResiduals
## D = 0.075, p-value = 0.3676
## alternative hypothesis: two-sided
```

```r
testDispersion(res_select_beta)
```

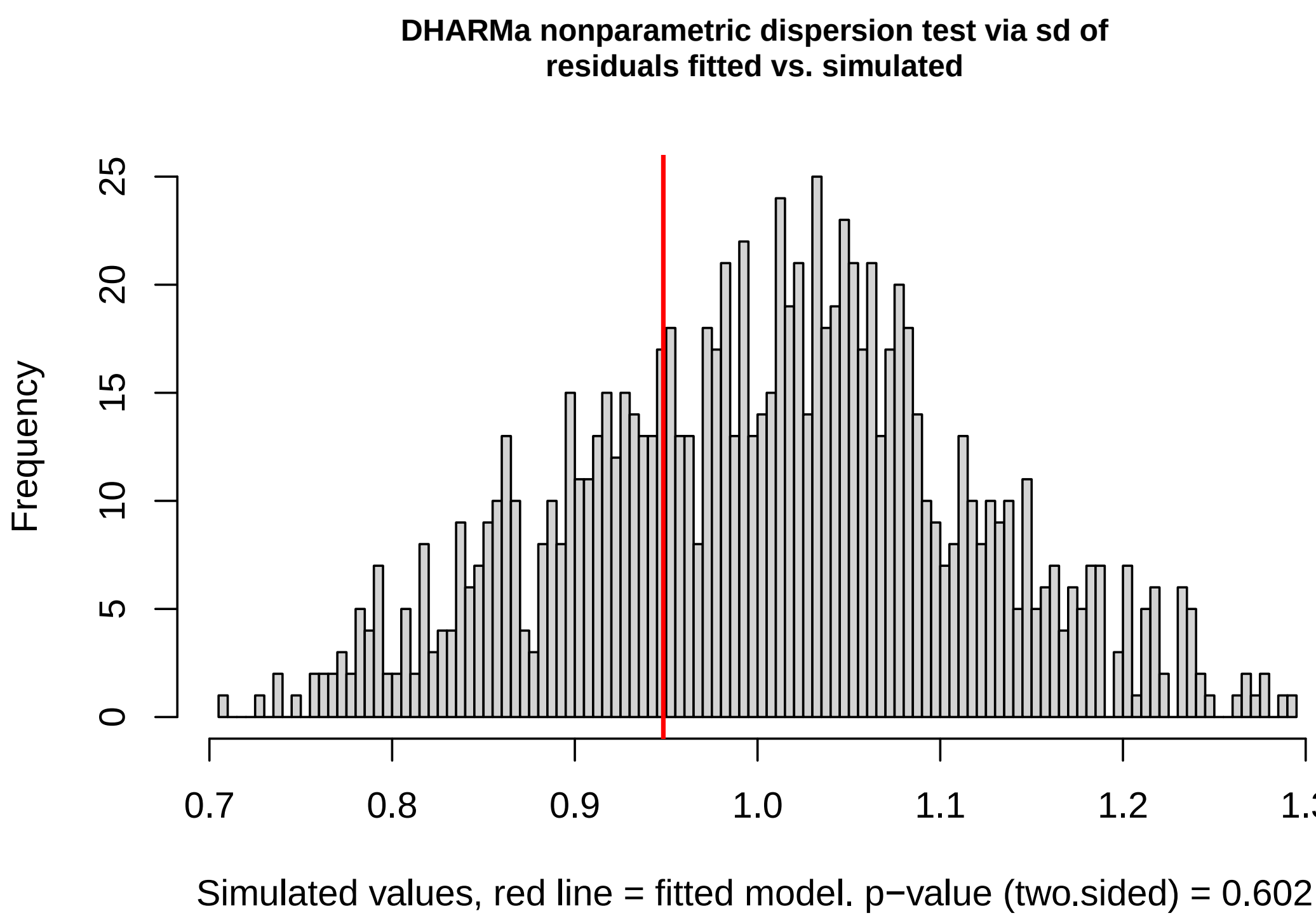


```
##
##  DHARMa nonparametric dispersion test via sd of residuals fitted vs.
##  simulated
##
## data:  simulationOutput
## dispersion = 0.94328, p-value = 0.602
## alternative hypothesis: two.sided
```

```r
testZeroInflation(res_select_beta)
```

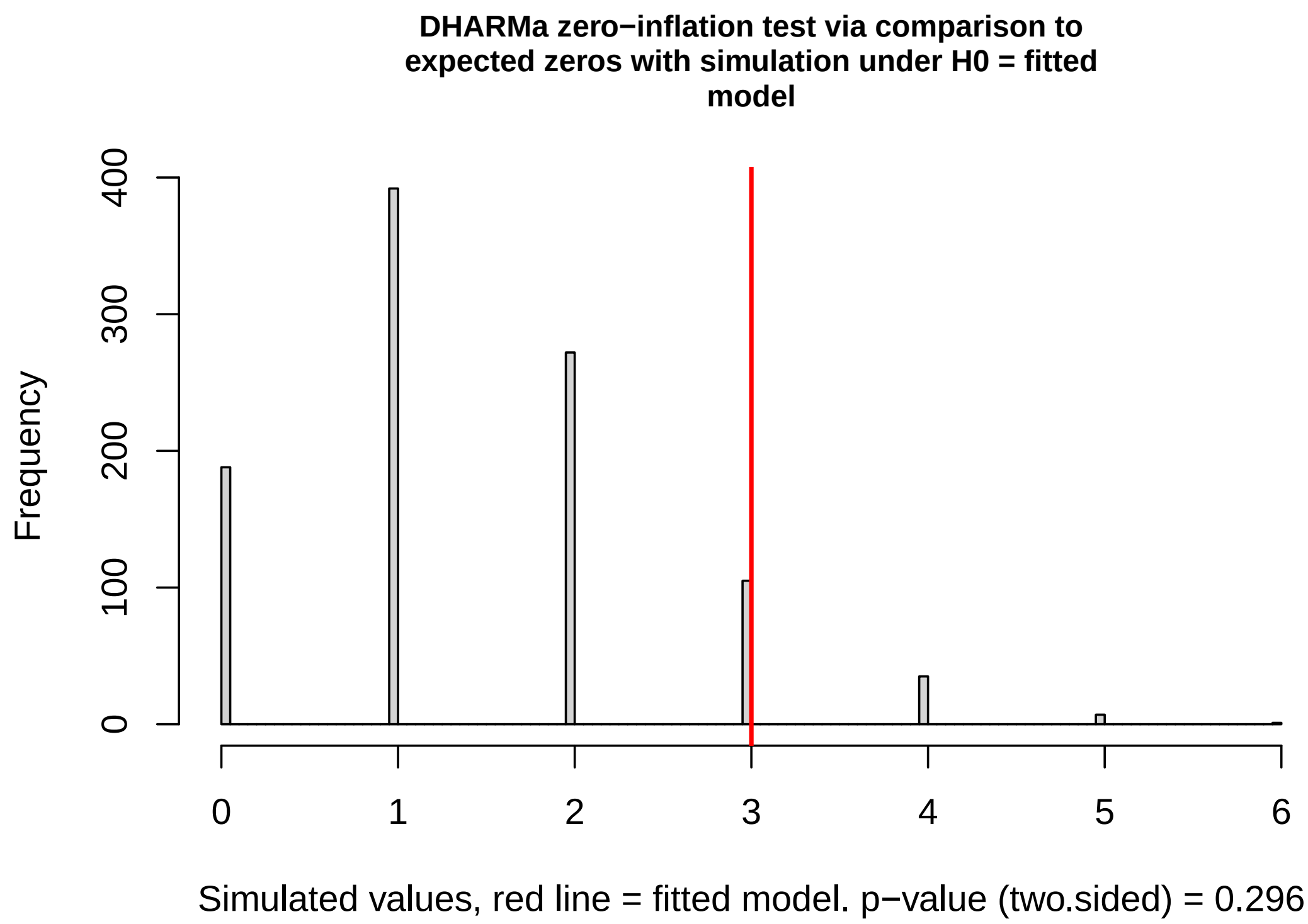


```
##
##  DHARMa zero-inflation test via comparison to expected zeros with
##  simulation under H0 = fitted model
##
## data:  simulationOutput
## ratioObsSim = 2.095, p-value = 0.296
## alternative hypothesis: two.sided
```

```
testOutliers(res_select_beta)
```

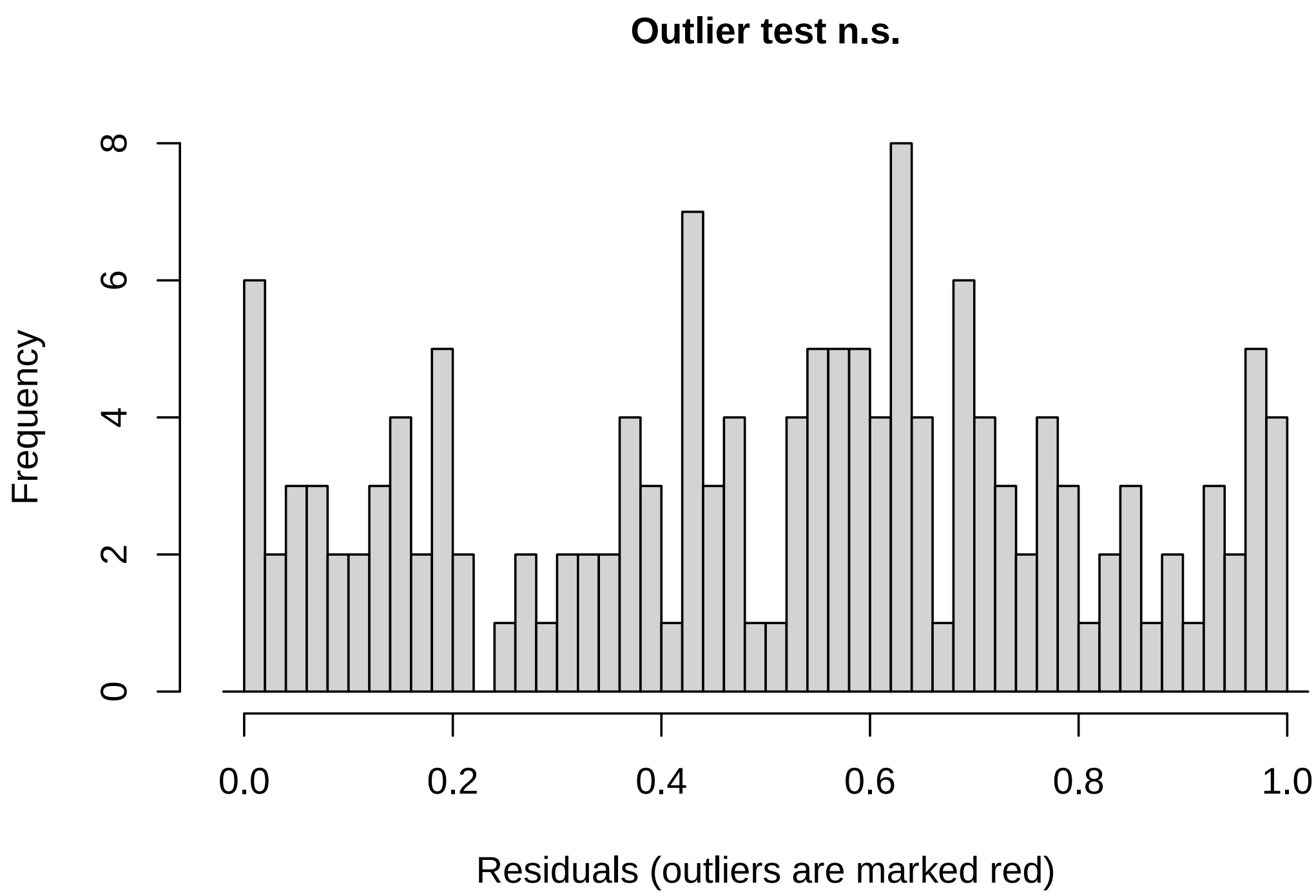


```
##
##  DHARMa bootstrapped outlier test
##
## data:  res_select_beta
## outliers at both margin(s) = 0, observations = 150, p-value = 1
## alternative hypothesis: two.sided
##  percent confidence interval:
##  0.000000000 0.006666667
## sample estimates:
## outlier frequency (expected: 0.000933333333333333 )
##                                                     0
```

6.3 Selected beta-binomial model

```
summary(m_select_beta)
```

```
##  Family: betabinomial  ( logit )
## Formula:
## cbind(Bites_HNT, Bites_LNT) ~ NH3_umol + DaysSinceStart_c + (1 |      Fish)
## Data: selectivity_trial
##
##      AIC       BIC    logLik -2*log(L)  df.resid
##   1214.3    1229.3    -602.1    1204.3       145
##
## Random effects:
##
```

```
## Conditional model:
##  Groups Name        Variance Std.Dev.
##  Fish   (Intercept) 0.06135  0.2477
## Number of obs: 150, groups:  Fish, 21
##
## Dispersion parameter for betabinomial family (): 3.98
##
## Conditional model:
##                   Estimate Std. Error z value Pr(>|z|)
## (Intercept)        0.96391    0.14519   6.639 3.16e-11 ***
## NH3_umol           0.03192    0.03869   0.825    0.409
## DaysSinceStart_c   0.01326    0.01654   0.802    0.423
## ---
## Signif. codes:  0 '***' 0.001 '**' 0.01 '*' 0.05 '.' 0.1 ' ' 1
```

6.4 Odds ratios and HNT allocation probability

```r
selectivity_coef <- as.data.frame(
  summary(m_select_beta)$coefficients$cond
) %>%
  tibble::rownames_to_column("Term") %>%
  rename(
    Estimate = Estimate,
    SE = `Std. Error`,
    z_value = `z value`,
    p_value = `Pr(>|z|)`
  ) %>%
  mutate(
    CI_low_logit = Estimate - 1.96 * SE,
    CI_high_logit = Estimate + 1.96 * SE,
    Odds_ratio = exp(Estimate),
    OR_CI_low = exp(CI_low_logit),
    OR_CI_high = exp(CI_high_logit)
  )

selectivity_coef
```

```
##               Term   Estimate         SE   z_value      p_value CI_low_logit
## 1      (Intercept) 0.96390915 0.14518818 6.6390333 3.157472e-11   0.67934032
## 2         NH3_umol 0.03191810 0.03868885 0.8249948 4.093745e-01  -0.04391204
## 3 DaysSinceStart_c 0.01325955 0.01653988 0.8016716 4.227430e-01  -0.01915862
##   CI_high_logit Odds_ratio OR_CI_low OR_CI_high
## 1    1.24847798   2.621926 1.9725760   3.485035
## 2    0.10774824   1.032433 0.9570381   1.113767
## 3    0.04567773   1.013348 0.9810237   1.046737
```

```r
selectivity_intercept_probability <- selectivity_coef %>%
  filter(Term == "(Intercept)") %>%
  transmute(
    Probability_HNT = plogis(Estimate),
    Probability_CI_low = plogis(CI_low_logit),
```

```
    Probability_CI_high = plogis(CI_high_logit)
  )

selectivity_intercept_probability
```

```
##   Probability_HNT Probability_CI_low Probability_CI_high
## 1       0.7239038          0.6635914           0.7770363
```
