# Supplementary material for: Food selectivity and grazing responses of the Lawnmower Blenny (Salarias fasciatus) to nutrient enrichment and ammonia exposure
Source: J Fish Biol. 2026 Sep 22;109(5):2851–62. doi: 10.1111/jfb.70659 (PMC13770696; doi:10.1111/jfb.70659)
Supplement: Supplementary file 2 [file JFB-109-2851-s001.docx]

# Supplementary tables and Figures

Supplementary Table S1. Mean, standard deviation (SD), and standard error (SE) of water quality parameters monitored in the individual tanks from the start of fish acclimation (11 April 2023) to the end of the experiment (11 May 2023). Parameters include nitrite concentration (NO₂⁻), salinity, temperature (T), oxygen saturation (%), dissolved oxygen concentration (O₂), and pH.

| Variable | Mean | SD | SE |
| --- | --- | --- | --- |
| NO_2_ [µM] | 1.382258 | 1.096178 | 0.049775 |
| Salinity [] | 35.07588 | 0.228972 | 0.010397 |
| T [°C] | 23.99918 | 0.722253 | 0.032796 |
| %O_2_ saturation | 100.7412 | 1.953566 | 0.088707 |
| O_2_ [mg L^-1^] | 6.88715 | 0.086076 | 0.003908 |
| pH | 7.987131 | 0.052153 | 0.002368 |

**a**

**b**

Figure S1. Experimental setup used for the grazing activity trials at the Marine Ecology Experimental Facility (MAREE), Leibniz Centre for Tropical Marine Research (ZMT). (a) Overview of the individual fish tanks and camera arrangement used to record grazing behaviour during the experiment. (b) Close-up of a single experimental tank showing one Salarias fasciatus individual, the two experimental tiles, the PVC shelter, and the air stone.
